# BASS: multi-scale and multi-sample analysis enables accurate cell type clustering and spatial domain detection in spatial transcriptomic studies

**DOI:** 10.1186/s13059-022-02734-7

**Published:** 2022-08-04

**Authors:** Zheng Li, Xiang Zhou

**Affiliations:** 1 Department of Biostatistics, University of Michigan, Ann Arbor, MI 48109 USA; 2 Center for Statistical Genetics, University of Michigan, Ann Arbor, MI 48109 USA

**Keywords:** Spatial transcriptomics, Multi-scale analysis, Clustering analysis, Cell type, Spatial domain, Multi-sample analysis, Tissue section, Bayesian hierarchical model, BASS

## Abstract

Spatial transcriptomic studies are reaching single-cell spatial resolution, with data often collected from multiple tissue sections. Here, we present a computational method, BASS, that enables multi-scale and multi-sample analysis for single-cell resolution spatial transcriptomics. BASS performs cell type clustering at the single-cell scale and spatial domain detection at the tissue regional scale, with the two tasks carried out simultaneously within a Bayesian hierarchical modeling framework. We illustrate the benefits of BASS through comprehensive simulations and applications to three datasets. The substantial power gain brought by BASS allows us to reveal accurate transcriptomic and cellular landscape in both cortex and hypothalamus.

**Supplementary Information:**

The online version contains supplementary material available at 10.1186/s13059-022-02734-7.

## Background

Spatially resolved transcriptomic studies are enabled by various recently developed spatial transcriptomic technologies that perform gene expression profiling with spatial localization information on tissues [1–3]. These technologies are based on either high-throughput sequencing or single molecular fluorescent in situ hybridization (smFISH) [4] and are achieving increasingly high spatial resolution. For example, among the sequencing-based technologies, the old Spatial Transcriptomics (ST) technology measures gene expression on multiple capture sites known as spots, each of which has a diameter of 100 μm and captures mRNA from a neighborhood of likely 10–40 single cells [5]. The 10x Visium technology improves upon ST technology to yield a spatial resolution of 55 μm with each measured spot assaying 1–10 cells. Slide-seq [6] and the subsequent Slide-seqV2 [7] reach a spatial resolution of 10 μm, providing near-cellular observation with each measured spot containing 1–3 cells. HDST [8] and Seq-Scope [9] further achieve a spatial resolution of 2 and 0.6 μm, respectively, allowing for the measurement of transcripts at a sub-cellular resolution. Moreover, some recent sequencing-based technologies, such as STARmap [10] and FISSEQ [11], are based on in situ sequencing (ISS) and can directly measure gene expression at the single-cell resolution. The upcoming 10x Visium HD technology will also extend the commercial 10x Visium to single-cell spatial resolution. Besides the sequencing-based technologies, the smFISH-based technologies, such as MERFISH [12], seqFISH [13], seqFISH+ [14], and osmFISH [15], can also obtain gene expression measurements directly at single-cell resolution.

The abundant availability of spatial transcriptomic datasets with single-cell or approximate single-cell resolution provides rich information for comprehensive characterization of the spatial transcriptomic landscape of complex tissues at multiple anatomic scales. Specifically, at the single-cell scale, single-cell resolution spatial transcriptomics enables clustering of cells into distinct cell types [10, 16]. Cell type clustering allows for the characterization of the spatial distribution of distinct cell types, facilitating the investigation of cell-cell interactions across spatial locations [17, 18] and the detection of genes with spatial expression patterns across cells [19–22]. At the tissue domain scale, single-cell resolution spatial transcriptomics contains crucial information for detecting distinct spatial domains on the tissue [23–25]. Spatial domain detection allows for the transcriptomic characterization of tissue structures, facilitating the accurate assessment of cell type composition across tissue locations and the evaluation of the transcriptomic profiles of tissue microenvironments [23, 26]. These two key analytic tasks at the two distinct anatomic scales—cell type clustering at the single-cell scale and spatial domain detection at the tissue domain scale—are enabled by multiple recently developed computational methods. Specifically, for cell type clustering, existing analysis of single-cell resolution spatial transcriptomics primarily relies on clustering methods developed for single-cell RNA sequencing (scRNA-seq) datasets. For example, Seurat [27] and SC3 [28] are two common cell type clustering approaches that enjoy robust performance across a range of scRNA-seq settings [29, 30] and that have been applied to analyze single-cell resolution spatial transcriptomics [9]. In addition, tailored cell type clustering methods for single-cell resolution spatial transcriptomics are also being developed. For example, FICT [31] is a recent method that can combine both gene expression and cell localization information available in spatial transcriptomics for enhanced cell type clustering. For spatial domain detection, common analytic methods include the hidden Markov random field (HMRF) [23], BayesSpace [25], and SpaGCN [24], to name a few. These methods allocate tissue locations into distinct spatial domains while properly accounting for the spatial correlation among locations. For example, both HMRF and BayesSpace rely on a Potts model to impose a spatial dependency structure among neighboring tissue locations, while SpaGCN relies on a graph convolutional network to model such spatial dependency. Overall, these recently developed computational methods facilitate cell type clustering and spatial domain detection in spatial transcriptomics, allowing us to characterize the unique cell type composition underneath each spatial domain and investigate the cellular and transcriptomic mechanisms that underlie tissue function and malfunction [32–34].

Despite the importance of cell type clustering and spatial domain detection, existing methods for these analytic tasks have two important limitations. First, all existing methods carry out only one of the two analytic tasks, effectively rendering the analysis at the two different anatomic scales disconnected from each other. However, cell type clustering and spatial domain detection are inherently interconnected analytic tasks, with the results obtained at one analysis potentially facilitating the other. In particular, knowing the cell type assignments can help better characterize the cell type composition across tissue locations, thus facilitating the detection of spatial domains characterized by distinct cell type compositions. Conversely, knowing the allocation of spatial domains provides crucial information on how cells and cell types are segregated across spatial locations, thus facilitating cell type inference and clustering. Second, all existing methods have focused on analyzing spatial transcriptomic data collected from a single tissue section. However, spatial transcriptomic studies often collect multiple adjacent sections from the same tissue or collect tissue samples from multiple individuals. Different sections from the same or similar tissues may contain a similar set of spatial domains and a similar composition of cell types. Consequently, modeling multiple tissue sections/samples together can borrow information across samples to potentially enhance the performance of both cell type clustering and spatial domain detection for single-cell spatial transcriptomics.

Here, we present a new computational method, BASS (Bayesian Analytics for Spatial Segmentation), for multi-scale and multi-sample analysis that overcomes the above two limitations. BASS performs multi-scale transcriptomic analyses in the form of joint cell type clustering and spatial domain detection, with the two analytic tasks carried out simultaneously within a Bayesian hierarchical modeling framework. For both analyses, BASS properly accounts for the spatial correlation structure and seamlessly integrates gene expression information with spatial localization information to improve their performance. In addition, BASS is capable of multi-sample analysis that jointly models multiple tissue sections/samples, facilitating the integration of spatial transcriptomic data across tissue samples. Note that the multi-section analysis of BASS only requires the spatial transcriptomic data to contain multiple tissue sections. For spatial transcriptomic data that contain only one tissue section, one can directly apply the single-section analysis of BASS. We illustrate the benefits of BASS through extensive simulations and in-depth analyses of three spatial transcriptomic datasets that include two with single-cell resolution and one from the 10x Visium platform.

## Results

### Simulation results

**Fig. 1:**
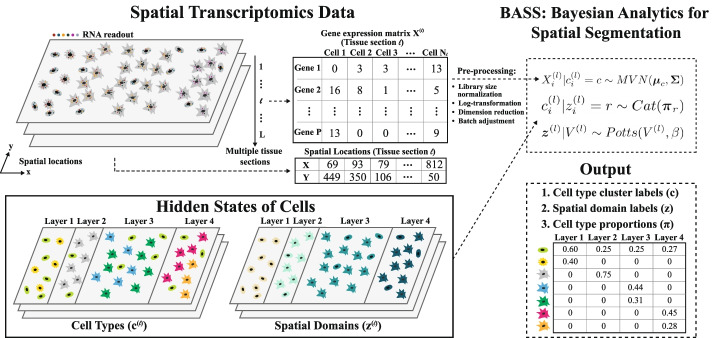
Workflow of BASS. BASS performs multi-scale and multi-sample analysis for accurate cell type clustering and spatial domain detection in spatial transcriptomics. BASS takes input of the gene expression matrix and spatial location information from multiple tissue sections and models both information in a hierarchical Bayesian framework. In the analysis, BASS treats the cell type label (c) and the spatial domain label (z) for each cell on the tissue as latent/hidden variables and infers them through an efficient inference algorithm. After analysis, BASS provides the inferred cell type cluster labels, the spatial domain labels, and the cell type proportions inside each spatial domain as outputs

A method schematic of BASS is shown in Fig. 1, with details provided in the “Methods” section. We conducted extensive simulations to evaluate the performance of BASS and compared it with existing approaches. Simulation details are provided in “Methods”. Briefly, for single tissue section simulations, we obtained the spatial location information for cells from a tissue section of the STARmap data and allocated them into four distinct cortical layers. We assumed that each spatial domain consisted of multiple cell types, and we set the number of cell types to be four. We examined four different scenarios (I–IV) by varying the composition of cell types in different spatial domains and simulated gene expression for each cell using the splatter package. We explored nine simulation settings for each scenario by varying the number of genes, the proportion of genes that were differentially expressed (DE) in each cell type versus the others, and the DE gene strength. We applied BASS, HMRF, BayesSpace, and SpaGCN for spatial domain detection. Because HMRF requires users to input a value for its spatial parameter *β*, we explored a range of *β* values and display HMRF for three different *β*s that correspond to the worst, median, and best performance. We refer to the HMRF with the best performance as the oracle version, as the spatial parameter *β* is selected based on the true spatial domains that are assumed to be unknown for all other methods. In addition, we applied BASS, Seurat, SC3, and FICT for cell type clustering. Because only BASS can perform both cell type clustering and spatial domain detection, we also applied BASS to estimate the cell type compositions across spatial domains. Besides single tissue section simulations, we generated additional tissue sections by introducing a moderate amount of shift on the spatial domain boundaries between the four cortical layers (Additional file 1: Fig. S2). We then evaluated the performance of BASS for the aforementioned tasks in the integrative analysis of multiple tissue sections. In addition, we examined the performance of Seurat and SC3 for cell type clustering in the multiple tissue section analysis. Besides the main simulations described above, we also explored various other factors including the specified number of cell types/spatial domains, rare cell types, and a random exclusion of genes, on the performance of different methods.

#### Main simulations

**Fig. 2:**
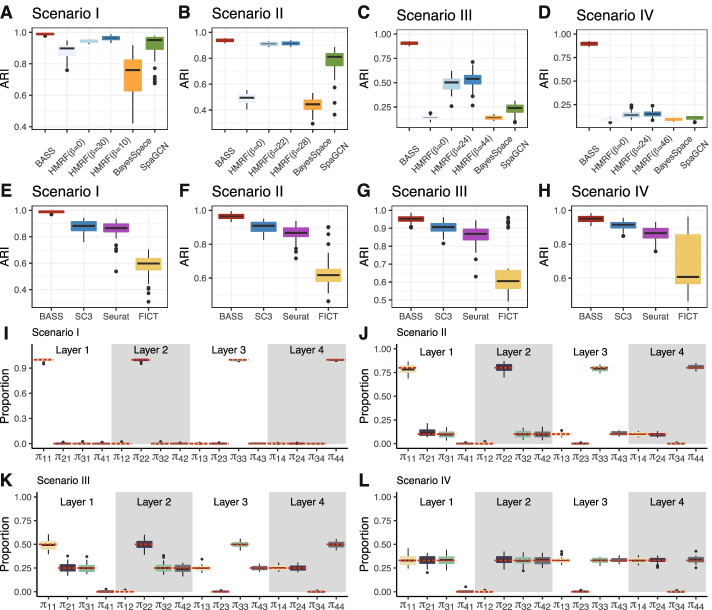
Comparison of different methods for spatial domain detection and cell type clustering in simulations on a single tissue section. Boxplots of ARI show the accuracy of different methods for (**A–D**) spatial domain detection and (**E–H**) cell type clustering. Compared methods for spatial domain detection include BASS, HMRF, BayesSpace, and SpaGCN. For HMRF, a list of the spatial parameter *β*s ranging from 0 to 50 at increments of 2 were examined, and the three *β* values that corresponded to the worst, median, and best performance are displayed. Compared methods for cell type clustering include BASS, Seurat, SC3, and FICT. **I–L** Boxplots show the estimated cell type proportions in each spatial domain across simulation replicates, where *π*_*cr*_ indicates the proportion of cell type *c* in the spatial domain *r*. The red dashed lines indicate the true proportions. Simulations were conducted under Scenario I (**A, E, I**), Scenario II (**B, F, J**), Scenario III (**C, G, K**), or Scenario IV (**D, H, L**), with the simulation parameters set as the baseline setting: the number of genes (*nGenes*) = 200, the proportion of genes that were differentially expressed in each cell type versus the others (*de*. *prob*) = 0.2, and the DE gene strength (*de*. *facloc*) = 1.1

First, we evaluated the performance of different methods for spatial domain detection on a single tissue section. In the simulations, we found that BASS outperformed HMRF, BayesSpace, and SpaGCN across all scenarios (Fig. 2A–D). Specifically, the performance of BASS was generally followed by the oracle version of HMRF, SpaGCN, and BayesSpace. The performance of BayesSpace was relatively poor, presumably because BayesSpace was developed for analyzing the ordered lattice structure of spots from ST and 10x Visium platforms and is likely not well suited to analyze data with relatively unordered cell locations. The advantage of BASS was especially apparent when the spatial domains consisted of multiple cell types (Fig. 2C,D), highlighting the benefits of performing multi-scale analyses and incorporating cell type information for detecting spatial domains. In addition, the advantage of BASS was also more apparent in the realistic scenario where there were multiple dominant cell types in each spatial domain as compared to the simplistic scenario where one cell type dominated each domain. For example, in scenario I where one cell type completely dominated one spatial domain, BASS, the oracle version of HMRF, BayesSpace, and SpaGCN achieved a median ARI of 0.99, 0.97, 0.76, and 0.95 (Fig. 2A). In contrast, in scenario IV where the cell types in each spatial domain had equal compositions, BASS achieved a median ARI of 0.90 while the oracle version of HMRF, BayesSpace, and SpaGCN only achieved a median ARI of 0.15, 0.09, and 0.11 (Fig. 2D). In addition, we found that the *β* value for the oracle version of HMRF differed, sometimes quite substantially, under different simulation settings. For example, the optimal value of *β* ranged from 8 to 50 in scenario I (Additional file 1: Fig. S3) and ranged from 36 to 50 in scenario III (Additional file 1: Fig. S5). Similarly, the *β* estimates from BASS varied both within and across different simulation settings (Additional file 1: Fig. S14). In scenario I, the *β* estimates were larger than those in the other scenarios, which was consistent with the relatively high spatial correlation in scenario I induced by homogenous cell type composition in each spatial domain as compared to the relatively low spatial correlation in the other scenarios due to the heterogenous cell type compositions in each domain. Note that while both HMRF and BASS use the Potts model that contains the interaction parameter *β*, the *β* parameter is not directly comparable between the two methods due to the additional hierarchical modeling components introduced in BASS. These results suggest that the optimal *β* is highly data-dependent and highlight the importance of estimating the optimal *β* in each data set as is done in BASS. As expected, the performance of all methods increased as the number of genes increased, as the DE gene strength increased, and as the proportion of genes that were differentially expressed in each cell type versus the others increased, though the relative performance of different methods remained largely similar (Additional file 1: Figs. S3-S6). Importantly, BASS is relatively robust with respect to the DE gene strength and the proportion of DE genes, both of which have substantial impact on the performance of HMRF, BayesSpace, and SpaGCN. For example, in scenario II, when DE gene strength (determined by the *de*. *facloc* parameter) reduced from 1.4 to 0.5, the median ARI of the oracle version of HMRF, BayesSpace, and SpaGCN decreased from 0.92, 0.53, and 0.85 to 0.60, 0.02, and 0.10, respectively (Additional file 1: Fig. S4E vs Fig. S4H). In contrast, the median ARI of BASS remained high and reduced only from 0.94 to 0.90 (Additional file 1: Fig. S4E vs Fig. S4H). These results highlight the accurate performance of BASS on detecting spatial domains.

Next, we evaluated the performance of different methods for cell type clustering on a single tissue section. In the simulations, we found that BASS outperformed SC3, Seurat, and FICT across all scenarios (Fig. 2E–H). The advantage of BASS over the other three methods was especially apparent when cell types displayed spatial patterns on the tissue. For example, in scenario I where cell types displayed strong spatial patterns, BASS achieved a median ARI of 0.99 across simulation replicates, while the median ARI from SC3, Seurat, and FICT were 0.88, 0.87, and 0.60, respectively (Fig. 2E). In contrast, in scenario IV where cell types displayed weak spatial patterns, BASS achieved a median ARI of 0.95, while the median ARI from SC3, Seurat, and FICT were 0.92, 0.87, and 0.61, respectively (Fig. 2H). The higher performance gain by BASS in the presence of spatial patterns highlights the benefits of performing multi-scale analyses and incorporating spatial domain information for clustering cell types. The performance gain by BASS was especially apparent in the challenging setting where the degree of separability between cell types was relatively small, as reflected by a relatively small DE gene strength (Additional file 1: Fig. S7E vs Fig. S10E), small number of genes (Additional file 1: Fig. S7A vs Fig. S10A), or small proportion of DE genes (Additional file 1: Fig. S7I vs Fig. S10I). Surprisingly, FICT did not perform as well as SC3 and Seurat, and its performance remained similar regardless of the strength of the spatial correlation pattern of the cell types (Fig. 2E–H). The low performance of FICT is likely due to the large number of parameters employed in the model, which reduces the degree of freedom and subsequently clustering accuracy, especially when the number of cells in the data is small or when each cell type consists of a heterogeneous cell population. Indeed, FICT could outperform SC3 and Seurat when the number of genes, proportion of DE genes, or DE gene strength was relatively large (Additional file 1: Fig. S9B-D, H, K). As expected, the performance of all methods increased with increasing number of genes, DE gene strength, and proportion of DE genes, though the relative performance of different methods remained largely similar except for FICT as described above (Additional file 1: Figs. S7-S10). Comparing SC3 and Seurat, we found that SC3 always outperformed Seurat (Fig. 2E–H and Additional file 1: Figs. S7-S10), consistent with earlier observations [28]. Overall, BASS is effective for cell type clustering, and, when paired with its effectiveness in spatial domain detection, can lead to accurate cell type composition estimation across spatial domains (Fig. 2I–L and Additional file 1: Figs. S11-S13).

**Fig. 3:**
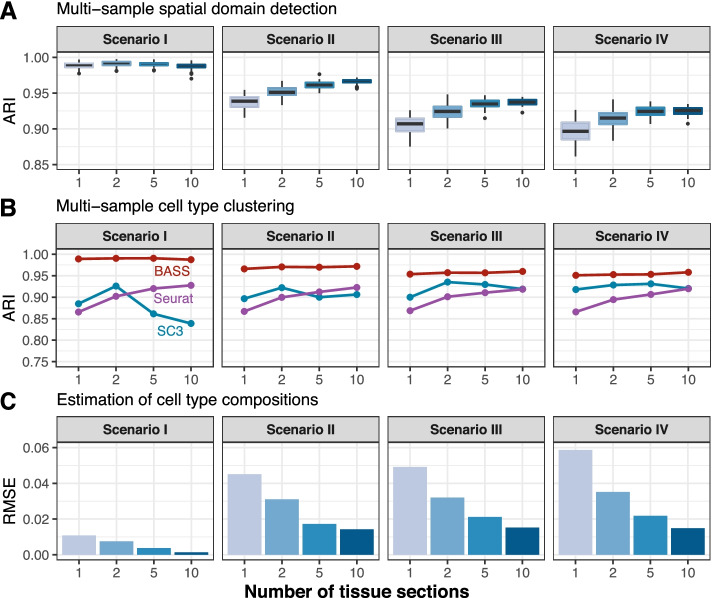
Comparison of different methods for spatial domain detection and cell type clustering in simulations with multiple tissue sections. **A** Boxplots of ARI show the accuracy of BASS for spatial domain detection (*y*-axis) in the presence of 1, 2, 5, or 10 tissue sections (*x*-axis). **B** Line plots display the median ARI by different methods for cell type clustering across 50 simulation replicates (*y*-axis) in the presence of 1, 2, 5, or 10 tissue sections (*x*-axis). Compared methods for cell type clustering include BASS, Seurat, and SC3. **C** Barplots show the median RMSE between the estimated cell type compositions and the true compositions across 50 simulation replicates (*y*-axis) in the presence of 1, 2, 5, or 10 tissue sections (*x*-axis). Simulations were conducted under Scenario I, Scenario II, Scenario III, and Scenario IV, with the simulation parameters set as the baseline setting: the number of genes (*nGenes*) = 200, the proportion of genes that were differentially expressed in each cell type versus the others (*de*. *prob*) = 0.2, and the DE strength (*de*. *facloc*) = 1.1

Finally, we evaluated the performance of BASS on integrative analysis of multiple tissue sections. Across all simulation scenarios, we found that the performance of BASS for all three analytic tasks, including spatial domain detection, cell type clustering, and estimation of cell type compositions across domains, increased with the increasing number of analyzed tissue sections (Fig. 3 and Additional file 1: Fig. S15). For example, the performance gain of BASS on using five tissue sections versus a single section was 3, 6, and 55% for the three analytic tasks, respectively in scenario III (Additional file 1: Fig. S15). For cell type clustering, the performance of Seurat also increased with increasing number of analyzed tissue sections, while the performance of SC3 increased first, but then decreased with increasing number of tissue sections (Fig. 3B). The dependence of SC3 on the number of tissue sections is expected as SC3 performs an initial clustering on a fixed number of randomly selected cells (= 5000), which likely imposes a potential upper limit for its performance in large datasets [35]. Similar to the single tissue section analysis, the advantage of BASS over the other methods on cell type clustering was especially apparent when cell types displayed spatial patterns on the tissue (Fig. 3B and Additional file 1: Fig. S15B).

#### Influence of the specified number of cell types/spatial domains

First, we evaluated the influence of the specified number of cell types on cell type clustering. As expected, when the number of cell types was under-specified, all methods aggregated cells of multiple types into the same clusters and produced lower ARIs than before (median ARIs = 0.36, 0.41, 0.31, and 0.37 for BASS, SC3, Seurat, and FICT; Additional file 1: Fig. S16A-C). On the other hand, when the number of cell types was over-specified, we found that BASS could still accurately assign cells of the same type into the same cluster while keeping the redundant clusters to be of minimal sizes, leading to only a small ARI loss (Additional file 1: Fig. S16A, C). For example, when the number of cell types was specified to be 10 (truth = 4), the median ARI of cell type clustering from BASS was 0.90, which represented only a small reduction compared to the median ARI of 0.95 when the number of cell types was specified to be the truth (4) (Additional file 1: Fig. S16A). In this case, the top 4 clusters with the largest number of cells (Clusters 1–4) corresponded to the 4 cell types, while the remaining 6 clusters were small and consisted of only an average of 6% of the total cell population (Additional file 1: Fig. S16C). In contrast, SC3, Seurat, and FICT segregated cells of the same type into multiple clusters and produced much poorer ARIs than before. Specifically, in the same example above, the median ARIs from SC3, Seurat, and FICT were 0.53, 0.53, and 0.45, respectively, which were much worse compared to the median ARIs of 0.91, 0.87, and 0.61 when the number of cell types was specified to be the truth (4). For these three methods, the 6 clusters that did not clearly correspond to the true cell types were large and consisted of an average of 30, 41, and 54% of cells, respectively (Additional file 1: Fig. S16C).

Next, we evaluated the influence of the specified number of spatial domains on spatial domain detection. As expected, when the number of spatial domains was under-specified, all methods tended to merge different spatial domains into large ones and produced poorer ARIs than before (median ARIs = 0.52, 0.11, 0.03, and 0.14 for BASS, HMRF, BayesSpace, and SpaGCN; Additional file 1: Fig. S17A). On the other hand, when the number of spatial domains was over-specified, similarly to what we have observed in the cell type clustering, BASS could still accurately detect the 4 spatial domains while keeping the redundant domains to be small and contained only a small proportion of cells, resulting in only a small reduction in ARI (Additional file 1: Fig. S17A-C). HMRF was also relatively robust to the over-specification of the number of spatial domains, presumably because we explored a list of spatial parameters *β* in HMRF and chose the oracle version of HMRF that likely over-fitted the data. In contrast, BayesSpace and SpaGCN separated one tissue domain into multiple domains, leading to a substantial loss in ARI (Additional file 1: Fig. S17C).

Finally, we evaluated the influence of the specified number of cell types on spatial domain detection in BASS (Additional file 1: Fig. S16D-F) and the influence of the specified number of spatial domains on cell type clustering in BASS (Additional file 1: Fig. S17D-F). We found that a mis-specified number of spatial domains, regardless of whether it was under-specified or over-specified, did not have much influence on the performance of cell type clustering. An over-specified number of cell type clusters did not influence the performance of spatial domains either. Only an under-specified number of cell type clusters reduced the performance of spatial domain detection, which is presumably due to the relatively poor cell type clustering performance there. Consistent results are obtained using the normalized mutual information (NMI) as evaluation criterion, which accounts for the difference between the estimated number of clusters/domains and the true number (Additional file 1: Fig. S18).

#### Influence of rare cell types

We evaluated the performance of all methods in the presence of rare cell types in the simulation. We found that the performance of all methods decreased when we increased the number of rare cell types from six to ten, although the performance of BASS remained much better than the other methods (Additional file 1: Fig. S19). For example, when the rare cell types were randomly distributed across the entire tissue, the median *F*_1_ scores (MCCs) of BASS, SC3, Seurat, and FICT decreased from 0.78 (0.78), 0.63 (0.63), 0.57 (0.55), and 0.40 (0.37) to 0.51 (0.50), 0.40 (0.39), 0.35 (0.34), and 0.27 (0.25), respectively, when the number of rare cell types increased from six to ten. On the other hand, the median ARI from BASS only slightly decreased from 0.84 to 0.76 when the number of rare cell types increased from six to ten, indicating that BASS can still accurately detect the major cell types and that the ARI is not an appropriate metric for evaluating the clustering of rare cell types. In contrast, the median ARI from SC3, Seurat, and FICT decreased substantially from 0.65, 0.56, and 0.43 to 0.34, 0.43, and 0.35, indicating their performance on detecting major cell types was substantially impacted by the presence of rare cell types. In addition, the performance of BASS on detecting rare cell types improved when rare cell types exhibit a domain-specific pattern (Additional file 1: Fig. S19). For example, when the number of rare cell types was set to be ten, the median *F*_1_ score (MCCs) of BASS increased from 0.51 (0.50) to 0.59 (0.59) when the distribution patterns of rare cell types changed from random to domain specific. In contrast, the performance of SC3, Seurat, and FICT remained largely similar. Finally, we found that the performance of all methods on detecting spatial domains remained largely similar regardless of the number of rare cell types and slightly increased when rare cell types exhibit domain-specific distribution patterns, presumably because spatial domains are largely characterized by major cell types and thus their detection is not influenced much by the presence of rare cell types (Additional file 1: Fig. S20).

#### Influence of a random exclusion of genes

We conducted simulations in which we randomly excluded genes from the gene expression matrix and evaluated its influence on the performance of all methods. As expected, for both cell type clustering and spatial domain detection, the performance of all methods decreased when fewer genes were retained in the expression matrix, though the performance of BASS remained better than the other methods. For cell type clustering, the performance of BASS was also less affected by a random exclusion of genes than the other methods (Additional file 1: Fig. S21A). For example, in scenario III, the median ARI of SC3, Seurat, and FICT decreased substantially from 0.92, 0.88, and 0.93 to 0.27, 0.34, and 0.40 while the median ARI of BASS decreased from 0.97 to 0.74 when the number of retained genes decreased from 500 to 200. In addition, the performance of BASS on cell type clustering was more robust to the random exclusion of genes in scenarios I and II than in scenarios III and IV while the performance of SC3, Seurat, and FICT remained largely similar across the four scenarios, suggesting that BASS has advantages over the other three methods on detecting cell types that display strong spatial patterns (Additional file 1: Fig. S21A). For spatial domain detection, we found that both BASS and the oracle version of HMRF were more robust to a random exclusion of genes than BayesSpace and SpaGCN (Additional file 1: Fig. S21B). For example, in scenario III, when the number of retained genes decreased from 500 to 200, the performance reduction in terms of ARI was 2% and 20% for BASS and HMRF while the performance reduction was 88% and 63% for BayesSpace and SpaGCN. We also noticed that the performance of BASS on spatial domain detection varied a lot in scenarios III and IV compared to scenarios I and II (Additional file 1: Fig. S21B). Presumably, this is because spatial domains cannot be accurately detected when cell types are not well clustered, which is more likely to occur in scenarios III and IV as mentioned above.

### Mouse medial prefrontal cortex data by STARmap

**Fig. 4:**
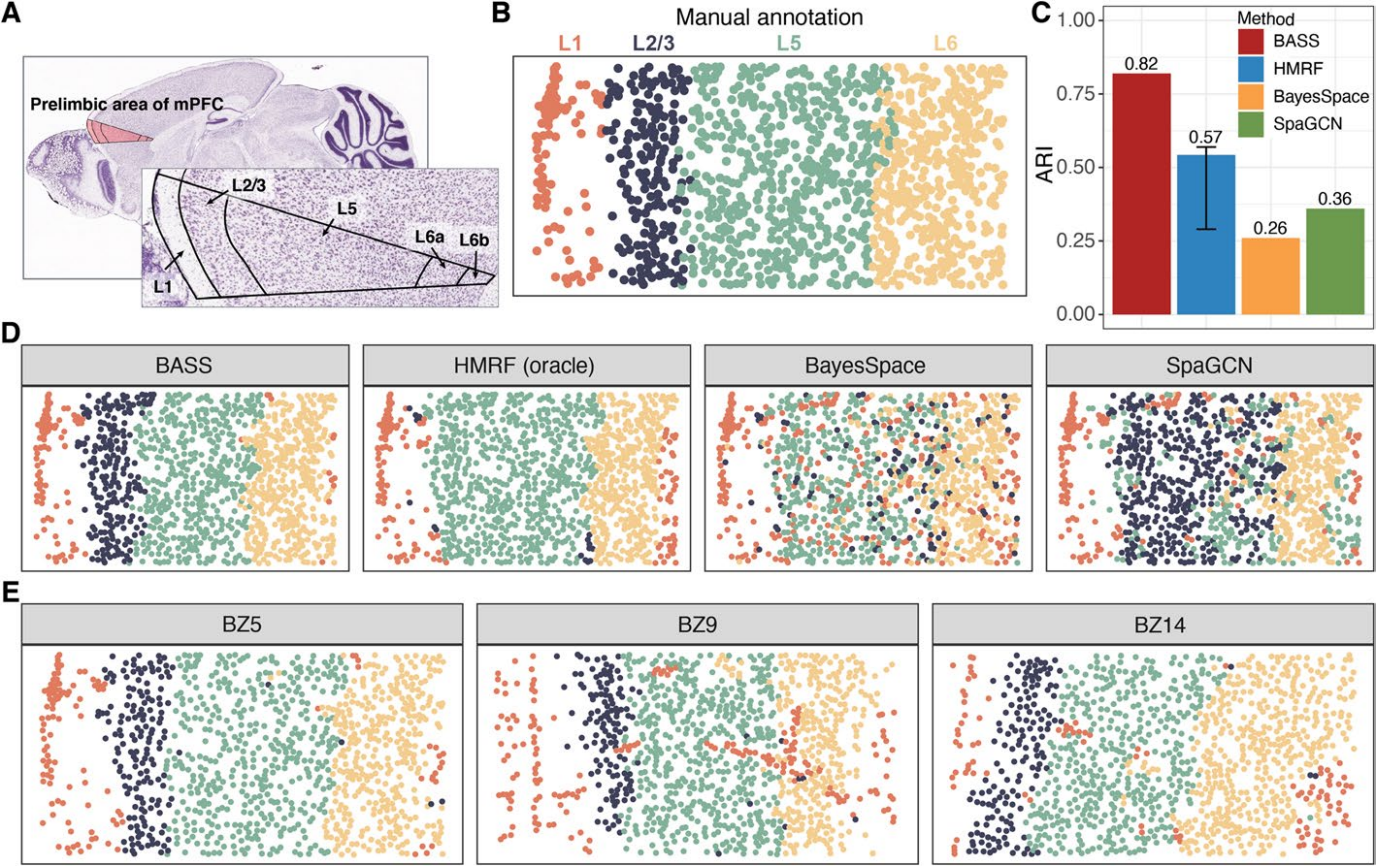
Detecting spatial domains in the STARmap dataset. **A** An anatomic reference atlas obtained from the Allen Mouse Brain Atlas displays the spatial domains of the prelimbic area in the mouse prefrontal cortex. **B** Annotated spatial domain labels for the tissue section BZ5 based on spatial gene expression patterns and the Allen Mouse Brain Atlas. **C** Barplots of ARI show the accuracy of different methods for spatial domain detection on the tissue section BZ5. The compared methods include BASS, HMRF, BayesSpace, and SpaGCN. For HMRF, the range of ARI based on a pre-defined list of *βs* is shown with an error bar. **D** The identified spatial domains on the tissue section BZ5 are shown for BASS, HMRF, BayesSpace, and SpaGCN. **E** The identified spatial domains on three tissue sections (BZ5, BZ9, and BZ14) were obtained with the multi-sample analysis of BASS

We applied BASS to analyze two publicly available spatial transcriptomic data with single-cell resolution. The first dataset is from the STARmap technology, consisting of three tissue sections obtained from the medial prefrontal cortex (mPFC) of the mouse brain from different mice. mPFC is a crucial cortical region located at the front of the frontal lobe and plays an essential role in high-level cognitive functions including decision-making, memory, attention, and emotion [36]. Dysfunction of the mPFC has been found in various neurological and psychiatric disorders such as depression and Alzheimer’s disease [37]. The mPFC is comprised of four layers (L1, L2/3, L5, and L6) and consists predominantly of excitatory pyramidal neurons (about 80~90%) and inhibitory GABAergic interneurons (about 10~20%), orchestrating cortical network dynamics and communicating with long-distance targets [37]. The three tissue sections include BZ5 (1049 cells), BZ9 (1053 cells), and BZ14 (1088 cells), with expression measurements collected on a common set of 166 genes. The cells on all tissue sections were carefully annotated to four distinct layer structures that included L1, L2/3, L5, and L6 (Fig. 4A,B and Additional file 1: Fig. S23). We performed single-section analysis on tissue section BZ5 and multiple-section analysis on all three tissue sections.

We first examined the results from different methods on spatial domain detection. In the analysis, BASS detected four spatial domains that highly resemble the expected cortical layers (ARI = 0.82; Fig. 4C,D). In contrast, the spatial domains detected by HMRF (ARI = 0.57), BayesSpace (ARI = 0.26), and SpaGCN (ARI = 0.36) do not generally match the underlying truth (Fig. 4C,D), with the ranking of the method generally consistent with that observed in the simulations. A close look at the detected spatial domains revealed that HMRF, BayesSpace, and SpaGCN failed to segregate the cortical layers L2/3 and L5 despite the well-known functional and morphological distinction between these two layers [38]. In addition, the four cortical layers detected by BASS are well segregated from each other with smooth boundaries between them, while the layers detected by BayesSpace and SpaGCN are somewhat intermingled together without smooth tissue boundaries separating them (Fig. 4D). Importantly, the ability of BASS for multi-sample integrative analysis further enhanced the spatial domain detection accuracy on the same tissue section used for the one sample analysis (ARI = 0.85; Fig. 4E). In particular, the boundary between the layers L2/3 and L5 was even more precisely captured in the multi-sample integrative analysis and more closely resembles the expected truth as compared to the analysis using only one tissue section. In addition, similar to what we have found in the sample of focus, BASS accurately captured the four spatial domains in the other two sections, more so than the other methods, in both the single-section and multi-section analyses (Fig. 4E and Additional file 1: Fig. S23). The results confirmed the superior performance of BASS for spatial domain detection and multi-sample integrative analysis.

**Fig. 5:**
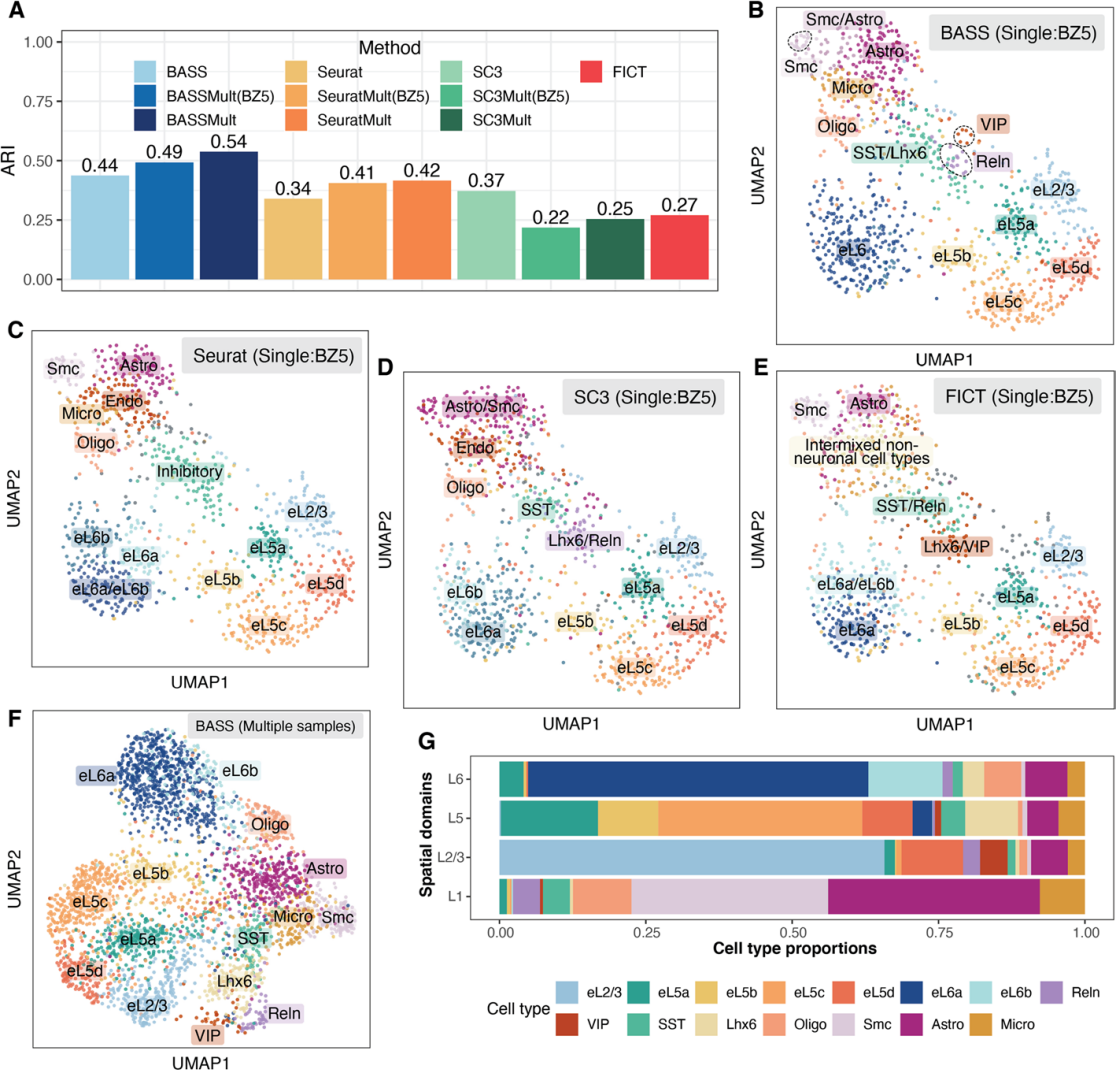
Clustering cell types in the STARmap dataset. **A** Barplots of ARI display the accuracy of different methods for cell type clustering. Compared methods include BASS, Seurat, SC3, and FICT. Results for BASS, Seurat, and SC3 are shown for both the single tissue section analysis on the tissue section BZ5 and multi-sample analysis (BASSMult, SC3Mult, and SeuratMult) that fitted all the three tissue sections (BZ5, BZ9, and BZ14) and evaluated ARI on the section BZ5 (e.g., BASSMult (BZ5)) as well as on all three tissue sections (e.g., BASSMult). **B–E** UMAP visualization of cell type clustering results on BZ5 by **B** BASS, **C** Seurat, **D** SC3, and **E** FICT for analyzing the single section BZ5. **F** UMAP visualization of cell type clustering results on three sections (BZ5, BZ9, and BZ14) by the multi-sample version of BASS. **G** Cell type compositions estimated by the multi-sample version of BASS. Excitatory neurons: eL2/3, eL5a, eL5b, eL5c, eL5d, eL6a, and eL6b; Inhibitory neurons: Reln, VIP, SST, and Lhx6; Oligo: oligodendrocytes; Smc: smooth muscle cells; Astro: astrocytes; and Micro: microglia

Next, we examined the results from different methods on cell type clustering. We first evaluated the performance of different methods for cell type clustering on a single tissue section. Consistent with the simulations, BASS achieved accurate cell type clustering (ARI = 0.44), more so than Seurat (ARI = 0.34), SC3 (ARI = 0.37), and FICT (ARI = 0.27) (Fig. 5A). Specifically, BASS detected major cell types that included six excitatory neuronal subtypes, three inhibitory neuronal subtypes, and four non-neuronal subtypes with distinct marker gene expression (Fig. 5B and Additional file 1: Fig. S24A). In contrast, Seurat erroneously produced a cluster that was a mixture of two other cell types (eL6a/eL6b) as evident by their marker gene expression, failed to delineate inhibitory neuronal subtypes (SST, VIP, Lhx6, and Reln), and produced one NA cluster with no clear marker gene expression (Fig. 5C and Additional file 1: Fig. S24B). The resulting NA cluster was likely because cells of different cell types were somehow split from their main clusters and then combined together. SC3 erroneously clustered smooth muscle cells, astrocytes, and VIP inhibitory neurons into the same cluster, despite their vastly distinct expression profiles (Fig. 5D and Additional file 1: Fig. S24C). In addition, SC3 erroneously produced one cluster (eL6a-2) that had similar marker gene expression to eL6a-1 and produced two NA clusters with no marker gene expression (Additional file 1: Fig. S24C). FICT failed to delineate inhibitory neuronal subtype SST from Reln, failed to delineate inhibitory neuronal subtype Lhx6 from VIP, and intermixed different non-neuronal subtypes (Fig. 5E and Additional file 1: Fig. S24D). Next, we examined the cell type clustering results from the multi-sample version of BASS by analyzing three tissue sections together (Fig. 5F and Additional file 1: Fig. S25). Consistent with the simulations, the integrative analysis provided more accurate cell type clustering results (ARI = 0.49; Fig. 5A, F) than analyzing a single tissue sample, highlighting the benefits of multi-sample analysis. In particular, the multi-sample integrative analysis further delineated the excitatory neurons at layer 6 (eL6) into two neuronal subtypes (eL6a and eL6b) and segregated the SST inhibitory neurons from the Lhx6 inhibitory neurons (Fig. 5F and Additional file 1: Fig. S25A). These cell type clustering results from multi-sample analysis were supported by the expression pattern of marker genes (Additional file 1: Fig. S25B). For example, the two cell types (eL6a and eL6b) that were clustered together (eL6∗) in the single tissue section analysis, but segregated in the multi-sample integrative analysis had a distinct expression of marker genes that corresponded to the two excitatory neuronal subtypes (eL6a: *Syt6*; eL6b: *Ctgf*). Finally, the multi-sample analysis of BASS also outperformed the two other cell type clustering methods (Seurat and SC3) that can be adapted to make use of multiple tissue sections in an ad hoc fashion (Fig. 5A and Additional file 1: Fig. S25C-G).

Finally, we examined the spatial distribution of cell types identified by BASS on the tissue and assessed the cell type compositions in each spatial domain. Overall, we found that excitatory neurons were often enriched in spatially restricted domains while inhibitory neurons and glial cells often dispersed across multiple cortical layers (Additional file 1: Fig. S26). Specifically, six out of the seven excitatory neuronal subtypes were highly enriched in a single cortex layer, defining the laminar architecture of the cortex: one excitatory subtype (eL2/3) was enriched in L2/3; three (eL5a, eL5b, eL5c) were enriched in L5; two excitatory subtypes (eL6a, eL6b) were enriched in L6; and the remaining excitatory subtype (eL5d), labeled by immediate-early genes *Fos*, *Egr2*, and *Egr4*, was enriched in both L2/3 and L5. The four inhibitory neurons, on the other hand, were dispersed across multiple cortical layers. For example, the inhibitory neuronal subtype (SST) marked by *Sst* was present across all four layers while the inhibitory neuronal subtype (Lhx6) marked by *Lhx6* was primarily located at both L5 and L6. For non-neuronal cells, oligodendrocytes were mainly located at L6; smooth muscle cells were mainly located at L1 whereas astrocytes and microglial cells were dispersed across the entire tissue. Consistent with the distinct distributional pattern of multiple cell types on the tissue, each cortical layer was also composed of a distinct mixture of cell types (Fig. 5G). Specifically, L1 was mainly composed of non-neuronal cells (astrocytes: 35%; smooth muscle cells: 31%; oligodendrocytes: 13%; and microglial cells: 9%), with astrocytes and smooth muscle cells playing key roles in regulating blood-brain barrier function and cerebral blood flow [39, 40]. The remaining three cortical layers consisted of mainly excitatory neuronal subtypes, with eL2/3 comprising 66% of cells in L2/3; eL5a, eL5b, and eL5c comprising 62% of cells in L5; and eL6a and eL6b comprising 71% of cells in L6.

### Mouse hypothalamus data by MERFISH

**Fig. 6:**
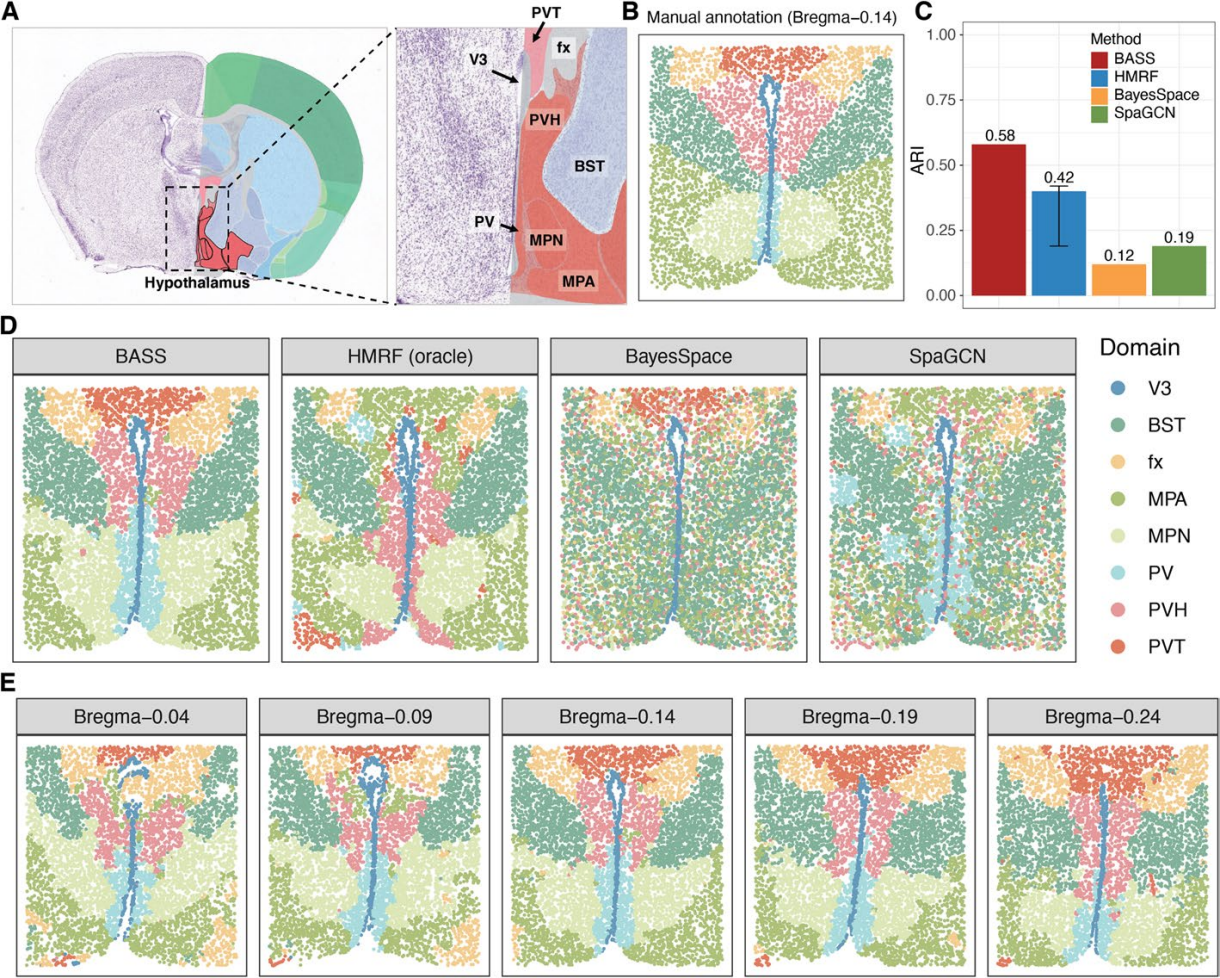
Detecting spatial domains in the MERFISH dataset. **A** An anatomic reference atlas obtained from the Allen Mouse Brain Atlas displays the spatial domains of the mouse hypothalamus region. **B** Annotated spatial domain labels for the tissue section Bregma-0.14 based on spatial gene expression patterns and the Allen Mouse Brain Atlas. **C** Barplots of ARI show the accuracy of different methods for spatial domain detection on the tissue section Bregma-0.14. The compared methods include BASS, HMRF, BayesSpace, and SpaGCN. For HMRF, the range of ARI based on a pre-defined list of *β*s is shown with an error bar. **D** The identified spatial domains on the tissue section Bregma-0.14 are shown for BASS, HMRF, BayesSpace, and SpaGCN. **E** The identified spatial domains on five tissue sections (Bregma-0.04, Bregma-0.09, Bregma-0.14, Bregma-0.19, and Bregma-0.24) were obtained with the multi-sample analysis of BASS. V3: the third ventricle; BST: bed nuclei of the strata terminalis; fx: columns of the fornix; MPA: medial preoptic area; MPN: medial preoptic nucleus; PV: periventricular hypothalamic nucleus; PVH: paraventricular hypothalamic nucleus; and PVT: paraventricular nucleus of the thalamus

The second dataset we examined is from the MERFISH technology, consisting of five adjacent tissue sections obtained from the preoptic region of the mouse hypothalamus, which is an important region in the center of the brain that comprises multiple nuclei and controls many social behaviors such as reproduction and circadian rhythms as well as homeostatic functions such as neuroendocrine and cardiovascular regulation [41]. The five sections include Bregma-0.04 (5488 cells), Bregma-0.09 (5557cells), Bregma-0.14 (5926 cells), Bregma-0.19 (5803 cells), and Bregma-0.24 (5543 cells), with expression measurements collected on a common set of 155 genes. The cells on all tissue sections were carefully annotated to eight distinct structures that included the third ventricle (V3), bed nuclei of the strata terminalis (BST), columns of the fornix (fx), medial preoptic area (MPA), medial preoptic nucleus (MPN), periventricular hypothalamic nucleus (PV), paraventricular hypothalamic nucleus (PVH), and paraventricular nucleus of the thalamus (PVT) (Fig. 6A,B and Additional file 1: Fig. S27). We performed single-section analysis on the tissue section Bregma-0.14 and multi-section analysis on all five tissue sections.

We first examined the results from different methods on spatial domain detection. In the analysis, BASS detected major spatial domains that highly resemble the underlying histological annotations (ARI = 0.58; Fig. 6C,D). In contrast, the spatial domains detected by HMRF (ARI = 0.42), BayesSpace (ARI = 0.12), and SpaGCN (ARI = 0.19) do not generally match the underlying truth (Fig. 6C,D). For example, HMRF failed to segregate the three key regions of hypothalamus (PVT, MPA, and PVH) from each other. SpaGCN failed to detect the MPN, MPA, and PVH regions while BayesSpace barely detected any segments of the tissue with a smooth boundary except for the V3 and PVT regions. In particular, none of the other three domain detection methods could identify and segregate the PV region and the MPN region, despite their important structural and functional roles in the nervous system. There, PV consists of a thin sheet of small neurons located in the wall of the third ventricle and these neurons regulate the release of gonadotropin-releasing hormone (GnRH) and growth hormone (GH) [42, 43], whereas MPN occupies a more lateral location than PV and plays an important role in reproductive and parental behaviors [44]. Importantly, multi-sample integrative analysis of five adjacent spatial transcriptomic tissue sections with BASS provided further insights into the structural organization of the preoptic region of hypothalamus that could not otherwise be achieved by the single-sample analysis of the other methods (Fig. 6E and Additional file 1: Fig. S27). In particular, the eight main spatial domains were present in all five tissue sections, with their shape and size varying across sections. For example, from anterior to posterior, the PVT and BST regions increased their sizes while the PV and MPN regions reduced their sizes.

**Fig. 7:**
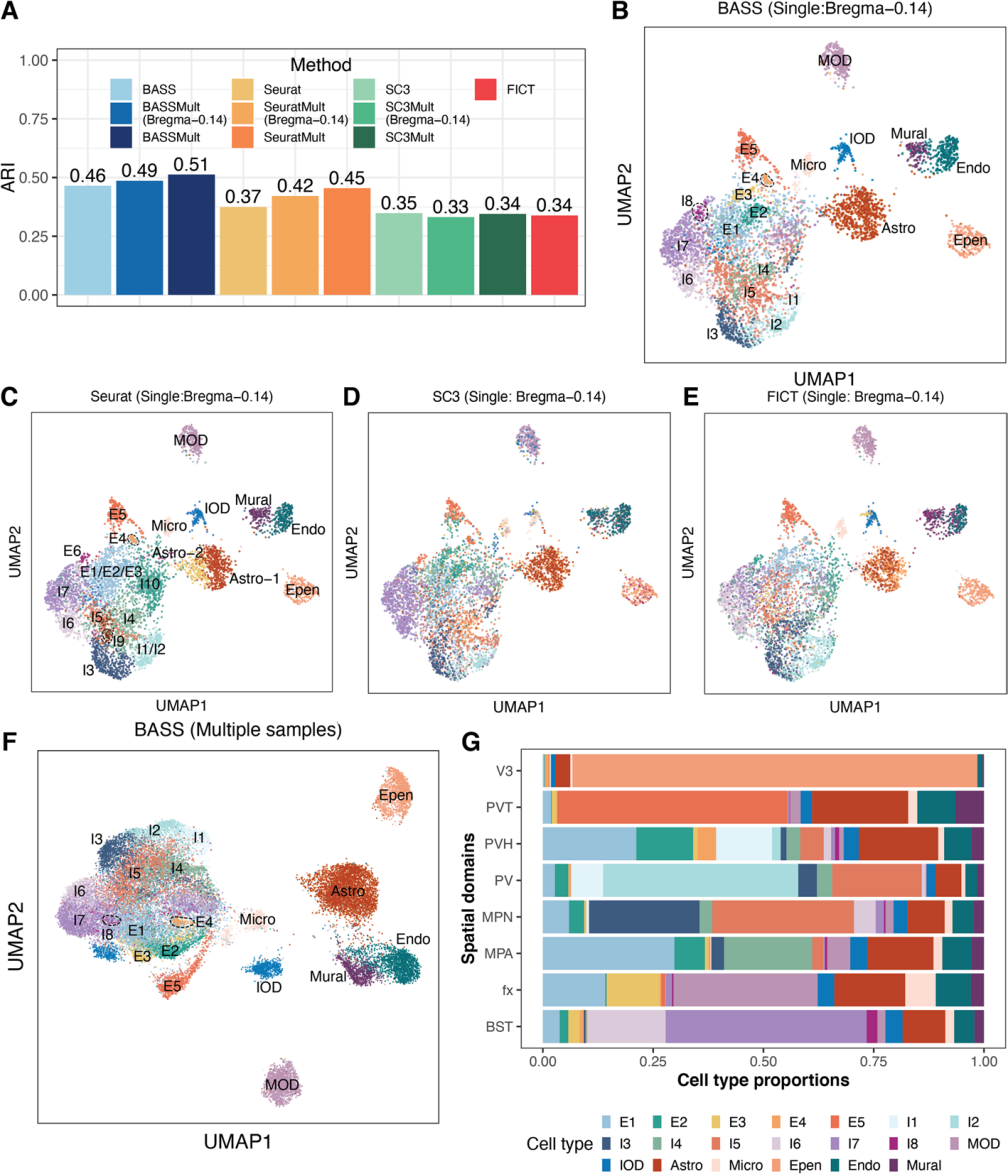
Clustering cell types in the MERFISH dataset. **A** Barplots of ARI display the accuracy of different methods for cell type clustering. Compared methods include BASS, Seurat, SC3, and FICT. Results for BASS, Seurat, and SC3 are shown for both the single tissue section analysis on the tissue section Bregma-0.14 and multi-sample analysis (BASSMult, SC3Mult, and SeuratMult) that fitted all five tissue sections (Bregma-0.04, -0.09, -0.14, -0.19, and -0.24) and evaluated ARI on the section Bregma-0.14 (e.g., BASSMult (Bregma-0.14)) as well as on all five tissue sections (e.g., BASSMult). **B–E** UMAP visualization of cell type clustering results on Bregma-0.14 by **B** BASS, **C** Seurat, **D** SC3, and **E** FICT for analyzing the single section Bregma-0.14. **F** UMAP visualization of cell type clustering results on five sections by the multi-sample version of BASS. **G** Cell type compositions estimated by the multi-sample version of BASS. Excitatory neurons: E1, E2, E3, E4, and E5; Inhibitory neurons: I1, I2, I3, I4, I5, I6, I7, and I8; MOD: mature oligodendrocytes; IOD: immature oligodendrocytes; Astro: astrocytes; Micro: microglial cells; Epen: ependymal cells; Endo: endothelial cells; and Mural: mural cells

Next, we examined the results from different methods on cell type clustering. We first evaluated the performance of different methods for cell type clustering on a single tissue section. Consistent with the simulations, BASS achieved accurate cell type clustering (ARI = 0.46), more so than Seurat (ARI = 0.37), SC3 (ARI = 0.35), and FICT (ARI = 0.34) (Fig. 7A). Specifically, BASS detected major cell types that included five excitatory neuronal subtypes, eight inhibitory neuronal subtypes, and seven non-neuronal subtypes with distinct marker gene expression (Fig. 7B and Additional file 1: Fig. S28A). In contrast, Seurat failed to delineate excitatory neuronal subtypes E1, E2, and E3 and failed to delineate inhibitory neuronal subtypes I1 and I2, despite the distinct expression profiles of each subtype (Fig. 7C and Additional file 1: Fig. S28B). However, we also noticed that Seurat has split the astrocytes into two clusters (Astro-1 and Astro-2; Fig. 7C) with very similar overall gene expression pattern (Additional file 1: Fig. S28B). We performed a careful differential expression analysis and identified a few genes that displayed very subtle expression differences between the two clusters (Additional file 1: Fig. S29). Therefore, it is possible that the two clusters may represent two astrocyte subtypes with very similar expression patterns. Consistent with the simulations, the performance of SC3 was relatively poor, presumably due to the large number of cells in the data. Specifically, SC3 assigned microglial cells and immature oligodendrocytes into the same cluster; assigned mural cells and endothelial cells into the same cluster; and failed to segregate certain neuronal subtypes (e.g., I6 and I7) (Fig. 7D and Additional file 1: Fig. S28C). FICT erroneously separated astrocytes into three clusters, separated immature oligodendrocytes into two clusters, and produced a cluster that is a mix of mural cells and endothelial cells (Fig. 7E and Additional file 1: Fig. S28D). We applied BASS, SC3, and Seurat for multi-sample analysis (Fig. 7F and Additional file 1: Fig. S30). We found that multi-sample analysis with BASS yielded similar cell type clustering accuracy as the single-section analysis for Bregma-0.14 (ARI = 0.49; Fig. 7B vs Additional file 1: Fig. S30B), presumably because the number of cells in the single tissue section was already very large. Multi-sample analysis with Seurat improved upon the single-section analysis (ARI = 0.42; Fig. 7A and Additional file 1: Fig. S30C-D) while multi-sample analysis with SC3 yielded lower cell type clustering performance as compared to the single-section analysis, likely due to its potential reduction in performance with the increased cell number, as explained earlier (ARI = 0.33; Fig. 7A and Additional file 1: Fig. S30E-F). The comparison for multi-section vs single-section analysis for the three methods was consistent with the simulations and highlighted the benefits of BASS.

Then, we estimated the cell type composition in each spatial domain and examined the spatial distribution of cell types identified by BASS across tissue locations. We found that the majority of excitatory and inhibitory neurons were enriched in certain tissue regions whereas the majority of glial cells and other cell types were often dispersed throughout the tissue (Additional file 1: Fig. S31). Specifically, the excitatory neurons (E1, E2, E3, E4, and E5) were highly enriched in the PVT, fx, PVH, and MPA regions, with a minor dispersion pattern visualizable across many other regions. Inhibitory neurons (I1, I2, I3, I4, I5, I6, I7, and I8) on the other hand were highly enriched in the BST, MPA, MPN, PV, and PVH regions, with a minor dispersion pattern visualizable across many other regions. The spatial enrichment of cell types in the hypothalamus underlies their structural and functional roles. For example, the inhibitory neurons in BST project inhibitory fibers to the lateral hypothalamus and are important for feeding regulation [45]. For non-neuronal cells, mature oligodendrocytes were highly enriched in the fx region, which is part of the limbic system and consists of a bundle of nerve fibers that carry signals from the hippocampus to other parts of the brain. Continuous oligodendrogenesis in the fx region [46] is important for myelination and ensures rapid conduction of neural signals [47]. The ependymal cells formed the epithelial lining of V3 and separated the cerebrospinal fluid in the ventricle from the brain. In contrast, the other glial cells (immature oligodendrocytes, astrocytes, microglial cells, endothelial cells, and mural cells) were all dispersed across the entire tissue. Consistent with the distinct distributional pattern of multiple cell types on the tissue, we found that all spatial domains in the hypothalamus were composed of a distinct mixture of cell types (Fig. 7G). For example, the four regions (V3, BST, PV, and PVT) were each dominated by one cell type (V3: ependymal cells; BST: I7; PV: I2; and PVT: E5) and the dominant cell type comprised nearly 50% or more cells in the corresponding region. In other regions such as fx, MPA, MPN, and PVH, we found a mixture of cell types with comparable proportions. For example, the fx was mainly composed of mature oligodendrocytes, astrocytes, E1, and E3, with proportion estimates being 0.33, 0.16, 0.14, and 0.12 respectively.

Finally, the accurate detection of tissue domains by BASS also allows us to perform additional downstream analysis to further characterize the transcriptomic architecture underlying complex tissues. We conducted a differential expression analysis to identify both cell type marker genes and domain marker genes based on the cell type or spatial domain estimates from BASS. In the analysis, we found that each cell type/spatial domain exhibit a unique expression signature, allowing us to identify important gene markers for each cell type and each spatial domain (Additional file 1: Figs. S33-S34). In particular, we identified top marker genes *Slc18a2* and *Scg2* for the PV domain and *Calcr* and *Nts* for the MPN domain, which are important gene candidates for future studies. Specifically, *Slc18a2* (Solute Carrier Family 18 Member A2) gene encodes a transmembrane protein that facilitates the uptake of monoamine neurotransmitters into synaptic vesicles and plays an essential role in dopamine regulation [48]. The inhibitory effects of dopamine on gonadotrophin secretion have been extensively documented [49–52]. Indeed, genetic variants in *Slc18a2* in human have been identified to be associated with the polycystic ovary syndrome (PCOS), a hormone disorder related to ovaries [53]. The important role of *Slc18a2* in gonadotrophin secretion may underlie the well-known function of PV domain on regulating the release of gonadotropin-releasing hormone [42]. As a second example, *Calcr* (Calcitonin Receptor) encodes a high affinity receptor for the peptide hormone calcitonin. In mice, the amylin-calcitonin receptor signaling has been reported to mediate affiliative social contacts [54]. Therefore, the identification of *Calcr* by BASS may provide the transcriptomic mechanism underlying the regulatory function of the MPN region in reproductive and parental behavior [44].

### DLPFC 10x Visium data with non-single-cell resolution

While we have primarily focused on analyzing single-cell resolution spatial transcriptomics, we note that BASS can also be applied to analyze non-single-cell resolution spatial transcriptomics. Specifically, we can treat each spatial location in non-single-cell resolution spatial transcriptomics as a “pseudo cell” and directly apply BASS for data analysis. Certainly, the cell type assignments for the pseudo cells by BASS no longer have the cell type interpretation, and consequently, BASS can only be used for spatial domain detection and multi-sample analysis in non-single-cell resolution spatial transcriptomics. To illustrate the benefits of BASS in this context, we applied BASS to analyze the DLPFC data from the 10x Visium platform, which consists of 12 tissue sections obtained from the human dorsolateral prefrontal cortex of three adult donors (data details in the “Methods” section). The spots on each tissue section were carefully annotated by the original study [32] into seven laminar clusters that included six neural layers from L1 to L6 and the white matter (WM). Because DLPFC is not of single-cell resolution, we focused our analysis only on spatial domain detection but not cell type clustering. We performed single-section analysis on each of the 12 tissue sections and multiple-section analysis on the four tissue sections from each donor.

**Fig. 8:**
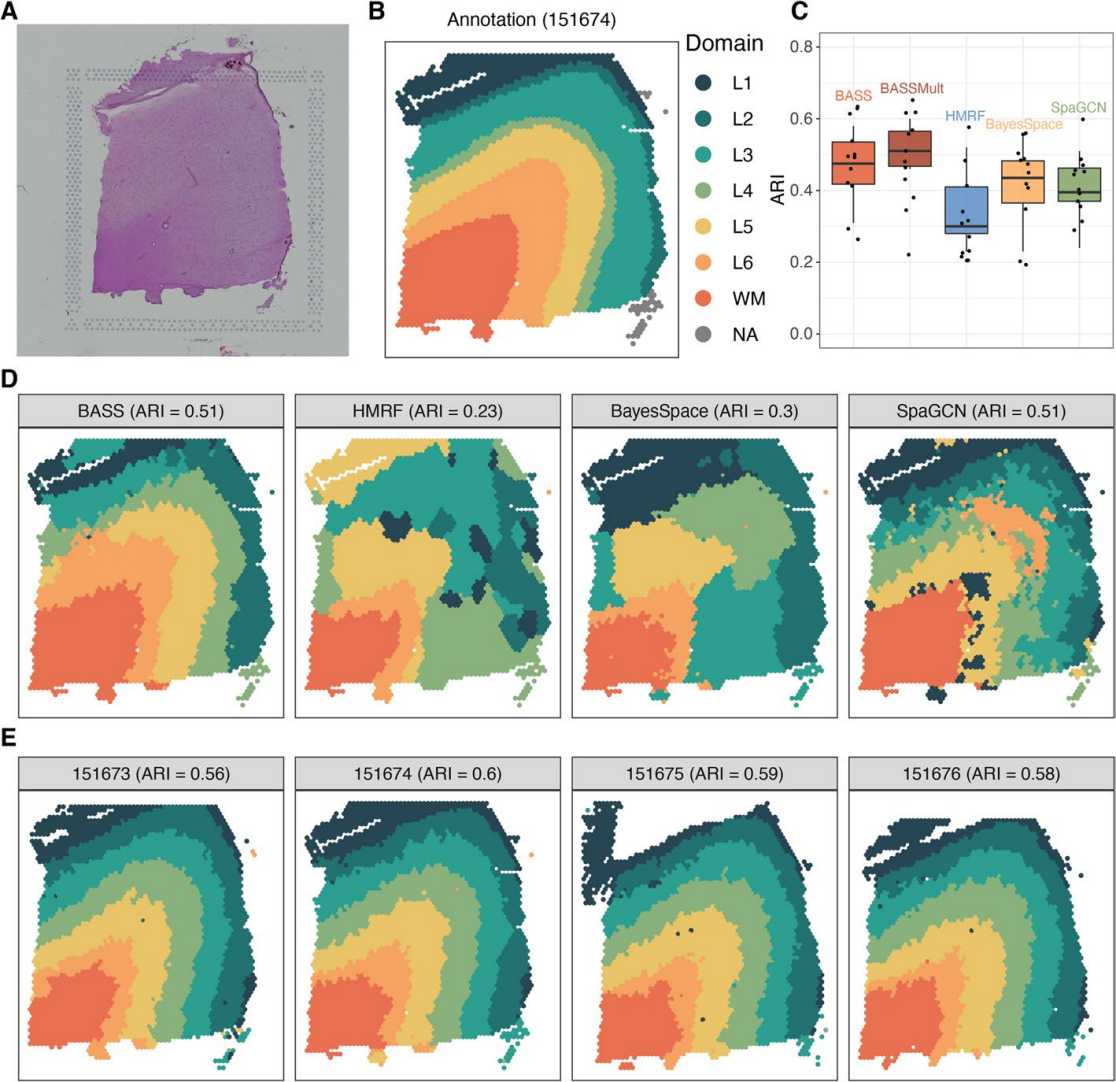
Detecting spatial domains in the DLPFC 10x Visium dataset. **A** A hematoxylin and eosin (H&E) staining image for the tissue section 151674. **B** Manually annotated spatial domain labels for the tissue section 151674. **C** Boxplots of ARI show the accuracy of different methods for spatial domain detection across 12 tissue sections. The compared methods include BASS that performs single tissue section analysis, BASSMult that performs multi-sample analysis that jointly fitted four tissue sections from each adult donor, the oracle version of HMRF, BayesSpace, and SpaGCN. **D** The identified spatial domains on the tissue section 151674 are shown for BASS, HMRF, BayesSpace, and SpaGCN. **E** The identified spatial domains on four tissue sections (151673, 151674, 151675, and 151676) were obtained with BASSMult

In the single-section analysis, BASS detected major spatial domains that highly resemble the underlying histological annotations for most tissue sections (median ARI across sections = 0.48), more so than HMRF (median ARI = 0.30), BayesSpace (median ARI = 0.44), and SpaGCN (median ARI = 0.40; Fig. 8B and Additional file 1: Fig. S35). Importantly, the integrative analysis of four tissue sections from each adult donor further improved the spatial domain detection (median ARI = 0.51; Fig. 8B) and produced consistent spatial domains across the four sections (Additional file 1: Fig. S35). For example, on tissue section 151674, the single-section analysis of BASS (ARI = 0.51), HMRF (ARI = 0.23), BayesSpace (ARI = 0.30), and SpaGCN (ARI = 0.51) all failed to detect the consecutive cortical layers (Fig. 8C), presumably due to the poor quality of this tissue section. However, the multi-sample integrative analysis with BASS greatly improved the detection of spatial domains in this particular sample and produced cortical layers that highly resemble the underlying annotations and are consistent across all four sections (ARI = 0.60; Fig. 8D). Similar observations can also be made in the other two sets of tissue sections (151507-151510 and 151669-151672; Additional file 1: Fig. S35), confirming the performance of BASS on spatial domain detection and multi-sample integrative analysis in non-single-cell resolution spatial transcriptomics.

### Computational performance

In terms of both running time and memory usage, BASS is comparable to the other spatial domain detection methods and cell type clustering methods (Additional file 1: Table S1). For a typical 10x Visium data with about 5000 spots, BASS takes about 8 min and uses 2 GB of memory. In addition, both the running time and memory usage of BASS scale linearly with the sample size of the data, which makes BASS scalable to analyzing tens to hundreds of thousands of cells/spots.

## Discussion

Although BASS, HMRF, and BayesSpace all employ a Potts model, BASS has introduced additional hierarchical modeling structures on top of the Potts model to allow for flexible and effective spatial transcriptomic modeling. The additional hierarchical modeling structures allow BASS to make fundamentally different and more effective assumptions on the composition of spatial domains as compared to HMRF and BayesSpace. Specifically, both HMRF and BayesSpace define the spatial domain to be a region with a homogenous gene expression and model the gene expression of all cells in a spatial domain with the same distribution, either a multivariate normal distribution for HMRF or a multivariate *t*-distribution for BayesSpace. However, spatial domains are often composed of multiple cell types characterized by distinct gene expression profiles. Modelling gene expression in a spatial domain with the same distribution can be insufficient for capturing the gene expression heterogeneity across cell types. Therefore, BASS has introduced the cell type composition as an intermediate layer in the hierarchical modelling structure to explicitly model distinct gene expression of different cell types. Consequently, BASS is able to define a spatial domain to be a region with a unique cell type composition to better capture the gene expression heterogeneity within each spatial domain and achieve improved performance. In addition, BASS infers the spatial interaction parameter *β* in the Potts model based on the data at hand while HMRF and BayesSpace fix the parameter to a user-specified value. The inference of *β* in BASS also contributes to its improved performance. Finally, from the model inference perspective, although both BASS and BayesSpace are based on a Bayesian framework, BASS uses the Swendsen-Wang algorithm to sample the spatial domain labels while BayesSpace uses a Gibbs sampling algorithm. The Swendsen-Wang algorithm is known to have a much better mixing rate than the Gibbs sampling algorithm, thus also helping BASS to achieve an improved accuracy [55].

The number of cell types and spatial domains are two important hyper-parameters that need to be specified in BASS. Given our investigation in the simulation (Additional file 1: Figs. S16-S18), here we provide a practical guideline for selecting the optimal number of these two hyper-parameters for BASS, when the underlying truths are unknown. Our recommendation is to start with a relatively large number of cell types and a relatively large number of spatial domains. We recommend to first select the optimal number of spatial domains, given that spatial domains can be robustly detected by BASS with mis-specified cell type number. To do so, we calculate the number of cells in each spatial domain and set the number of spatial domains to be the largest one that still leads to a relatively large number of cells in each domain determined by the user (e.g., with > 3% of cells per domain). After selecting the optimal number of spatial domains, we proceed to select the optimal number of cell types. Here, care needs to be taken when selecting the optimal number of cell types because the small cell type clusters with a relatively small number of cells can either be redundant clusters or represent rare cell types. Therefore, we recommend examining each small cell type cluster carefully, by, for example, performing a differential expression analysis to examine cell type cluster marker genes. Careful examination will allow us to determine whether the small cell type clusters are rare cell types or redundant clusters, thus informing the choice of the optimal number of cell types.

BASS can also be applied to other spatial transcriptomic technologies after necessary pre-processing steps. For example, some recent spatial transcriptomic technologies, such as HDST [8] and Seq-Scope [9], are of subcellular resolution, with each measured spot capturing transcripts from part of a cell. For these technologies, we can in principle first aggregate the expression levels measured on the multiple spots of the same cell into units of cells and then apply BASS on the aggregated gene expression measurements. However, accurate characterization of cell boundaries and matching spots to cells for these technologies remain as computational challenging tasks [9]. Existing approaches either performs data binning [8, 9], which aggregates expression measurements of neighboring spots into grids with size of single cells (10 μm-sided) or takes advantage of histology images to identify cell boundaries (e.g., watershed algorithm) and then matches spots to each cell [9]. However, neither approach is ideal. For data binning, aggregated spots of each grid do not necessarily belong to the same cell. For the histology-image-based method, it is challenging to segment cells with small sizes [9]. To address these challenges, new computational tools may be needed to facilitate the integration of both histology images and spatial expression data to improve the cell boundary detection.

There are several important future extensions for BASS. For example, while we have primarily focused on using gene expression data as input, we note that the BASS modeling framework is flexible and can be easily used to incorporate information from the histology images in the form of additional feature input to further enhance its performance. As another example, we have used the low-dimensional components from the principal component analysis (PCA) on the normalized gene expression matrix as input for BASS. We note that BASS is not restricted to PCA and can be paired with other dimension reduction methods with their low-dimensional components as input. For example, SPICEMIX [56] and SpatialPCA [57] are dimension reduction methods recently developed for spatial transcriptomics and have shown promising results for either cell type inference or spatial domain detection. Using the latent factors from SPICEMIX or SpatialPCA as input for BASS could potentially enhance its performance. Finally, BASS can be modified to take advantage of its normal-gamma prior on the mean parameters of the gene expression for informative gene selection. Specifically, the feature-specific scaling factor (*λ*_*j*_) in BASS reflects how informative the *j*th feature is in distinguishing different cell types, with a larger *λ*_*j*_ indicating a more informative expression feature. Therefore, when the gene expression matrix is used as input for BASS, we can identify cluster informative genes by inspecting the parameter *λ*_*j*_. We followed this idea and applied BASS to the BZ5 sample in the STARmap data set. Indeed, genes with large *λ*_*j*_ estimates are marker genes of different cell types (Additional file 1: Fig. S32). For example, *Bgn* and *Mgp*, which had the highest *λ*_*j*_ estimates, are marker genes of the smooth muscle cells; *Sst*, *Pnoc*, *Npy*, and *Vip* are marker genes of different inhibitory neurons; *Itgam* is a marker gene of the microglial cells; and *Pcp4* is a marker gene of the excitatory neurons in layer 6 (eL6a and eL6b) (Additional file 1: Fig. S32B). However, applying BASS directly on the gene expression matrix instead of the low-dimensional space can be computationally intense and may also suffer from the noise contained in the expression matrix. Therefore, further thoughts and methodological development in the future is likely necessary to make this strategy practical.

The accurate cell types and spatial domains detected by BASS can be paired with many other analytic tools to further improve various downstream applications to reveal additional biological insights. For example, SpatialCorr [58] is a recent method that aims to identify sets of genes with spatially varying correlation structures within or between pre-determined spatial domains. The spatial domains detected by BASS can be paired with SpatialCorr to detect genes with coordinated regulation within or between tissue regions. Knowing such coordinated regulation can help identify novel cell subpopulations and provide important insights into the gene regulatory network within the subpopulation [58]. As another example, trajectory analysis can be carried out on the cell types or the spatial domains inferred by BASS to facilitate the investigation into cancer progression, cell type differentiation, and structural feature development [57, 59]. In addition, because spatial transcriptomic studies often collect data from multiple adjacent sections, tools such as PASTE [60] and GPSA [61] for aligning adjacent tissue sections can be paired with BASS for 3D tissue construction [60] or 2D construction of a larger tissue area [9]. There, the accurately detected cell types and spatial domains from BASS allow us to explore the spatial domain structures and spatial distribution patterns of cell types in 3D or a larger 2D tissue area.

## Conclusions

In conclusion, we have presented BASS for multi-scale and multi-sample analysis in single-cell resolution spatial transcriptomics and for spatial domain detection and multi-sample analysis in non-single-cell resolution spatial transcriptomics. In comparison to existing approaches, BASS produces both accurate cell type and spatial domain assignments, and allows for an integrative analysis of spatial transcriptomics across multiple tissue sections. We have illustrated the benefits of BASS through both simulations and in-depth analyses of three spatial transcriptomic datasets.

## Methods

### BASS: model and inference

Our method applies to a wide variety of spatial transcriptomic data types obtained by high-resolution spatial transcriptomic technologies which can measure gene expression at the single-cell level or approximately the single-cell level. These technologies include in situ sequencing (ISS)-based ones such as STARmap [10] and FISSEQ [11]; single molecular fluorescent in situ hybridization-based (smFISH) ones such as MERFISH [12], seqFISH [13], seqFISH+ [14], and osmFISH [15], as well as the upcoming 10x Visium HD. For these data types, we perform transcriptomic analyses in the tissue at two different anatomical scales. Specifically, at the single-cell scale, we perform clustering analysis to both identify cell types and assign cell type labels to each individual cell. At the tissue domain scale, we segment the tissue into spatial domains in a de novo fashion and characterize the cell type composition in each detected domain. We carry out the two analyses at different scales jointly in a coherent fashion based on a Bayesian hierarchical model, which allows us to seamlessly integrate gene expression information with spatial information to improve the effectiveness of both analyses. Importantly, our method allows for multi-sample integrative analysis of spatial transcriptomic data measured on multiple tissue sections in the same anatomic region, which allows us to borrow critical biological information across tissue sections to further enhance the analytic performance.

We describe our method in this section, with its inference details provided in the Additional file 1: Supplementary notes. To set up notations, we assume that the spatial transcriptomic study measures gene expression for a common set of *P* genes on *L* different tissue sections. We denote the number of cells measured on the *l*th tissue section as *N*_*l*_, with *l* ∈ {1, …, *L*}. We assume that the cells across all tissue sections belong to *C* different cell types, and we denote ${c}_i^{(l)}$ as the cell type label for the *i*th cell on tissue section *l*, with ${c}_i^{(l)}\in \left\{1,\dots, C\right\}$. To simplify the algebra, we combine the cell type labels across all cells on section *l* into an *N*_*l*_-vector of ${\boldsymbol{c}}^{(l)}={\left({c}_1^{(l)},\cdots, {c}_{N_l}^{(l)}\right)}^T$. In addition, we assume that the tissue consists of *R* different spatial domains, each characterized by a distinct cell type composition. We denote ${z}_i^{(l)}$ as the spatial domain label for the *i*th cell on tissue section *l*, with ${z}_i^{(l)}\in \left\{1,\dots, R\right\}$. We also combine the spatial domain labels across all cells on section *l* into an *N*_*l*_-vector of ${\boldsymbol{z}}^{(l)}={\left({z}_1^{(l)},\cdots, {z}_{N_l}^{(l)}\right)}^T$. We further combine cell type and spatial domain labels across all sections into vectors of ***c*** = (***c***^(1)*T*^, …, ***c***^(*L*)*T*^)^*T*^ and ***z*** = (***z***^(1)*T*^, …, ***z***^(*L*)*T*^)^*T*^, with the vector size for both being ${\sum}_{l=1}^L{N}_l$. We denote ***π***_*r*_ ***=*** (*π*_1*r*_, …, *π*_*Cr*_)^*T*^ as the *C-*vector of the cell type composition in the *r*th spatial domain, where *π*_*cr*_ represents the proportion of cell type *c* in the spatial domain *r*, with ${\sum}_{c=1}^C{\pi}_{cr}=1$. We treat the cell type label ${c}_i^{(l)}$, spatial domain label ${z}_i^{(l)}$, and cell type composition in each spatial domain *π*_*cr*_ as unknown and aim to infer them using both the gene expression measurements and spatial location information obtained from spatial transcriptomics.

For the gene expression measurements, we combine cells across all *L* tissue sections, conduct library size normalization [62, 63] followed by a log2-transformation (after adding a pseudo-count of 1), and perform dimension reduction on the normalized expression matrix to extract *J* low-dimensional expression features. We denote ***X***^(*l*)^ as the resulting *N*_*l*_ × *J* low-dimensional expression feature matrix for section *l*, where ${\boldsymbol{x}}_i^{(l)}$ is the *J*-vector of expression features for the *i*th cell there, with *i* ∈ {1, …, *N*_*l*_}. Dimension reduction removes noise from the original expression matrix and preserves and compresses the gene expression information into a low-dimensional manifold [64]. Dimension reduction can be performed with any standard methods, and we simply use the principal component analysis following the recommendation of [64]. In both the simulation study and real data applications, we extract the top 20 principal components (PCs) as expression features. With the extracted low-dimensional expression features, we perform data alignment and batch effect adjustment to align expression data from different tissue sections using Harmony [65] per recommendation of [66]. For the spatial location information, we construct a neighborhood graph *V*^(*l*)^ among cells on each tissue section *l* by identifying for each cell its *k* nearest neighbors. With both expression and location information, we consider the following three equations to model the relationship among gene expression features, cell type labels, spatial domain labels, cell type compositions, and neighborhood graphs in a hierarchical fashion:

1 $${\boldsymbol{x}}_i^{(l)}\mid {c}_i^{(l)}=c\sim Normal\left({\boldsymbol{\mu}}_c,\boldsymbol{\Sigma} \right),$$

2 $${c}_i^{(l)}\mid {z}_i^{(l)}=r\sim Cat\left({\boldsymbol{\pi}}_{\boldsymbol{r}}\right),$$

3 $${\boldsymbol{z}}^{(l)}\sim Potts\left({V}^{(l)},\beta \right).$$

Above, the first equation models the expression feature of the *i*th cell on section *l*,${\boldsymbol{x}}_i^{(l)}$, as depending on its cell type label ${c}_i^{(l)}$. In particular, conditional on the *i*th cell belonging to the cell type *c*, ${\boldsymbol{x}}_i^{(l)}$ follows a normal distribution with a *c*-cell-type-specific mean parameter vector ***μ***_*c*_ and a variance-covariance matrix **Σ** that is shared across cell types. The second equation models the probability of the *i*th cell belonging to the cell type *c* as depending on the underlying spatial domain. In particular, conditional on the *i*th cell belonging to the spatial domain *r*, ${c}_i^{(l)}$ follows a categorical distribution characterized by the *r*-domain-specific cell type composition vector ***π***_*r*_. The third equation models the spatial domain label of the *i*th cell on section *l*, ${z}_i^{(l)}$, as a function of the neighborhood graph *V*^(*l*)^ through a homogeneous Potts model characterized by an interaction parameter *β* [67, 68]. The Potts model encourages similarity in spatial domain label assignment for neighboring cells, thus allowing for smooth segmentation of the tissue into spatial domains. The probability mass function of the corresponding Potts model is defined as

4 $$\Pr \left({\boldsymbol{z}}^{(l)}|{V}^{(l)},\beta \right)=\frac{1}{C^{(l)}\left(\beta \right)}\exp \left\{\beta {\sum}_{i\sim {i}^{\prime }}I\left({z}_i^{(l)}={z}_{i^{\prime}}^{(l)}\right)\right\},$$

where *i* ∼ *i*^′^ denotes all neighboring pairs in the graph *V*^(*l*)^; $I\left({z}_i^{(l)}={z}_{i^{\prime}}^{(l)}\right)$ is an indicator function that equals 1 if both the *i*th and *i*^′^th cells belong to the same spatial domain and equals 0 otherwise; *β* is the interaction parameter that determines the extent of the spatial domain similarity among neighboring locations; and *C*^(*l*)^(*β*) is the normalizing constant, also known as the partition function that ensures the above probability mass function to have a summation of one across all possible configurations of ***z***^(*l*)^. Intuitively, the Potts model encourages spatial domain similarity on neighboring locations and borrows spatial domain information from neighboring locations to infer the spatial domain label on a location of focus, thus resulting in smoothed boundaries of the detected tissue regions.

We treat all the hyper-parameters in the above equations (***μ***_*c*_, **Σ**, ***π***_*r*_, *β*) as unknown and specify priors on the hyper-parameters in order to infer them based on the data at hand. The hyper-parameters in the first two equations (***μ***_*c*_, **Σ**, ***π***_*r*_) are relatively easy to infer algorithmically. We simply specify conjugate priors on them to facilitate computation. In particular, we specify a normal-gamma prior for ***μ***_*j*_ [69], an inverse-Wishart prior for **Σ**, and a Dirichlet distribution for ***π***_*r*_ (details in the Additional file 1: Supplementary notes). The hyper-parameter *β* in the Potts model, however, is difficult to infer algorithmically because of the normalization constant *C*^(*l*)^(*β*), which requires evaluating the probability mass function of the Potts model over all possible configurations of ***z***^(*l*)^ and is thus known to be NP hard. Indeed, because of the inference difficulty, previous relevant studies that use the Potts model often examine a set of pre-determined values of *β* and select a proper value among them based on visualization of the detected spatial domain [23, 25]. This approach requires knowing the spatial domain a priori, defeating our purpose of detecting spatial domain in a de novo fashion. Therefore, we treat *β* as unknown in the present study and seek to infer it along with the other hyper-parameters. In particular, we specify a uniform prior on *β* in the form of *Unif*(0, *β*_*max*_). The lower bound of zero in the uniform distribution represents one extreme case of a lack of smoothness in the detected spatial domain boundaries, as the spatial domain labels in the neighboring locations are no longer informative of the spatial domain label in the location of focus. We set the upper bound *β*_*max*_ in the uniform distribution to be a large number (set to be four here), representing the other extreme case where spatial location information is highly informative and where the resulting spatial domain boundaries are extremely smooth.

With the above model setup, we develop a Gibbs sampling algorithm in combination with a Metropolis-Hastings algorithm to perform parameter inference. Algorithm details are provided in the Additional file 1: Supplementary notes. Briefly, the sampling algorithm updates one parameter at a time based on its conditional distribution. The conditional distributions for the parameters other than ***z***^(*l*)^ and *β* are in known distributional forms and are sampled directly through Gibbs sampling. The conditional distribution of *β* is not straightforward to sample because of the normalization constant in the Potts model as explained above. Instead of computing the normalizing constant directly, we estimate the ratio of two normalizing constants by adapting the Swendsen-Wang algorithm [68, 70], which allows us to sample *β* from its conditional distribution through a Metropolis-Hastings algorithm. Inferring *β* enables adaptive and accurate detection of the spatial domain. In the presence of multiple tissue sections, we infer *β* based on the first tissue section to reduce the computational burden. Similarly, we sample ***z***^(*l*)^ from its conditional distribution by using the Swendsen-Wang algorithm to achieve a faster mixing rate. Importantly, we also post-process the sampling results to address the label switching issue [71] associated with the sampling of ***z*** and ***c*** in the corresponding mixture models. Label switching occurs in mixture models because the posterior distribution is invariant to the labeling of the mixture indicators ***z*** or ***c***. Consequently, the posterior samples can switch between the symmetric high posterior density areas, making it challenging to properly summarize the posterior samples. We deal with the label switching problem by post-processing the posterior samples based on the iterative version 1 of the equivalence class representation (ECR) algorithm [72] implemented in the label.switching package (version 1.8) [73]. Through our algorithm, we estimate the key parameters of interest that include the cell type labels ***c***, the cell type composition ***π***_*r*_ of the *r*th region, and spatial domain labels ***z***, across all tissue sections. These estimates allow us to comprehensively characterize the tissue architecture in a multi-scale and multi-sample fashion.

### Simulation design

#### Main simulations

We conducted extensive simulations to evaluate the performance of our method and compared it with existing approaches. To do so, we obtained the spatial locations of 1127 cells from the STARmap mouse cortex data (data details in the next section) and used the location information to allocate cells into four major spatial domains. These domains corresponded to distinct cortical layers and were obtained based on the expression patterns of cortical layer markers in the original study. We assumed that each spatial domain consisted of multiple cell types, and we set the total number of cell types on the tissue to be four for ease of simulation construction. We varied the composition of cell types in different spatial domains through creating four simulation scenarios. In scenario I, we assumed that each spatial domain only contained one cell type and that each cell type was only assigned to one domain. In the remaining three scenarios II–IV, we assumed that each spatial domain contained three cell types and that the missing cell type in each domain was different from those in the other spatial domains. In scenarios II and III, we selected one unique cell type in each spatial domain to be the dominant cell type. We set the probability of each cell belonging to the dominant cell type to be either 90% (scenario II) or 50% (scenario III) and set the probability of each cell belonging to the two non-dominant cell types to be either 5% (scenario II) or 25% (scenario III). In scenario IV, we set the probability of each cell belonging to either of the three cell types in each spatial domain to be equal (1/3). Therefore, scenario I represents one extreme case where one cell type completely dominates a spatial domain; scenario IV represents the other extreme case where the cell types in each spatial domain have equal compositions while scenarios II and III are in-between these two extreme cases. In each scenario, we assigned the cell type for each cell randomly from a categorical distribution with the corresponding probability vector being the cell type composition of the spatial domain where the cell resides.

With the cell type assignment, we simulated gene expression for each cell using the splatter package (version 1.16.1) [74], which provides gene expression data that largely resemble the real data (Additional file 1: Fig. S1). In splatter, we set the group parameter to be four and varied the number of genes (*nGenes*) to be either 200, 500, 800, or 1,000, resembling those typically captured by single-cell resolution spatial transcriptomic technologies [75]. We set the proportion of genes that are differentially expressed (DE) (*de*. *prob*) in each cell type versus the others to be either 0.1, 0.2, or 0.3. We set the DE strength, determined by *de*. *facloc*, to be 0.5, 0.7, 1.1, or 1.4, corresponding to 1.5-fold, two-fold, three-fold, or four-fold change in expression. We randomly selected an equal proportion of DE genes to be upregulated or downregulated. We set the other parameters, including the library size parameters and biological coefficient of variation (BCV) for incorporating the mean-variance trend, all based on 111 eL2/3 cells from the STARmap data, using the splatEstimate function provided in the splatter package.

For each simulation scenario described above, we set a baseline simulation setting with *nGenes* set to be 200, *de*. *prob* set to be 0.2, and *de*. *facloc* set to be 1.1. We then varied each of the three parameters (*nGenes*, *de*. *prob*, *de*. *facloc*) one at a time on top of the baseline scenarios to examine the influence of each parameter on method performance. We examined a total of eight simulation settings for each scenario, with 50 simulation replicates in each setting. We then applied BASS on the expression features along with spatial localization information to detect spatial domains, infer cell types, and estimate domain-specific cell type compositions. We evaluated the performance of spatial domain detection and cell type inference using the adjusted random index (ARI), which measures the similarity between the estimated spatial domain or cell type labels and the truth. We also evaluated the estimation accuracy of the cell type compositions across spatial domains by comparing the estimated composition to the underlying truth. Because the inference results are invariant to a permutation of cell type labels or spatial domain labels, the cell type or spatial domain indexes are arbitrary. For example, referring to a group of cells as cell type 1 is the same as referring to the same group of cells as cell type 2. Therefore, we could not directly compare the estimated cell type composition in each inferred spatial domain with the underlying truth. Instead, we first identified the permutation of the estimated spatial domain labels that best matched the truth as well as the permutation of the estimated cell type labels that best matched the truth, before comparing the cell type composition in each domain with the ground truth.

In addition to simulating and analyzing a single tissue section, we also simulated multiple tissue sections and evaluated the performance of BASS for multi-sample integrative analysis. Specifically, we generated additional tissue sections based on the same spatial locations of the 1127 cells but with slightly different spatial domain allocations, creating varying spatial domain boundaries between the four cortical layers on different tissue sections. Specifically, we set the number of tissue sections (*L*) to be either 1, 2, 5, or 10. In each section, we set the spatial domain boundaries to be vertical and created new boundaries by horizontally moving the original boundaries based on a uniform distribution with step size set to be approximately 10% (= 500) of the tissue width (Additional file 1: Fig. S2). We followed similar procedures to assign cell types in each scenario and simulated gene expression for each cell under the baseline simulation setting and a more challenging setting (with *de*. *facloc* set to be 0.7, on top of the baseline setting) using the splatter package. For each simulation setting, we performed 50 simulation replicates. In each replicate, we applied BASS to jointly analyze all tissue sections for spatial domain detection, cell type inference, and estimation of cell type compositions across spatial domains. We again evaluated the performance of spatial domain detection and cell type inference using ARI and evaluated the estimation accuracy of the cell type compositions by comparing the estimated composition to the underlying truth using root mean square error (RMSE).

#### Additional simulations

Besides the main simulations described above, we also performed additional simulations to evaluate the influence of various other factors—including the specified number of cell types and spatial domains, rare cell types, and a random exclusion of genes—on method performance, as detailed below.

##### Influence of the specified number of cell types and spatial domains

We performed additional simulations to evaluate the influence of the specified number of cell types and the number of spatial domains on method performance. Specifically, we focused on the baseline simulation setting of scenario III and specified either the number of cell types or the number of spatial domains to be 2, 4, 6, 8, or 10 while fixing the other to be the truth (4). We then evaluated the impact of the two parameters on both cell type clustering and spatial domain detection for BASS and the corresponding task for the other methods based on three criteria: the overall agreement between the estimated labels and true labels measured by ARI and NMI; the number of estimated cell types and/or spatial domains; and the proportion of cells in each cell type cluster and/or spatial domain.

##### Influence of rare cell types

We created additional simulations to evaluate the performance of all methods in the presence of rare cell types. Specifically, we assumed that the tissue consisted of four major cell types along with either six or ten rare cell types. We assumed that the major cell types consisted of 70% of cells in the data while the rare cell types consisted of the remaining 30% of cells. This way, each rare cell type consisted of 5% (~ 56 cells) or 3% (~ 34 cells) of the total cell population. We assumed that the composition of major cell types in different spatial domains was the same as that in the scenario III, where each spatial domain contained three major cell types with a 2:1:1 ratio. For rare cell types, we examined two settings where the rare cell types exhibit either a random distribution pattern or a domain-specific pattern. In the setting of a random distribution pattern, we assumed that the rare cell types were randomly distributed across the entire tissue. In the setting of a domain-specific pattern, we assumed that each rare cell type was located only in one spatial domain. In these rare cell type simulations, in addition to ARI, we also evaluated the performance of all methods using the *F*_1_ score and Matthews correlation coefficient (MCC) following previous papers on rare cell type clustering [76–78].

##### Influence of a random exclusion of genes

Finally, because spatial transcriptomic technologies vary widely in terms of the number of genes they can profile, we conducted additional simulations, where genes were randomly excluded from the gene expression matrix during model fitting, to understand its influence on the performance of all methods. Specifically, we focused on the setting where the number of genes was set to be 1000 and where the other parameters were set to be the baseline for all four simulation scenarios. We randomly excluded genes from the simulated expression count matrix and evaluated settings where 100, 200, or 500 genes were retained.

### Method comparison

We compared BASS with several existing methods for spatial domain detection and cell type clustering. For spatial domain detection, we compared BASS with HMRF [23], BayesSpace [25], and SpaGCN [24]. Both HMRF and BayesSpace rely on a Potts model to impose a spatial dependency structure among neighboring cells, while SpaGCN relies on a graph convolutional network and constructs a weighted undirected graph to model the spatial dependency among cells. We used the Giotto package (version 1.0.4) [79] to fit HMRF and followed the online tutorial of Giotto for data pre-processing. Specifically, we excluded cells that did not contain any expressed gene and excluded genes that were not expressed in any cell. We normalized the count matrix for the remaining cells and genes with a default scale factor of 6000 and performed log-transformation. We then selected the top 100 genes with spatial coherent expression patterns as measured by the BinSpect-kmeans (Binary Spatial Extraction) method in Giotto. Afterwards, we constructed a spatial network with the default Delaunay method and fitted the HMRF model with the Potts parameter *β* set to be a fixed value, which ranged from 0 to 50 at increments of 2. We show HMRF results in simulations for three different *β*s that correspond to the worst, median, and best performance in terms of the median ARI across 50 simulation replicates. In the real data, we determined the optimal *β* to be the value that gives rise to the highest ARI compared to the ground truth obtained through the manual annotation—thus, the results of HMRF in the real data are likely overly optimistic. For BayesSpace, we used the BayesSpace package (version 1.2.1) with default settings and followed the online tutorial of BayesSpace for model fitting. For SpaGCN, we used the SpaGCN package (version 1.2.0) with default settings and followed the online tutorial of SpaGCN for model fitting. In particular, the spatial parameter (*p*), representing the percentage of total expression contributed by neighborhoods, was set to be its recommended value of 0.5. Note that all the above compared methods could only analyze one tissue section at a time. Therefore, we only compared the multiple-section version of BASS with the single-section version of BASS to illustrate the benefits of integrative analysis of multiple tissue sections.

For cell type clustering, we compared BASS with Seurat [27], SC3 [28], and FICT [31]. Both Seurat and SC3 are commonly used for cell type clustering in single-cell RNA-seq studies and have been shown to outperform a wide range of cell type clustering methods as demonstrated in two recent benchmarking papers [29, 30]. For Seurat, we used the Seurat package (version 3.2.3) for model fitting. We followed the Seurat online tutorial to normalize and scale the expression count data and extracted top 20 PCs as expression features for the subsequent clustering analysis. Seurat uses a resolution parameter to indirectly determine the number of clusters. We ran Seurat on a range of resolution parameters (0.1 to 4 at increments of 0.1) and identified the first value that resulted in the desired number of cell types. For SC3, we used the SC3 package (version 1.20.0) with default settings for model fitting. For FICT, we followed the sample code with its recommended settings for model fitting. Finally, we extended Seurat and SC3 to make use of the expression data from multiple tissue sections. Specifically, we extracted a common set of genes from all tissue sections and simply stacked the expression matrix from all sections to serve as the input. We then applied Seurat and SC3 on the combined data for cell type clustering through their own analytic pipelines. As FICT can only analyze one tissue section at a time, we did not include FICT in our comparison when analyzing multiple tissue sections.

### Differential gene expression analysis and cell type annotation

We performed differential expression (DE) analysis to identify marker genes for each cell type cluster inferred by BASS and the other methods. In particular, we used the Seurat package (version 3.2.3) [27] to examine one gene at a time and used a Wilcoxon rank-sum test to identify genes that were DE in one cell type as compared to the remaining cell types. Then, we annotated each cell type by comparing the identified DE genes with previously known cell type marker genes.

### Mouse medial prefrontal cortex data by STARmap

We obtained the STARmap data based on the online resources provided in the original study [10]. For single tissue section analysis, we focused on the tissue section “BZ5” that measured the medial prefrontal cortex (mPFC) region of the mouse brain. The data contains expression values in terms of count for 166 genes measured on 1127 single cells along with their centroid coordinates on the tissue. Among the 166 genes, 112 of them are putative cell-type markers and 48 of them are activity-regulated genes. After removing 78 “NA” cells, which were not confidently identified to be any cell type, we retained a total of 166 genes measured on 1049 single cells for further analysis. Following [24, 80], we assigned the cells into four spatial domains that included the cortical layers L1 (86 cells), L2/3 (167 cells), L5 (449 cells), and L6 (347 cells), based on the spatial expression patterns of marker genes that included *Bgn* (L1), *Cux2* (L2/3), *Tcerg1l* (L5), and *Pcp4* (L6). Note that mPFC lacks the L4 cortical layer [81]. In addition, we obtained cell type labels for all cells from the original study [10] to serve as the ground truth for evaluating the performance of cell type clustering. A total of 15 cell types were described in the original study, and these cell types were obtained through repeated clustering with careful marker gene examination.

For multiple tissue section analysis, we obtained two additional tissue sections “BZ9” and “BZ14” that were measured on the same mPFC region from different mice. We followed the same procedure described above to filter cells and retained the same set of 166 genes measured on 1053 cells (BZ9) and 1088 cells (BZ14) along with their centroid coordinates for further analysis. Similarly, cells on the two additional tissue sections were carefully annotated to the four cortical layers. In the analysis, we set the number of spatial domains to be the truth (four) and set the number of cell types to be 15 following [10] for all methods for the analyses on a single tissue section as well as the integrative analysis across multiple tissue sections.

### Mouse hypothalamus data by MERFISH

We obtained the MERFISH data set that measured the mouse preoptic region of the hypothalamus from Dryad [16]. For single tissue section analysis, we focused on the tissue section at Bregma-0.14 mm from animal 1. The data contains expression values of 160 genes measured on 6605 single cells along with their centroid coordinates on the tissue. In the original study [16], among the 160 genes, 85 of them were pre-selected as either known markers for major cell classes or relevant to neuronal functions of the hypothalamus; 70 were identified with scRNA-seq as neuronal cluster markers; and the remaining 5 genes represented the measurement of barcodes not assigned to any RNA and served as blank controls. Among the 6605 cells, 679 cells were annotated to be the “Ambiguous” class as they were identified as putative doublets [16]. After removing the blank genes and ambiguous cells, we retained a total of 155 genes measured on 5926 single cells for further analysis. The downloaded data contained normalized gene expression values, which were previously computed as the total counts per cell divided by the cell volume (for 135 genes measured by the combinatorial smFISH) or by the arbitrary fluorescence units per μm^3^ (for 20 genes measured by the non-combinatorial, sequential FISH) and further scaled by 1000 [16]. In addition, we assigned the cells to 8 spatial domains based on spatial gene expression patterns and the histology diagram of the mouse brain from the Allen’s brain atlas [80]. These spatial domains included the third ventricle (V3; 311 cells), bed nuclei of the strata terminalis (BST; 1539 cells), columns of the fornix (fx; 400 cells), medial preoptic area (MPA; 1655 cells), medial preoptic nucleus (MPN; 824 cells), periventricular hypothalamic nucleus (PV; 211 cells), paraventricular hypothalamic nucleus (PVH; 703 cells), and paraventricular nucleus of the thalamus (PVT; 283 cells). In addition, we obtained the cell type labels from the original study [16] to evaluate the performance of cell type clustering.

For multiple tissue section analysis, we obtained four additional tissue sections adjacent to Bregma-0.14 mm from the same animal. These include tissue sections at Bregma-0.04, -0.09, -0.19, and -0.24. We followed the same procedure described above to filter genes and cells. We retained the same set of 155 genes measured on 5488 cells (Bregma-0.04), 5557 cells (Bregma-0.09), 5803 cells (Bregma-0.19), and 5543 cells (Bregma-0.24) along with their centroid coordinates for further analysis. Similarly, cells on the four additional tissue sections were carefully annotated to the eight brain structures in the hypothalamus. For both the analyses of a single tissue section and the integrative analysis of multiple tissue sections, we set the number of spatial domains to be the truth (8) and the number of cell types to be 20 for all methods.

### Human dorsolateral prefrontal cortex data by 10x Visium

We obtained the human dorsolateral prefrontal cortex (DLPFC) data set that was measured on the 10x Visium platform [32]. DLPFC data contains expression values of 33,538 genes measured on two pairs of tissue sections from three independent neurotypical adult donors. Each pair consists of two directly adjacent, 10 μm serial tissue sections with the second pair located 300 μm posterior to the first, resulting in a total of 12 tissue sections. We excluded spots that were not mapped to the tissue region in the histology image and retained a total of 33,538 genes measured on 4226 (151507), 4384 (151508), 4789 (151509), 4634 (151510), 3661 (151669), 3498 (151670), 4110 (151671), 4015 (151672), 3639 (151673), 3673 (151674), 3592 (151675), and 3460 (151676) spots along with their spatial locations for further analysis. In addition, we obtained manually annotated labels of seven laminar clusters that included six cortical layers from L1 to L6 and white matter (WM) from the original publication as our ground truth to evaluate the performance of spatial domain detection. We focused our analysis only on spatial domain detection because the clustering of spatial spots no longer has the cell type interpretation. For single tissue section analysis, we analyzed each of the 12 tissue sections separately. For multiple tissue section analysis, we jointly analyzed four tissue sections from each adult donor because they contained similar tissue structures. For both the analysis on a single tissue section and the integrative analysis of multiple tissue sections, we set the number of spatial domains to be the truth (7) for all methods. In addition, for BASS, we extracted the top 3000 spatially variable genes with SPARK-X [22] before performing dimension reduction. We set the number of cell types to a relatively large number (15) to capture the expression heterogeneity.

## Supplementary information

Additional file 1. Supplementary information. Supplementary figures and table. Supplementary notes describing the detailed derivations of the BASS algorithm.

Additional file 2. Review History.

## Data Availability

BASS is implemented as an R package with underlying efficient C++ code interfaced through Rcpp. The software BASS, together with code for reproducing all the analysis results presented in the present study are freely available at GitHub [82] and Zenodo [83]. The source code is released under the GNU General Public License v3.0. All data used in this paper are publicly available. The mouse medial prefrontal cortex dataset by STARmap is available at the STARmap Resources, https://www.starmapresources.org/data [84]. The mouse hypothalamus dataset by MERFISH is available at the DRYAD, https://datadryad.org/stash/dataset/doi:10.5061/dryad.8t8s248 [85]. The DLPFC dataset by 10x Visium is available at the spatialLIBD project website, http://research.libd.org/spatialLIBD/ [86].
