# Supplementary material for: BASS: multi-scale and multi-sample analysis enables accurate cell type clustering and spatial domain detection in spatial transcriptomic studies
Source: Genome Biol. 2022 Aug 4;23:168. doi: 10.1186/s13059-022-02734-7 (PMC9351148; doi:10.1186/s13059-022-02734-7)
Supplement: Supplementary file 1 — Additional file 1. Supplementary information. Supplementary figures and table. Supplementary notes describing the detailed derivations of the BASS algorithm. [file 13059_2022_2734_MOESM1_ESM.pdf]

Supplementary figures:

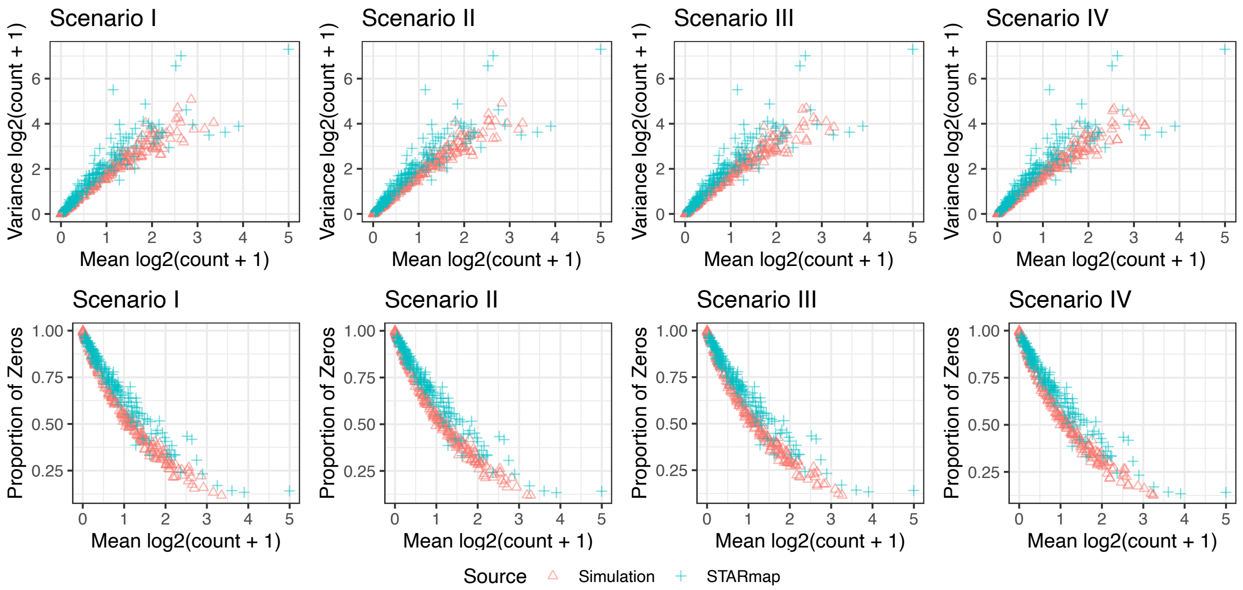

Fig. S1 Comparison of the simulated datasets under different scenarios with the STARmap dataset. One replicate was generated under the baseline setting of each simulation scenario (I-IV) and was compared to the STARmap dataset. The scatter plots on the top row show the mean-variance relationship of each gene. The scatter plots on the bottom row show the relationship between the mean expression of a gene and the percentage of zeros.

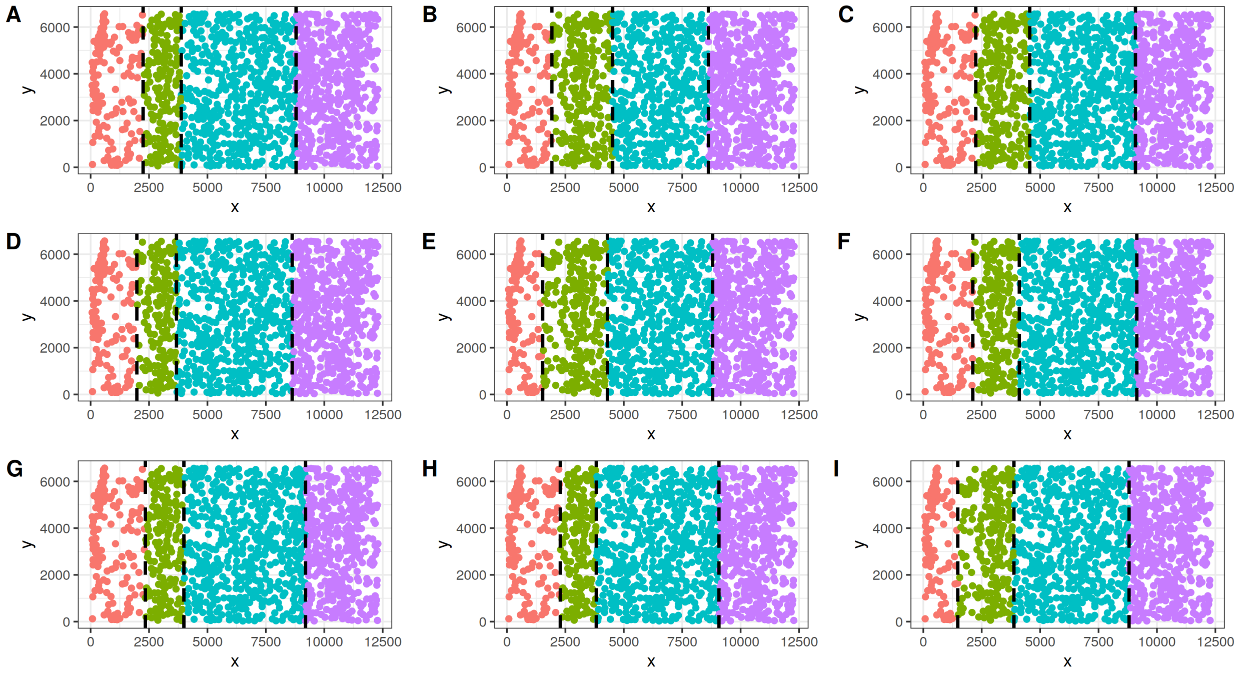

Fig. S2 Additional tissue sections generated based on the same spatial locations of cells from tissue section BZ5 in STARmap data. In each section, the spatial domain boundaries are set to be vertical and horizontally moved by changing its distance to the original boundary, where the distance is generated based on a uniform distribution with step size set to be approximately 10% (500) of the tissue width.

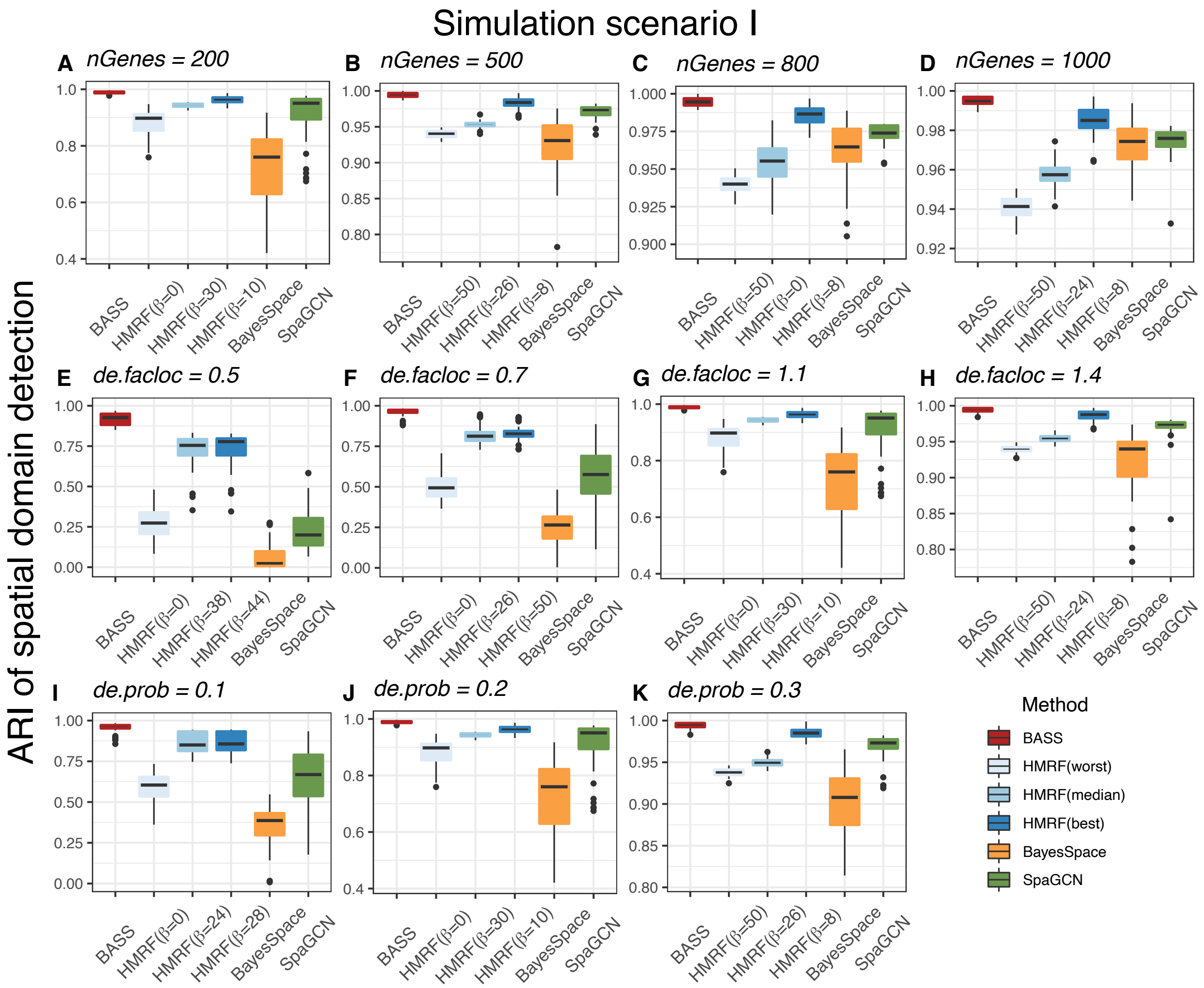

Fig. S3 Comparison of different methods for spatial domain detection in simulation (scenario I) with a single tissue section. Boxplots of ARI show the accuracy of different methods for spatial domain detection. Compared methods for spatial domain detection include BASS, HMRF, BayesSpace, and SpaGCN. For HMRF, a list of the spatial parameter β ranging from 0 to 50 at increments of 2 are examined and the three β values that corresponded to the worst, median and best performance are displayed. Simulations were carried out under different number of genes: **(A)** $nGenes = 200$, **(B)** $nGenes = 500$, **(C)** $nGenes = 800$, and **(D)** $nGenes = 1000$; different DE gene strength: **(E)** $de.facloc = 0.5$, **(F)** $de.facloc = 0.7$, **(G)** $de.facloc = 1.1$, and **(H)** $de.facloc = 1.4$; and different proportions of genes that were differentially expressed in each cell type versus the others: **(I)** $de.prob = 0.1$, **(J)** $de.prob = 0.2$, and **(K)** $de.prob = 0.3$ on top of the baseline parameter setting: $nGenes = 200$, $de.prob = 0.2$, and $de.facloc = 1.1$.

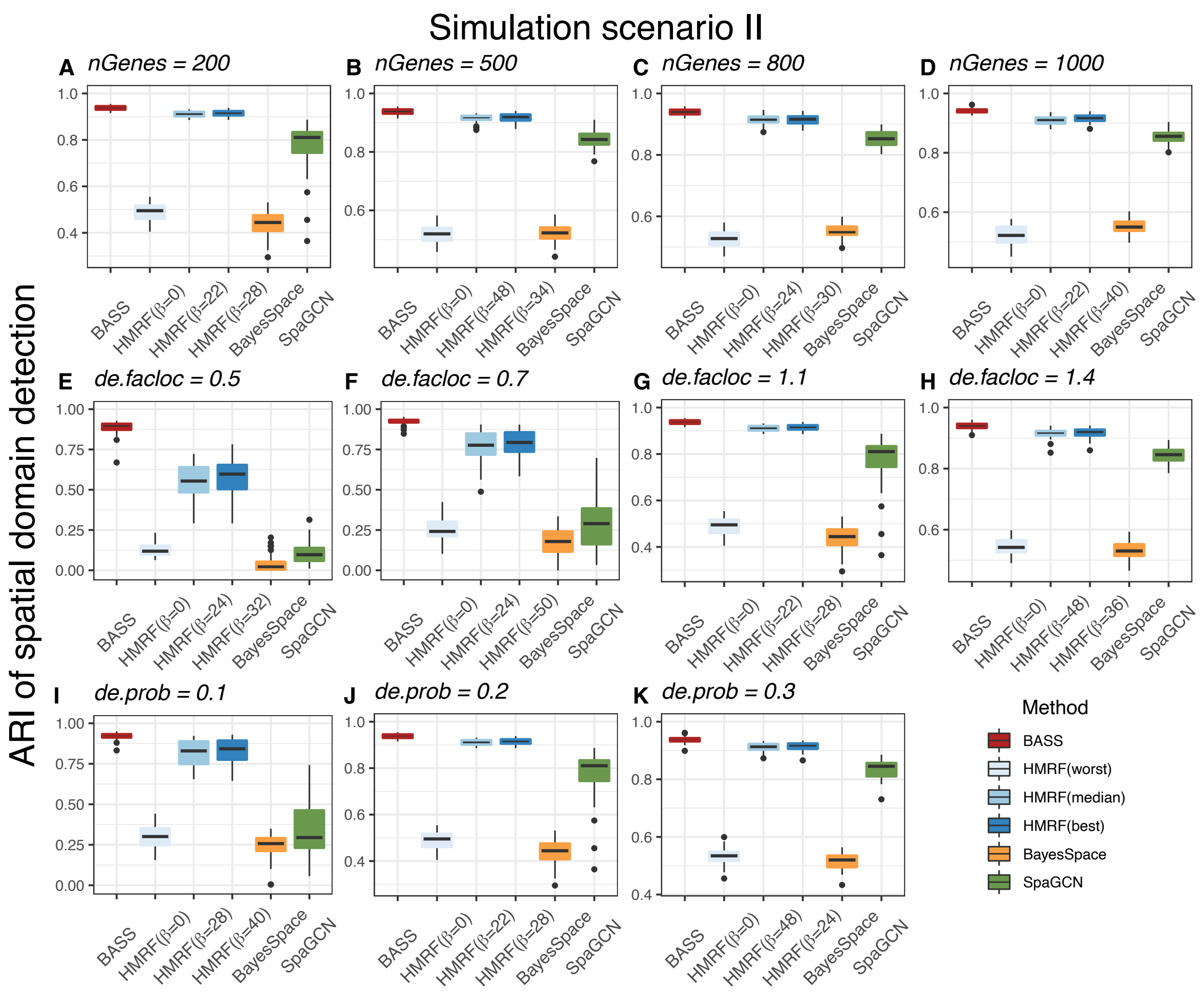

Fig. S4 Comparison of different methods for spatial domain detection in simulation (scenario II) with a single tissue section. Boxplots of ARI show the accuracy of different methods for spatial domain detection. Compared methods for spatial domain detection include BASS, HMRF, BayesSpace, and SpaGCN. For HMRF, a list of the spatial parameter β ranging from 0 to 50 at increments of 2 are examined and the three β values that corresponded to the worst, median and best performance are displayed. Simulations were carried out under different number of genes: **(A)** $nGenes = 200$, **(B)** $nGenes = 500$, **(C)** $nGenes = 800$, and **(D)** $nGenes = 1000$; different DE gene strength: **(E)** $de.facloc = 0.5$, **(F)** $de.facloc = 0.7$, **(G)** $de.facloc = 1.1$, and **(H)** $de.facloc = 1.4$; and different proportions of genes that were differentially expressed in each cell type versus the others: **(I)** $de.prob = 0.1$, **(J)** $de.prob = 0.2$, and **(K)** $de.prob = 0.3$ on top of the baseline parameter setting: $nGenes = 200$, $de.prob = 0.2$, and $de.facloc = 1.1$.

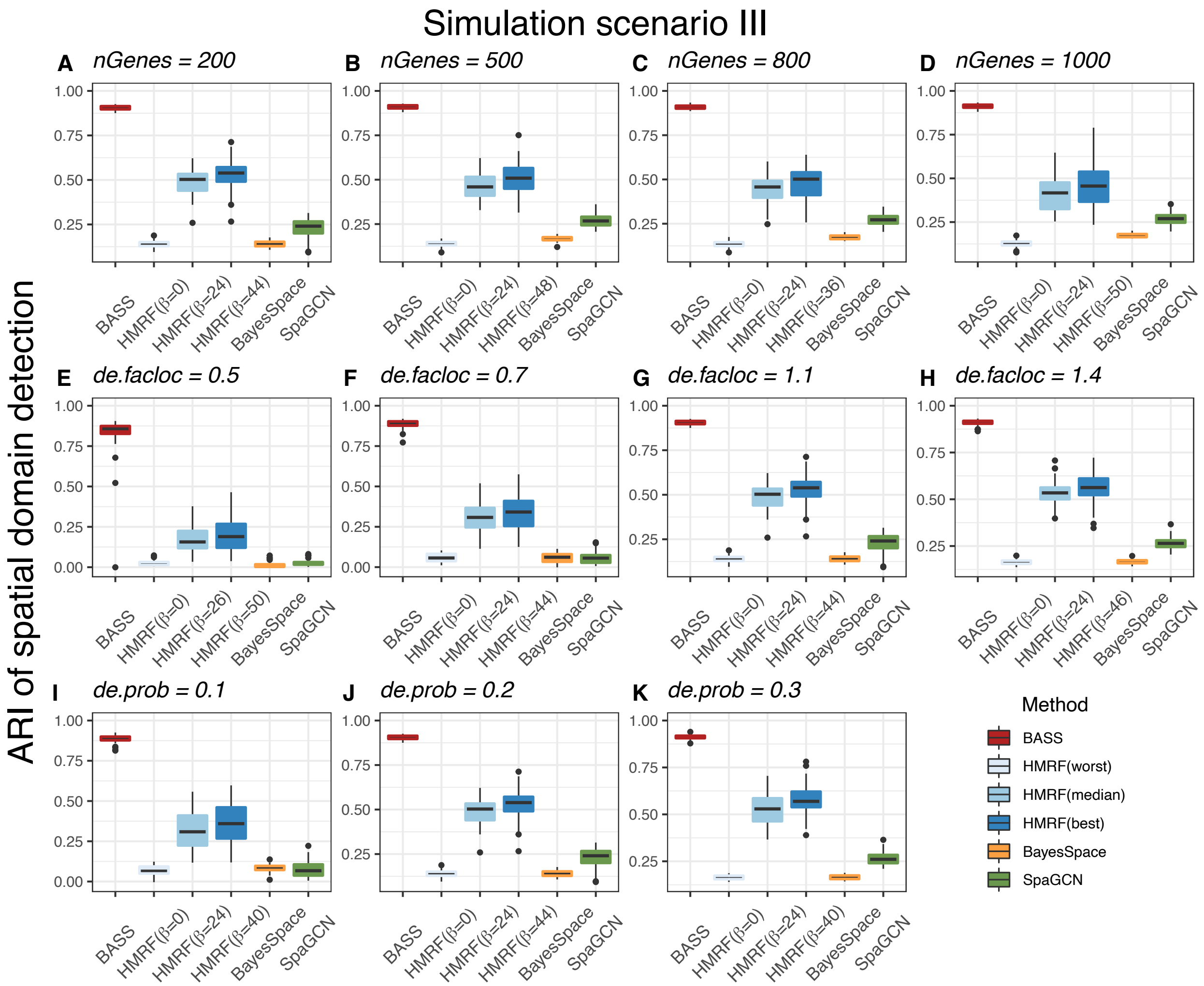

Fig. S5 Comparison of different methods for spatial domain detection in simulation (scenario III) with a single tissue section. Boxplots of ARI show the accuracy of different methods for spatial domain detection. Compared methods for spatial domain detection include BASS, HMRF, BayesSpace, and SpaGCN. For HMRF, a list of the spatial parameter β ranging from 0 to 50 at increments of 2 are examined and the three β values that corresponded to the worst, median and best performance are displayed. Simulations were carried out under different number of genes: **(A)** $nGenes = 200$, **(B)** $nGenes = 500$, **(C)** $nGenes = 800$, and **(D)** $nGenes = 1000$; different DE gene strength: **(E)** $de.facloc = 0.5$, **(F)** $de.facloc = 0.7$, **(G)** $de.facloc = 1.1$, and **(H)** $de.facloc = 1.4$; and different proportions of genes that were differentially expressed in each cell type versus the others: **(I)** $de.prob = 0.1$, **(J)** $de.prob = 0.2$, and **(K)** $de.prob = 0.3$ on top of the baseline parameter setting: $nGenes = 200$, $de.prob = 0.2$, and $de.facloc = 1.1$.

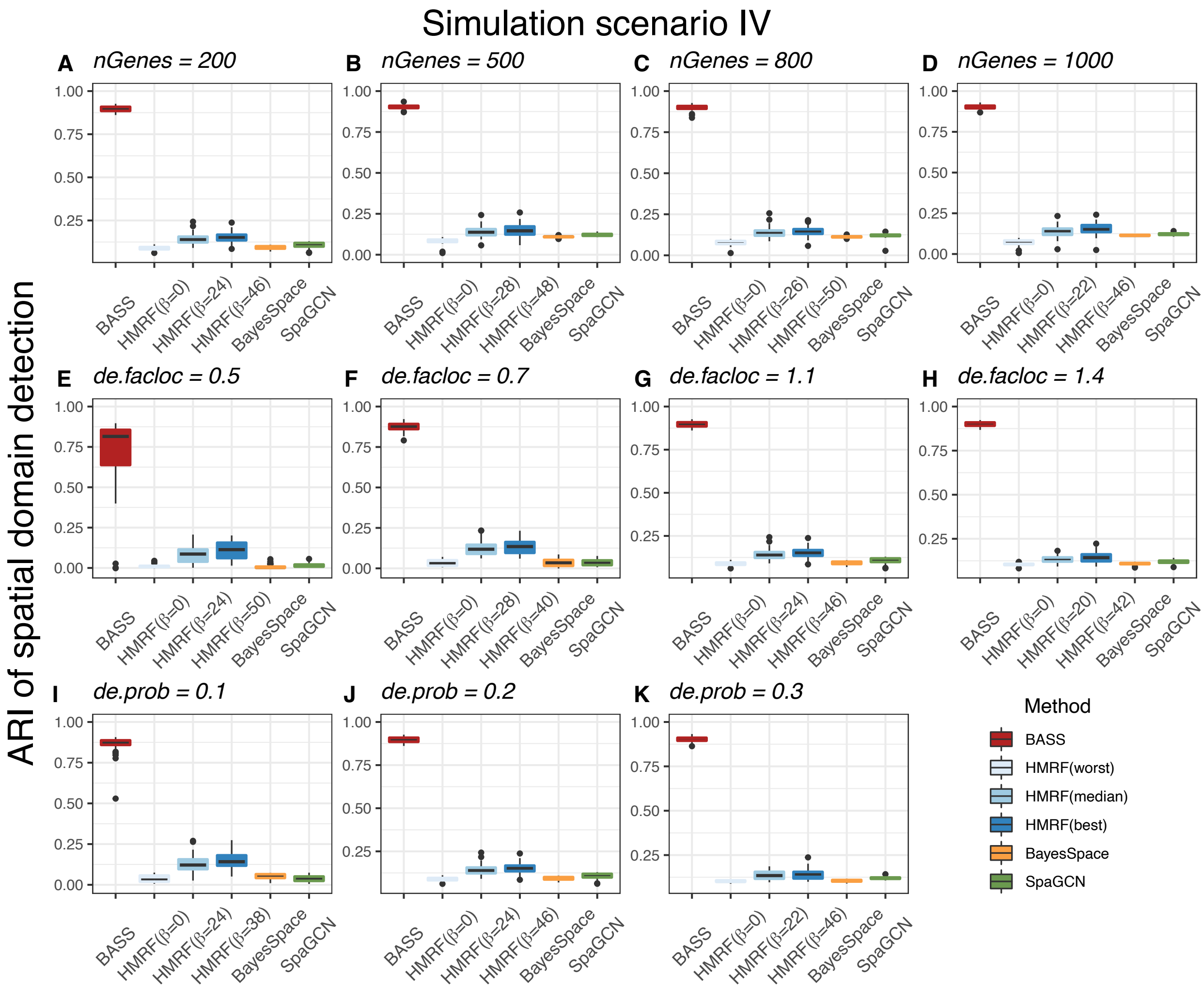

Fig. S6 Comparison of different methods for spatial domain detection in simulation (scenario IV) with a single tissue section. Boxplots of ARI show the accuracy of different methods for spatial domain detection. Compared methods for spatial domain detection include BASS, HMRF, BayesSpace, and SpaGCN. For HMRF, a list of the spatial parameter β ranging from 0 to 50 at increments of 2 are examined and the three β values that corresponded to the worst, median and best performance are displayed. Simulations were carried out under different number of genes: **(A)** $nGenes = 200$, **(B)** $nGenes = 500$, **(C)** $nGenes = 800$, and **(D)** $nGenes = 1000$; different DE gene strength: **(E)** $de.facloc = 0.5$, **(F)** $de.facloc = 0.7$, **(G)** $de.facloc = 1.1$, and **(H)** $de.facloc = 1.4$; and different proportions of genes that were differentially expressed in each cell type versus the others: **(I)** $de.prob = 0.1$, **(J)** $de.prob = 0.2$, and **(K)** $de.prob = 0.3$ on top of the baseline parameter setting: $nGenes = 200$, $de.prob = 0.2$, and $de.facloc = 1.1$.

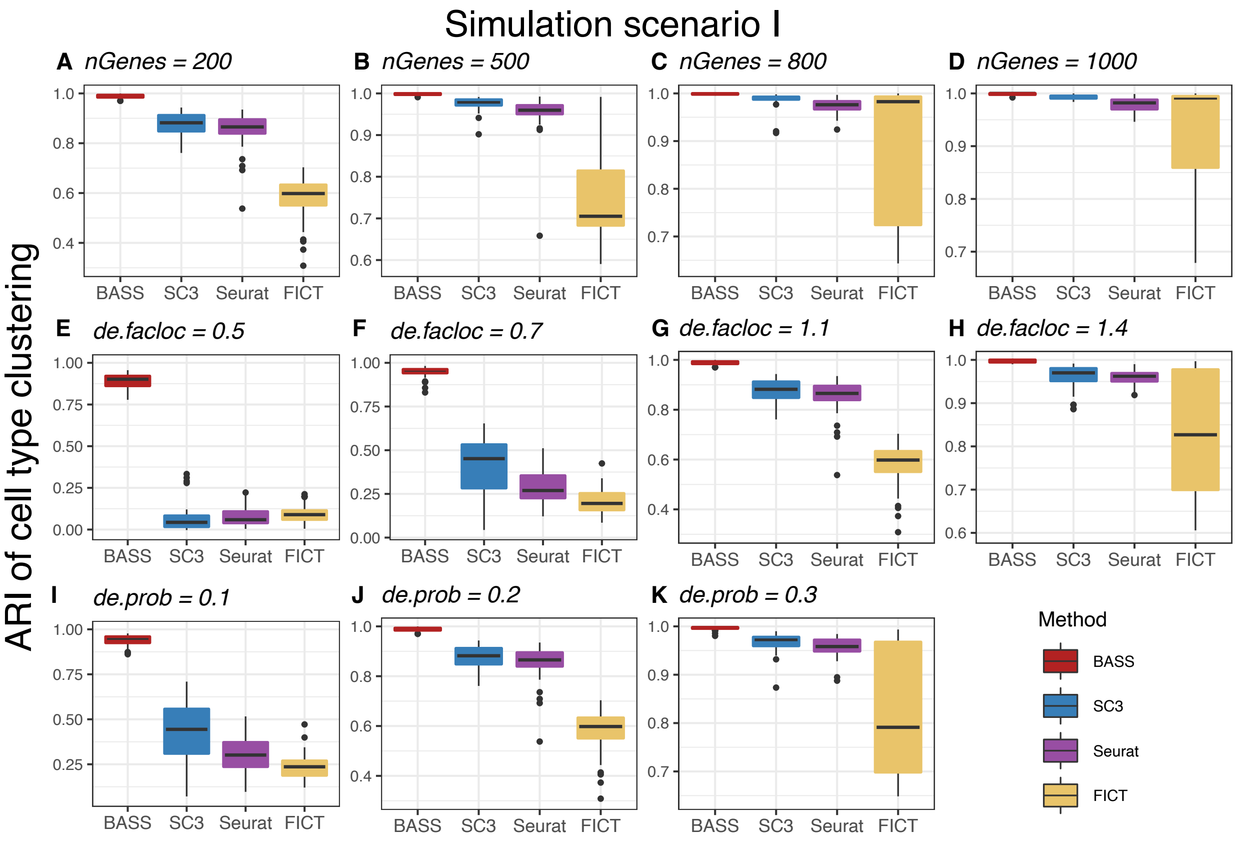

Fig. S7 Comparison of different methods for cell type clustering in simulation (scenario I) with a single tissue section. Boxplots of ARI show the accuracy of different methods for cell type clustering. Compared methods for cell type clustering include BASS, Seurat, SC3, and FICT. Simulations were carried out under different number of genes: (**A**) $nGenes = 200$, (**B**) $nGenes = 500$, (**C**) $nGenes = 800$, and (**D**) $nGenes = 1000$; different DE gene strength: (**E**) $de.facloc = 0.5$, (**F**) $de.facloc = 0.7$, (**G**) $de.facloc = 1.1$, and (**H**) $de.facloc = 1.4$; and different proportions of genes that were differentially expressed in each cell type versus the others: (**I**) $de.prob = 0.1$, (**J**) $de.prob = 0.2$, and (**K**) $de.prob = 0.3$ on top of the baseline parameter setting: $nGenes = 200$, $de.prob = 0.2$, and $de.facloc = 1.1$.

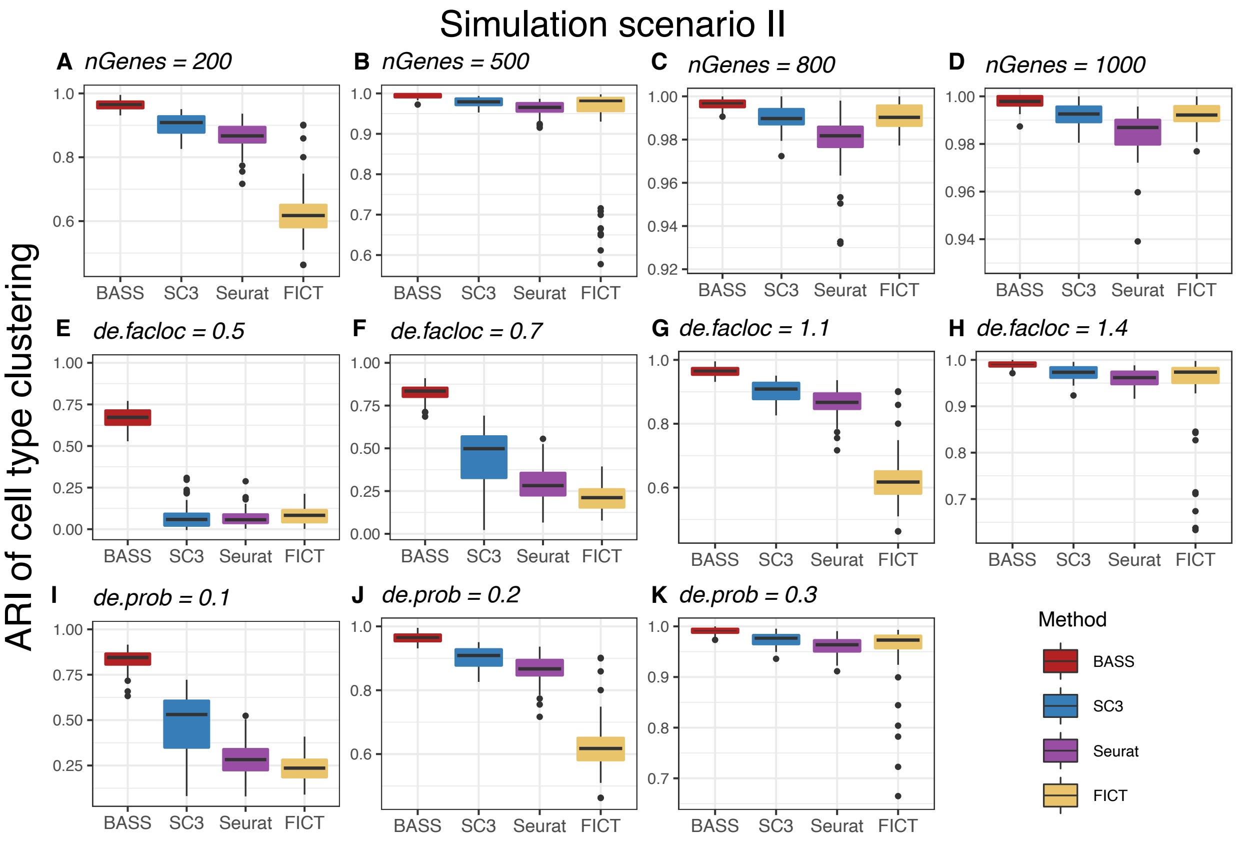

Fig. S8 Comparison of different methods for cell type clustering in simulation (scenario II) with a single tissue section. Boxplots of ARI show the accuracy of different methods for cell type clustering. Compared methods for cell type clustering include BASS, Seurat, SC3, and FICT. Simulations were carried out under different number of genes: (**A**) $nGenes = 200$, (**B**) $nGenes = 500$, (**C**) $nGenes = 800$, and (**D**) $nGenes = 1000$; different DE gene strength: (**E**) $de.facloc = 0.5$, (**F**) $de.facloc = 0.7$, (**G**) $de.facloc = 1.1$, and (**H**) $de.facloc = 1.4$; and different proportions of genes that were differentially expressed in each cell type versus the others: (**I**) $de.prob = 0.1$, (**J**) $de.prob = 0.2$, and (**K**) $de.prob = 0.3$ on top of the baseline parameter setting: $nGenes = 200$, $de.prob = 0.2$, and $de.facloc = 1.1$.

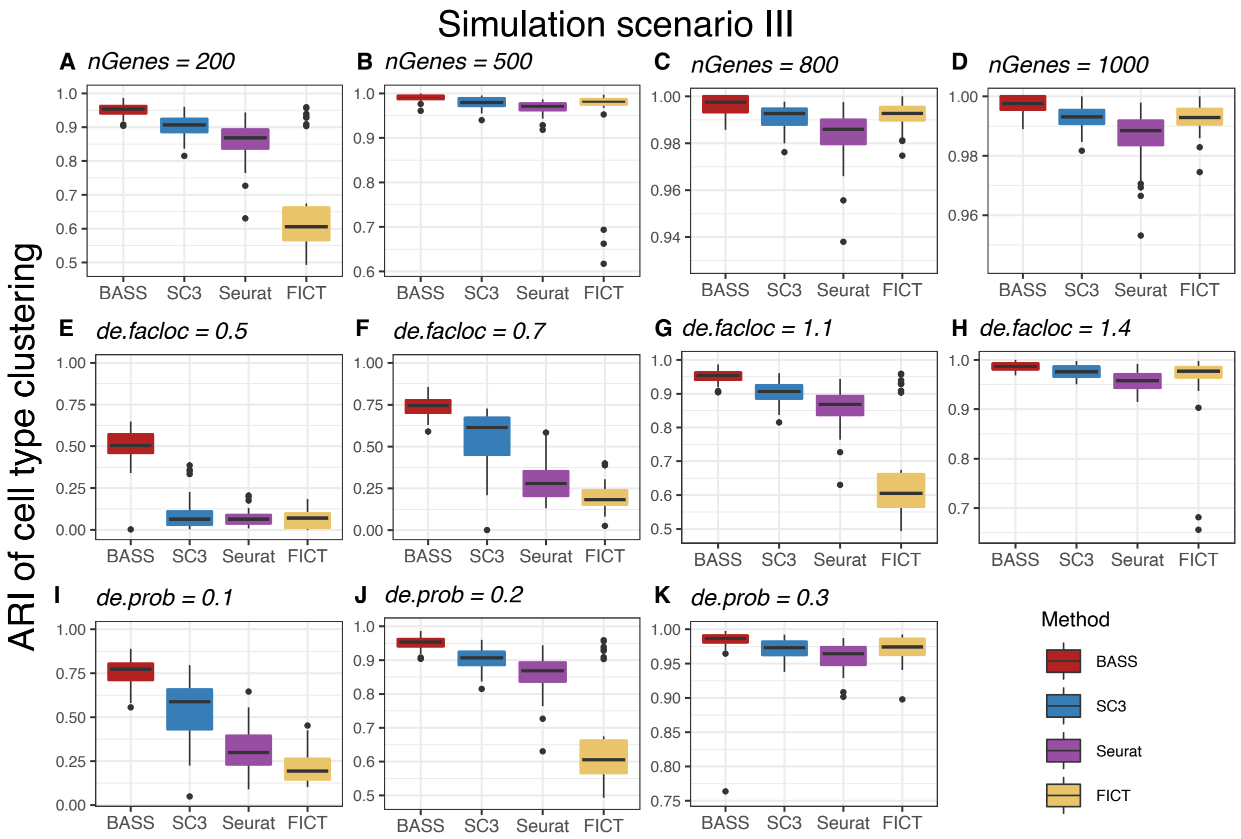

Fig. S9 Comparison of different methods for cell type clustering in simulation (scenario III) with a single tissue section. Boxplots of ARI show the accuracy of different methods for cell type clustering. Compared methods for cell type clustering include BASS, Seurat, SC3, and FICT. Simulations were carried out under different number of genes: (**A**) $nGenes = 200$, (**B**) $nGenes = 500$, (**C**) $nGenes = 800$, and (**D**) $nGenes = 1000$; different DE gene strength: (**E**) $de.facloc = 0.5$, (**F**) $de.facloc = 0.7$, (**G**) $de.facloc = 1.1$, and (**H**) $de.facloc = 1.4$; and different proportions of genes that were differentially expressed in each cell type versus the others: (**I**) $de.prob = 0.1$, (**J**) $de.prob = 0.2$, and (**K**) $de.prob = 0.3$ on top of the baseline parameter setting: $nGenes = 200$, $de.prob = 0.2$, and $de.facloc = 1.1$.

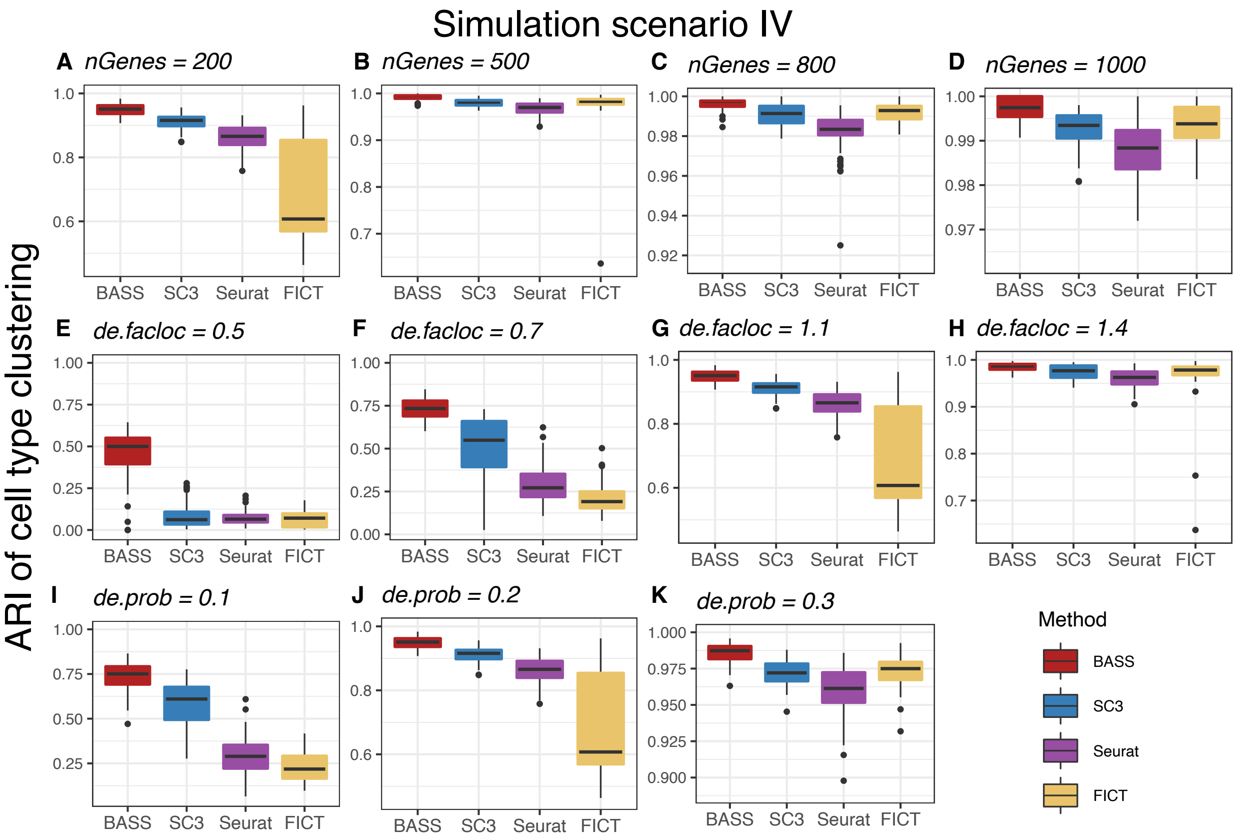

Fig. S10 Comparison of different methods for cell type clustering in simulation (scenario IV) with a single tissue section. Boxplots of ARI show the accuracy of different methods for cell type clustering. Compared methods for cell type clustering include BASS, Seurat, SC3, and FICT. Simulations were carried out under different number of genes: (**A**) $nGenes = 200$, (**B**) $nGenes = 500$, (**C**) $nGenes = 800$, and (**D**) $nGenes = 1000$; different DE gene strength: (**E**) $de.facloc = 0.5$, (**F**) $de.facloc = 0.7$, (**G**) $de.facloc = 1.1$, and (**H**) $de.facloc = 1.4$; and different proportions of genes that were differentially expressed in each cell type versus the others: (**I**) $de.prob = 0.1$, (**J**) $de.prob = 0.2$, and (**K**) $de.prob = 0.3$ on top of the baseline parameter setting: $nGenes = 200$, $de.prob = 0.2$, and $de.facloc = 1.1$.

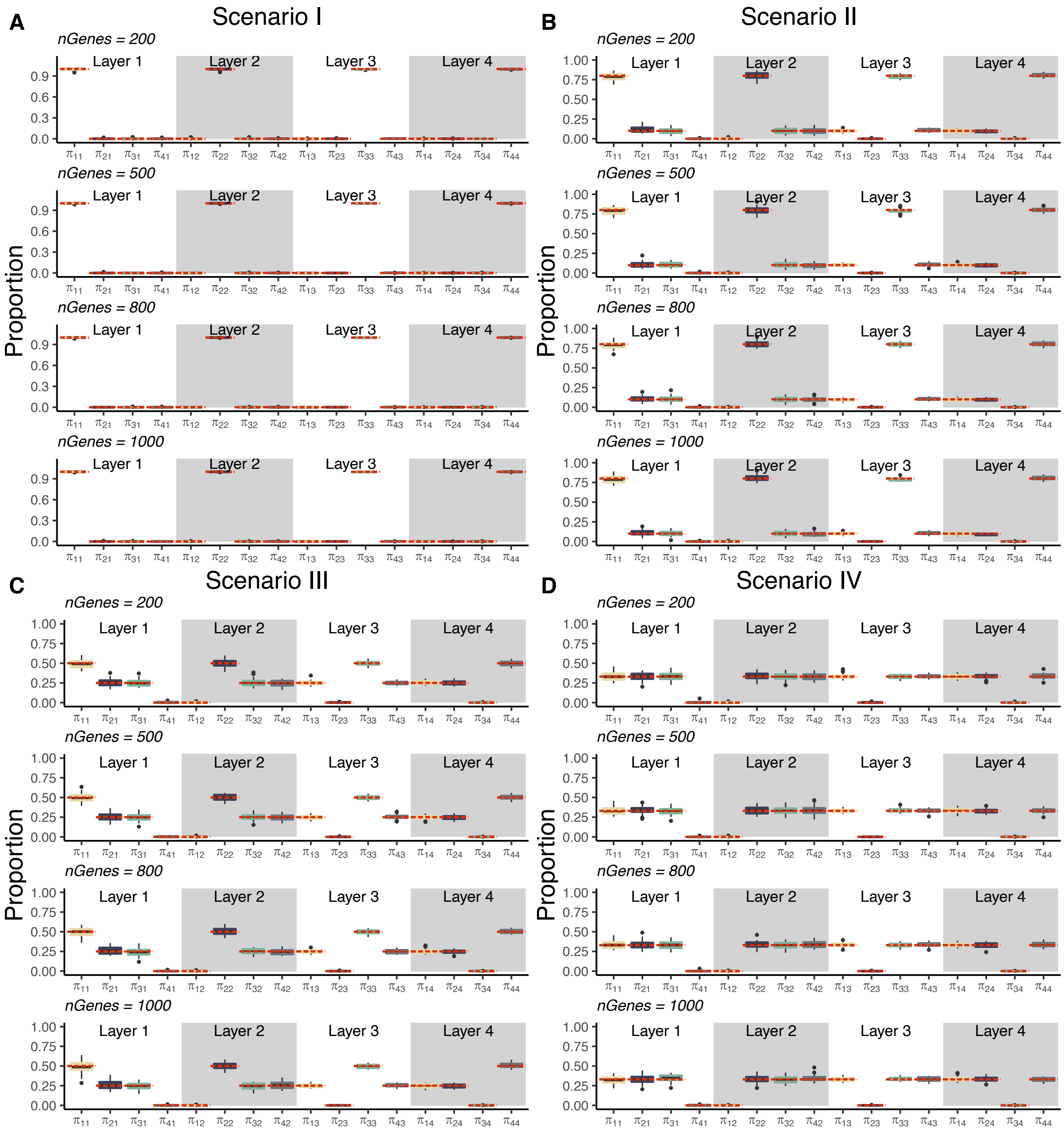

Fig. S11 Performance of BASS for cell type proportion estimation in simulations with a single tissue section. Boxplots show the estimated cell type proportions in each spatial domain across simulation replicates, where π_{cr} indicates the proportion of cell type c in spatial domain r. The red dashed lines indicate the true proportions. Simulations were carried out under (**A**) scenario I, (**B**) scenario II, (**C**) scenario III, or (**D**) scenario IV, with varying number of genes ($nGenes$ = 200, 500, 800 or 1000). The other parameters were set to be $de.facloc = 1.1$ and $de.prob = 0.2$.

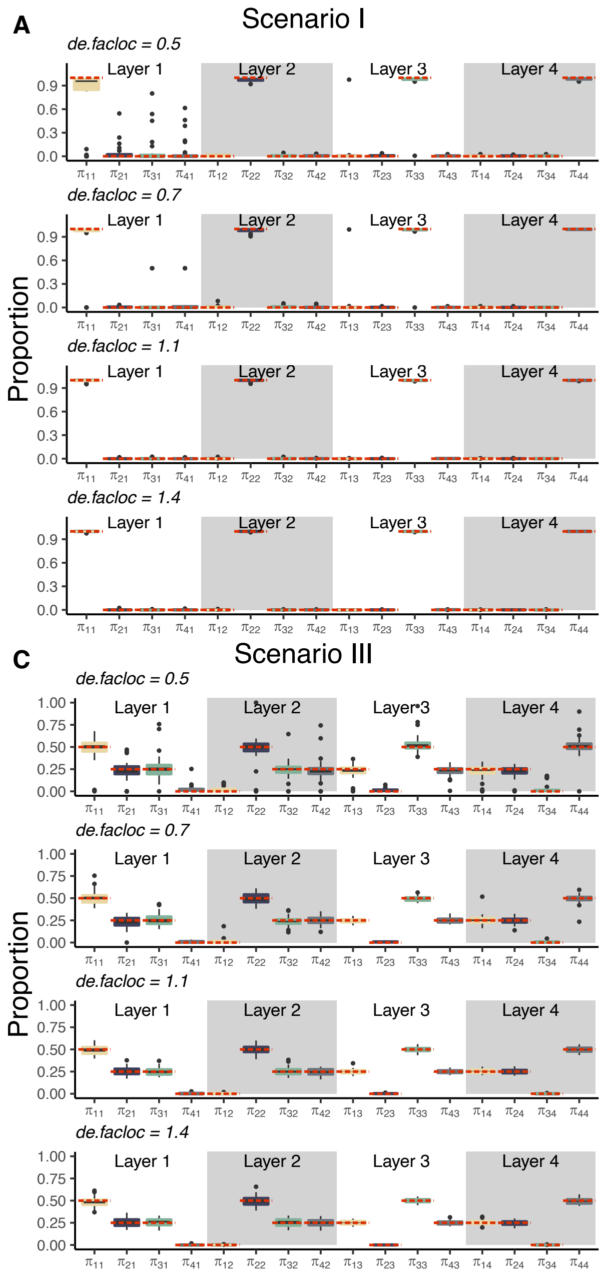

Fig. S12 Performance of BASS for cell type proportion estimation in simulations with a single tissue section. Boxplots show the estimated cell type proportions in each spatial domain across simulation replicates, where π_{cr} indicates the proportion of cell type c in spatial domain r. The red dashed lines indicate the true proportions. Simulations were carried out under (**A**) scenario I, (**B**) scenario II, (**C**) scenario III, or (**D**) scenario IV, with varying DE gene strength ($de.facloc =$ 0.7, 1.1, and 1.4). The other parameters were set to be $nGenes = 200$ and $de.prob = 0.2$.

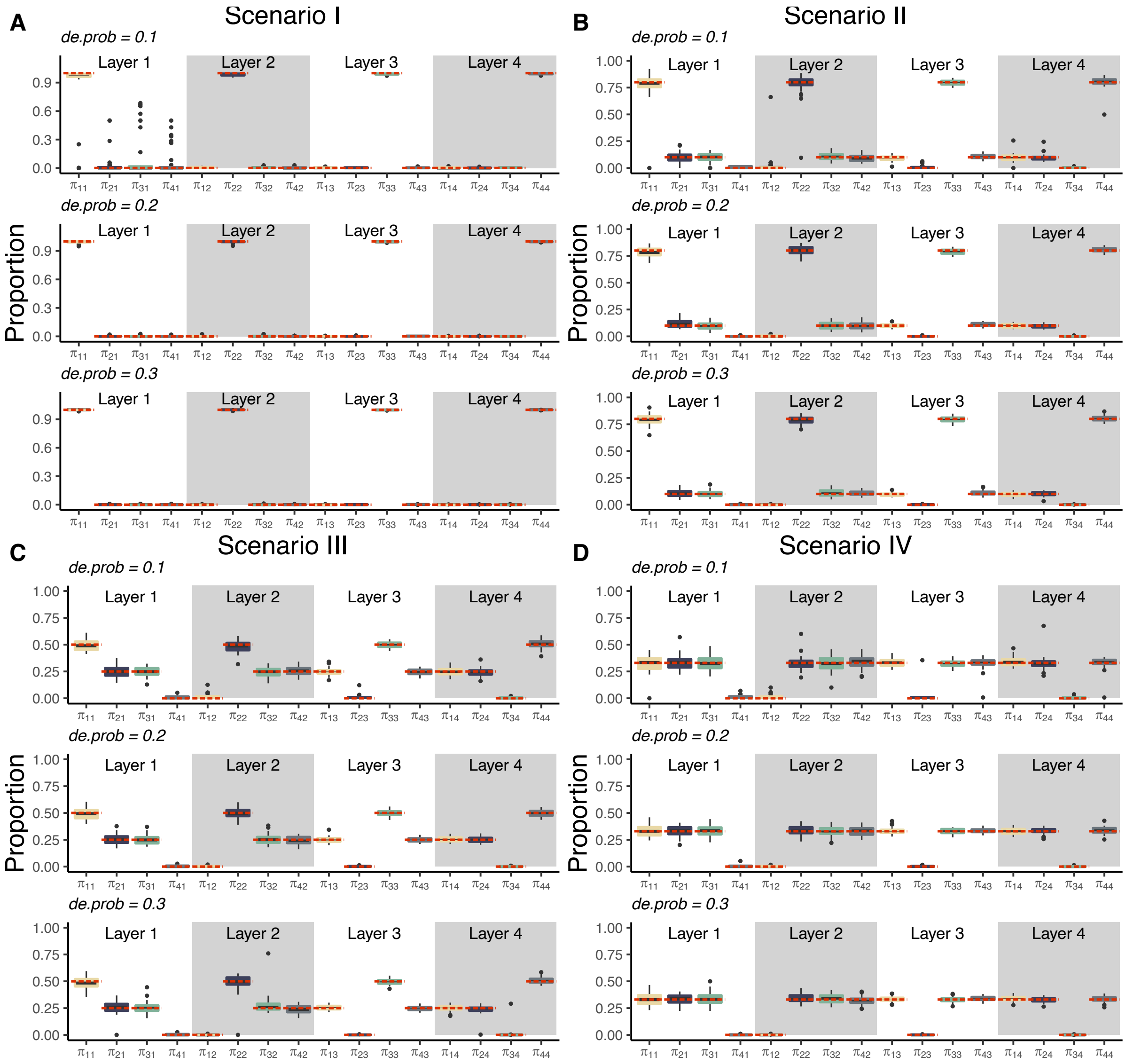

Fig. S13 Performance of BASS for cell type proportion estimation in simulations with a single tissue section. Boxplots show the estimated cell type proportions in each spatial domain across simulation replicates, where π_{cr} indicates the proportion of cell type c in spatial domain r. The red dashed lines indicate the true proportions. Simulations were carried out under (**A**) scenario I, (**B**) scenario II, (**C**) scenario III, or (**D**) scenario IV, with varying proportions of genes that were differentially expressed in each cell type versus the others ($de.prob = 0.1, 0.2,$ and 0.3). The other parameters were set to be $nGenes = 200$ and $de.facloc = 1.1$.

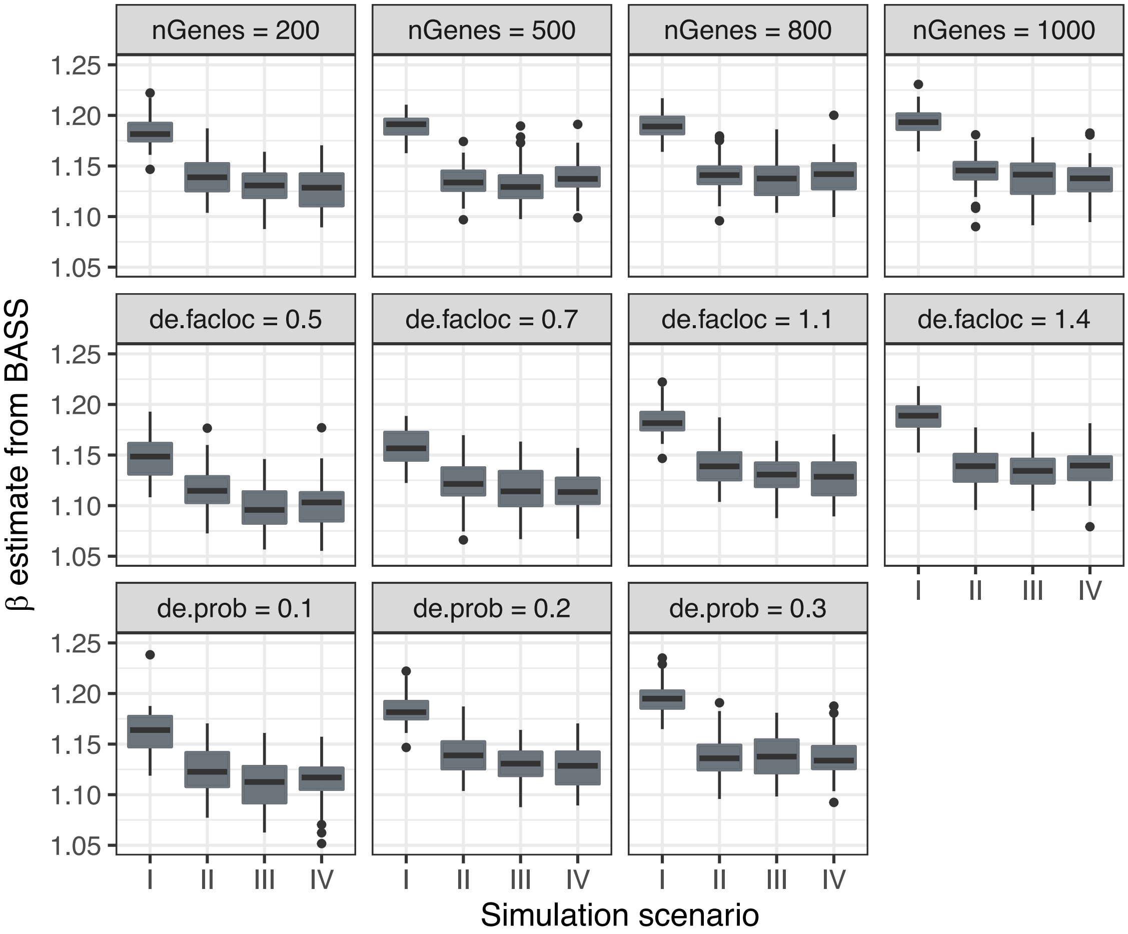

Fig. S14 Estimates of spatial interaction parameter β in the simulation study. Boxplots display the estimates of β from BASS across 50 simulation replicates under simulation scenarios I-IV, with varying number of genes (first row), DE gene strength (second row), and proportion of genes that were differentially expressed in each cell type versus the others (third row). The other two parameters were set to be the baseline ($nGenes = 200$, $de.facloc = 1.1$, and $de.prob = 0.2$) while varying each parameter.

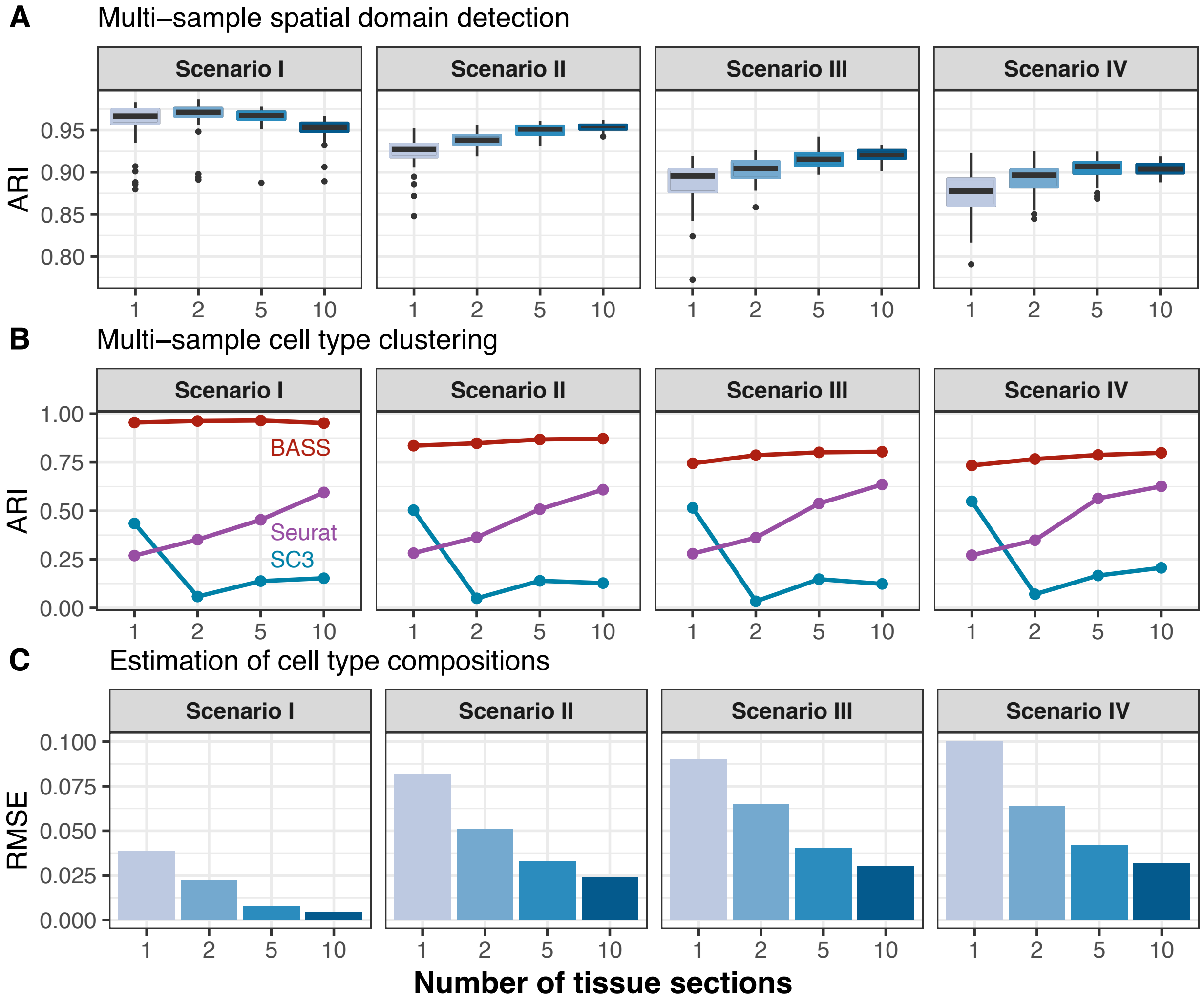

Fig. S15 Comparison of different methods for spatial domain detection and cell type clustering in simulations with multiple tissue sections. (**A**) Boxplots of ARI show the accuracy of BASS for spatial domain detection (y-axis) in the presence of 1, 2, 5 or 10 tissue sections (x-axis). (**B**) Line plots display the median ARI by different methods for cell type clustering across 50 simulation replicates (y-axis) in the presence of 1, 2, 5 or 10 tissue sections (x-axis). Compared methods for cell type clustering include BASS, Seurat and SC3. (**C**) Barplots show the median RMSE between the estimated cell type compositions and the true compositions across 50 simulation replicates (y-axis) in the presence of 1, 2, 5 or 10 tissue sections (x-axis). Simulations were conducted under scenarios I-IV, with the simulation parameters set to be $nGenes = 200$, $de.prob = 0.2$, and $de.facloc = 0.7$.

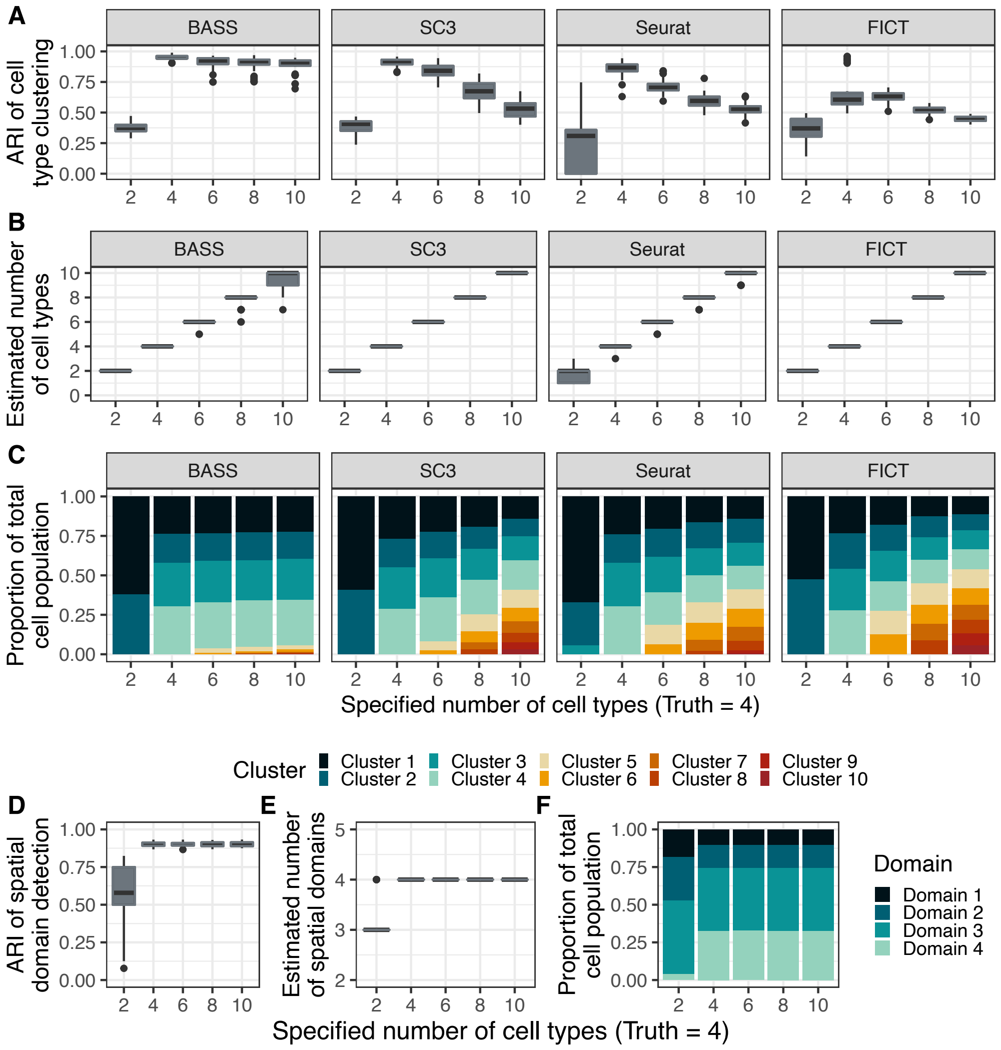

Fig. S16 Evaluation of the influence of a mis-specified number of cell types on the method performance. (**A**-**C**) Influence of the specified number of cell types on the performance of cell type clustering. Compared methods included BASS, SC3, Seurat, and FICT. Accuracy of cell type clustering was evaluated based on three criteria that include (**A**) the overall agreement between the estimated labels and true labels measured by ARI; (**B**) the number of estimated cell type clusters; and (**C**) the average proportion of cells in each cluster. There, the top 4 clusters corresponded to the 4 cell types while the remaining clusters were arranged from the largest to the smallest. (**D**-**F**) Influence of the specified number of cell types on the performance of spatial domain detection in BASS, evaluated based on the same criteria above. The number of cell types was set to be either $2, 4, 6, 8$ or 10 while the truth was 4. Simulations were conducted under the baseline setting of the scenario III (i.e., $nGenes = 200, de.prob = 0.2,$ and $de.facloc = 1.1$).

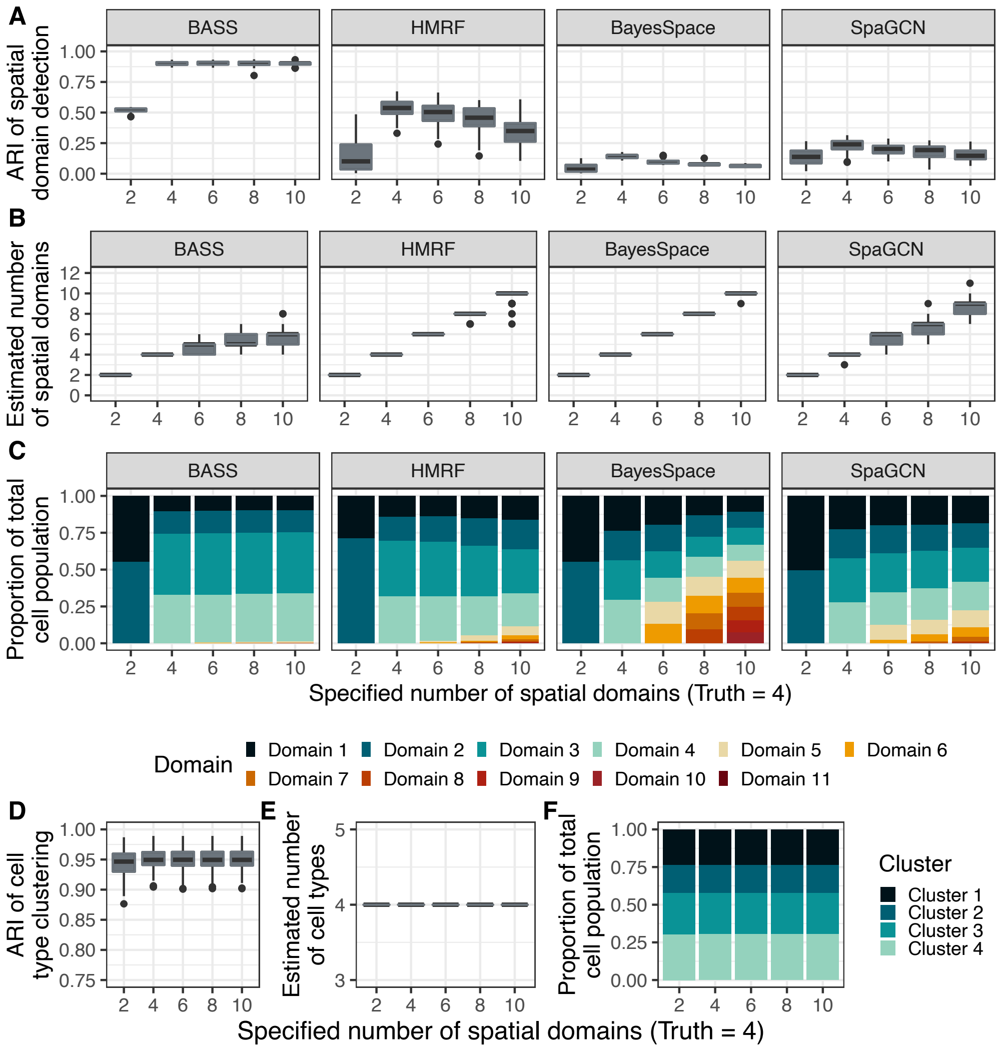

Fig. S17 Evaluation of the influence of a mis-specified number of spatial domains on the method performance. (**A-C**) Influence of the specified number of spatial domains on the performance of spatial domain detection. Compared methods included BASS, the oracle version of HMRF, BayesSpace, and SpaGCN. Accuracy of spatial domain detection was evaluated based on three criteria that include (**A**) the overall agreement between the estimated labels and true labels measured by ARI; (**B**) the number of estimated spatial domain clusters; and (**C**) the average proportion of cells in each cluster. There, the top 4 clusters corresponded to the 4 spatial domains while the remaining clusters were arranged from the largest to the smallest. (**D-F**) Influence of the specified number of spatial domains on the performance of cell type clustering in BASS, evaluated based on the same criteria above. The number of spatial domains was set to be either 2, 4, 6, 8 or 10 while the truth was 4. Simulations were conducted under the baseline setting of the scenario III (i.e., $nGenes = 200, de.prob = 0.2,$ and $de.facloc = 1.1$).

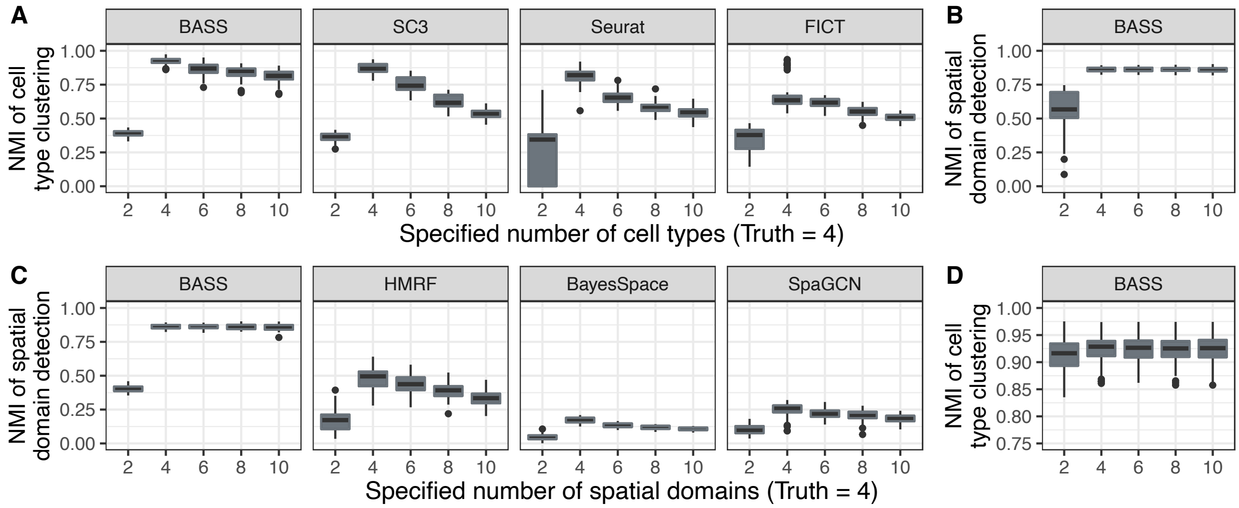

Fig. S18 Evaluation of the influence of a mis-specified number of cell types or spatial domains on the method performance. Accuracy of cell type clustering or spatial domain detection was evaluated by the normalized mutual information (NMI). (**A**) Influence of the specified number of cell types on the performance of cell type clustering. Compared methods included BASS, SC3, Seurat, and FICT. (**B**) Influence of the specified number of cell types on the performance of spatial domain detection in BASS. (**C**) Influence of the specified number of spatial domains on the performance of spatial domain detection. Compared methods included BASS, the oracle version of HMRF, BayesSpace, and SpaGCN. (**D**) Influence of the specified number of spatial domains on the performance of cell type clustering in BASS.

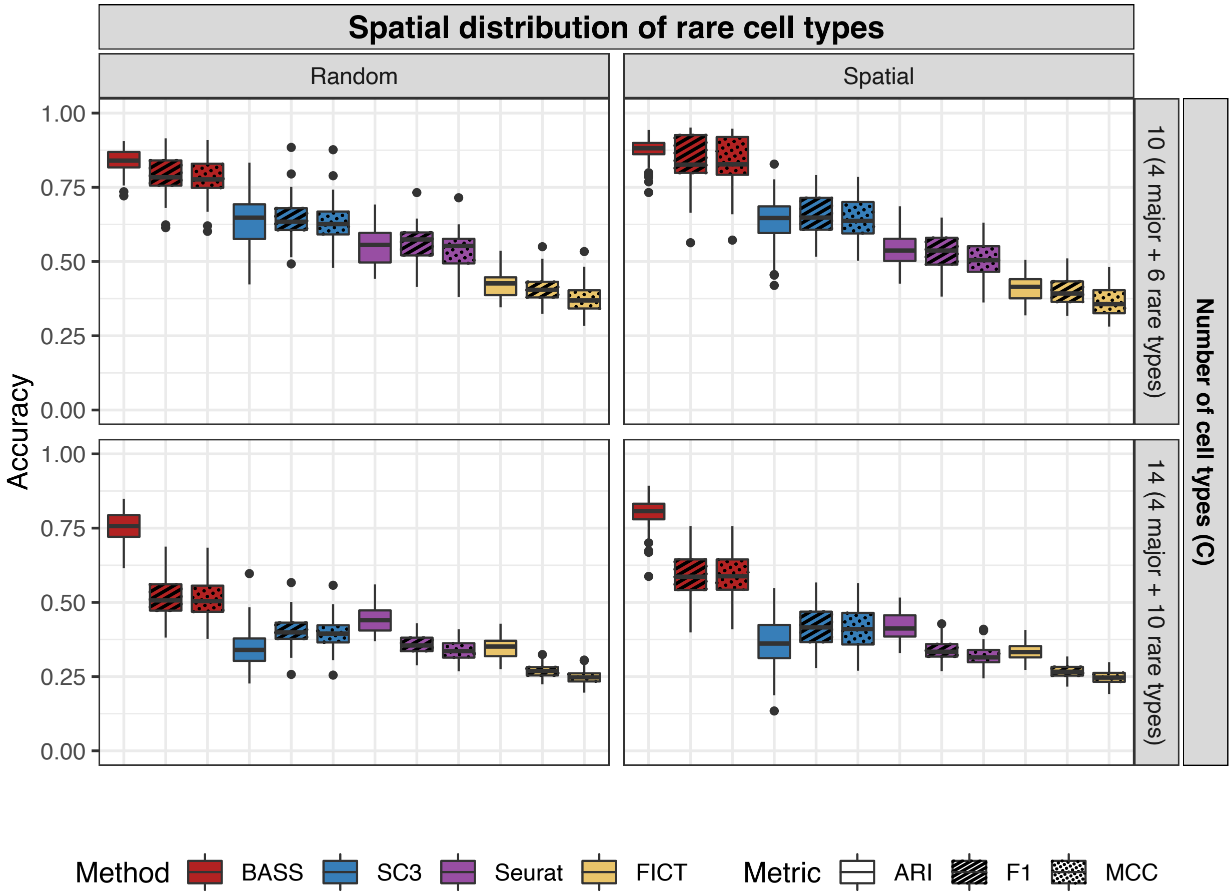

Fig. S19 Evaluation of the performance of different cell type clustering methods in the presence of rare cell types. Boxplots display three metrics including the ARI, F_1 score, and Matthews correlation coefficient (MCC) for evaluating the performance of different methods on cell type clustering in the presence of either 6 (first row) or 10 (second row) rare cell types and 4 major cell types. The rare cell types in total comprised 30% of cells, with each rare cell type consisted of 5% or 3% of the total cell population. The rare cell types exhibit either a random distribution pattern (first column) or a domain-specific pattern (second column) while the composition of major cell types in different spatial domains was set to be the same as that in the scenario III, where each spatial domain contained three major cell types with a 2:1:1 ratio. Compared methods for cell type clustering include BASS, Seurat, SC3, and FICT. All the other parameters were set to be the baseline, where $nGenes = 200$, $de.facloc = 1.1$, and $de.prob = 0.2$.

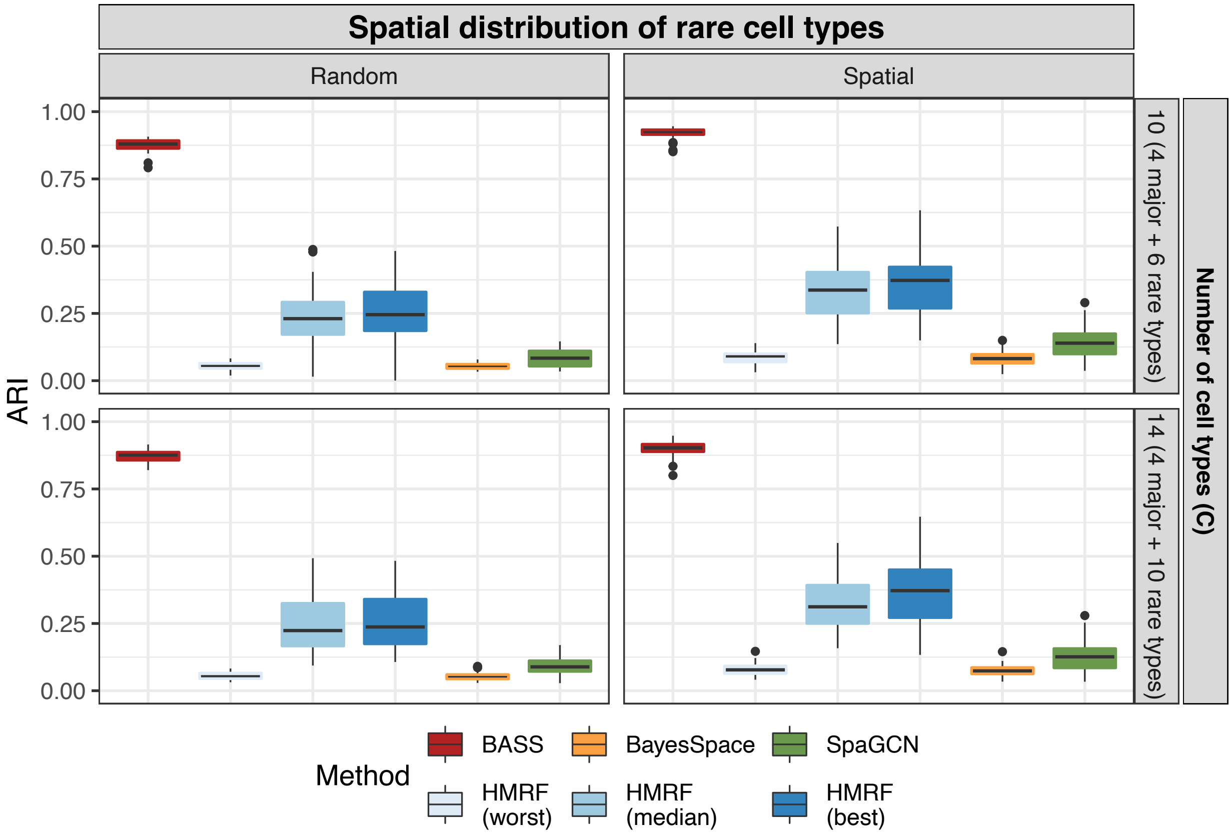

Fig. S20 Evaluation of the performance of different spatial domain detection methods in the presence of rare cell types. Boxplots display three metrics including the ARI, F_1 score, and Matthews correlation coefficient (MCC) for evaluating the performance of different methods on spatial domain detection in the presence of either 6 (first row) or 10 (second row) rare cell types and 4 major cell types. The rare cell types in total comprised 30% of cells, with each rare cell type consisted of 5% or 3% of the total cell population. The rare cell types exhibit either a random distribution pattern (first column) or a domain-specific pattern (second column) while the composition of major cell types in different spatial domains was set to be the same as that in the scenario III, where each spatial domain contained three major cell types with a 2:1:1 ratio. Compared methods for spatial domain detection included BASS, HMRF, BayesSpace, and SpaGCN. For HMRF, a list of the spatial parameter β ranging from 0 to 50 at increments of 2 were examined and the three β values that corresponded to the worst, median and best performance are displayed. All the other parameters were set to be the baseline, where $nGenes = 200$, $de.facloc = 1.1$, and $de.prob = 0.2$.

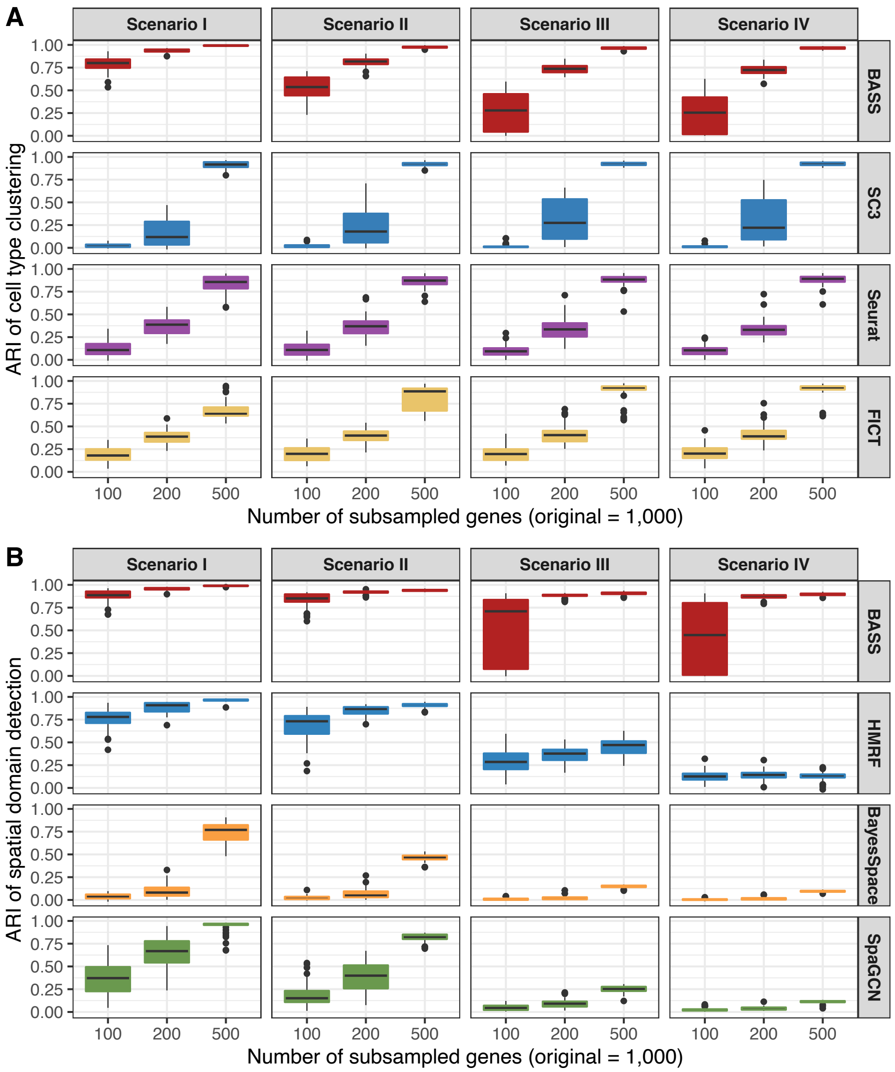

Fig. S21 Evaluation of the influence of randomly excluding genes from the gene expression matrix on the performance of different methods. Boxplots of ARI show the accuracy of different methods for (**A**) cell type clustering or (**B**) spatial domain detection after retaining only a subset of 100, 200, or 500 genes in the gene expression matrix while the original number of genes was set to be 1000. The other parameters were set to be the baseline, where $de.facloc = 1.1$ and $de.prob = 0.2$.

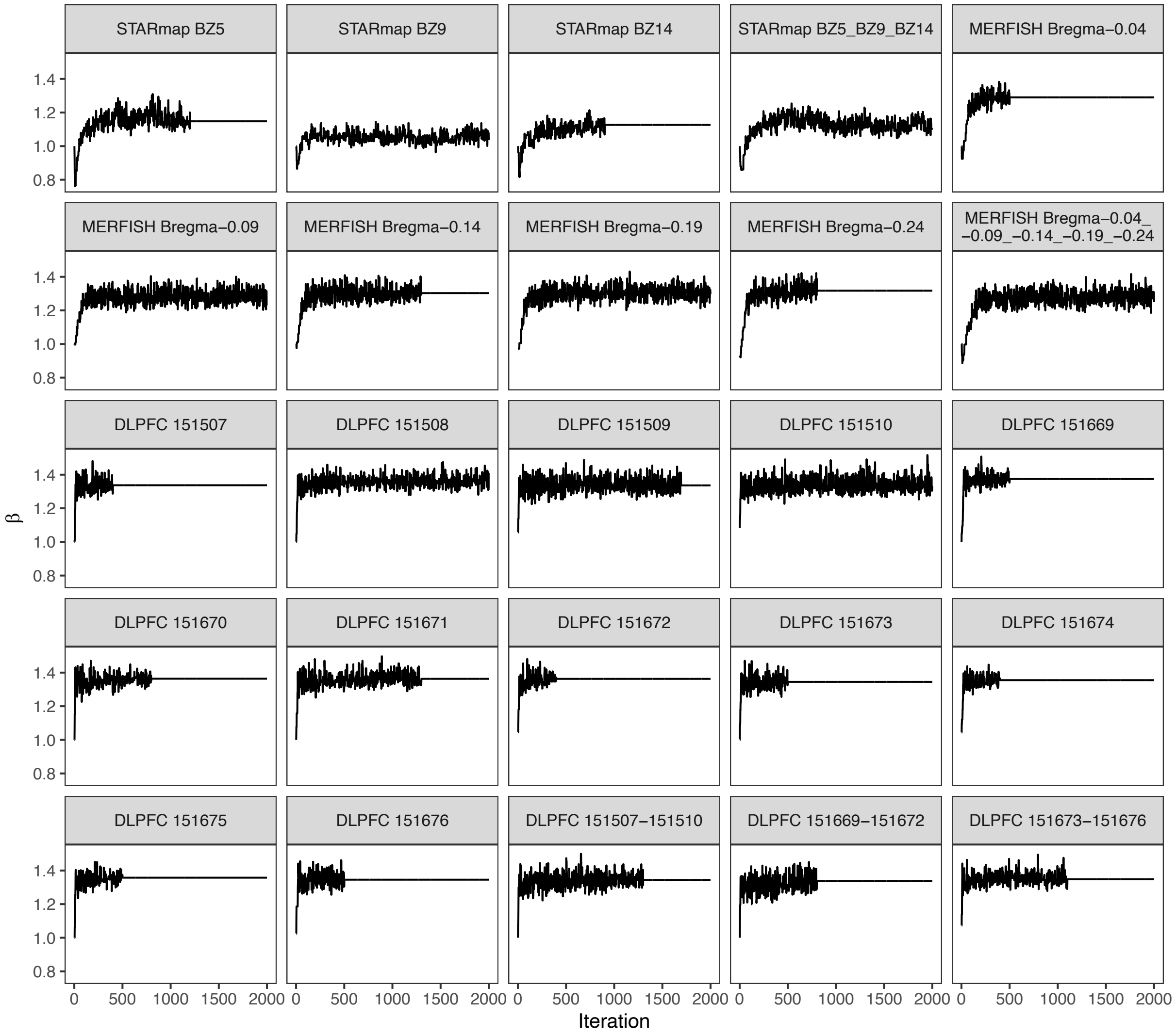

Fig. S22 Trace plots of the spatial interaction parameter $\boldsymbol{\beta}$ in all the real data applications. The BASS software implements a stopping rule to automatically determine the number of MCMC samples required for model fitting. BASS calculates the mean of the sampled β in every 100 iterations and stops sampling if the difference in the consecutive two means is below a certain threshold (set to be 0.001 by default). Such stopping rule can help improve the computation efficiency and works well in all the analyzed real datasets.

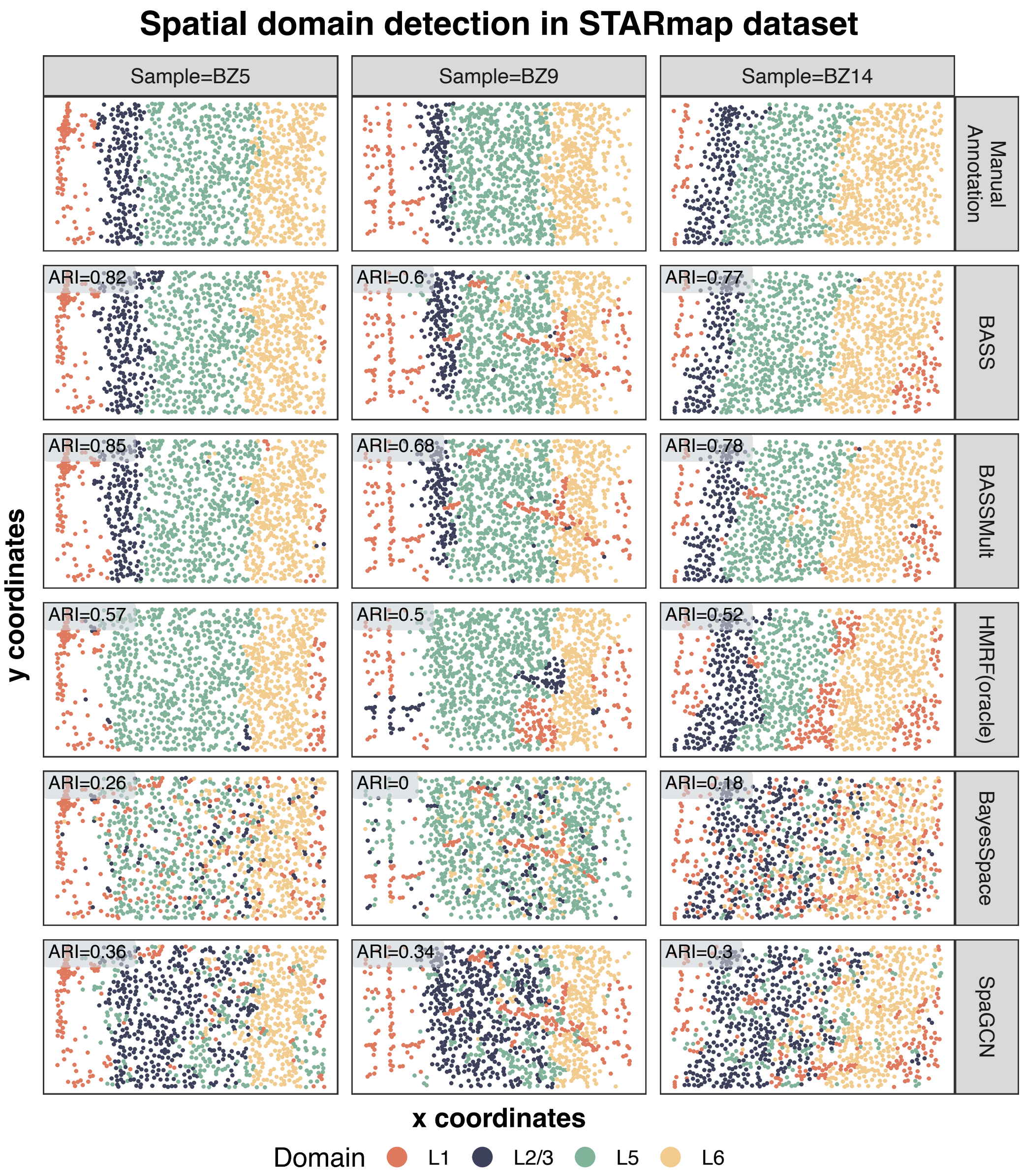

Fig. S23 Detecting spatial domains in the mouse medial prefrontal cortex data by STARmap. (**First row**) Cells from three tissue sections (BZ5, BZ9, and BZ14) were manually annotated to four distinct cortical structures that included L1, L2/3, L5, and L6 based on the spatial expression of marker genes and the histology diagram of the mouse brain from the Allen's brain atlas. (**Second to fifth row**) The identified spatial domains on the three tissue sections are shown for the single-sample analysis of BASS, multi-sample analysis of BASS, oracle version of HMRF, BayesSpace, and SpaGCN. ARIs in the grey boxes show the accuracy of different methods for spatial domain detection.

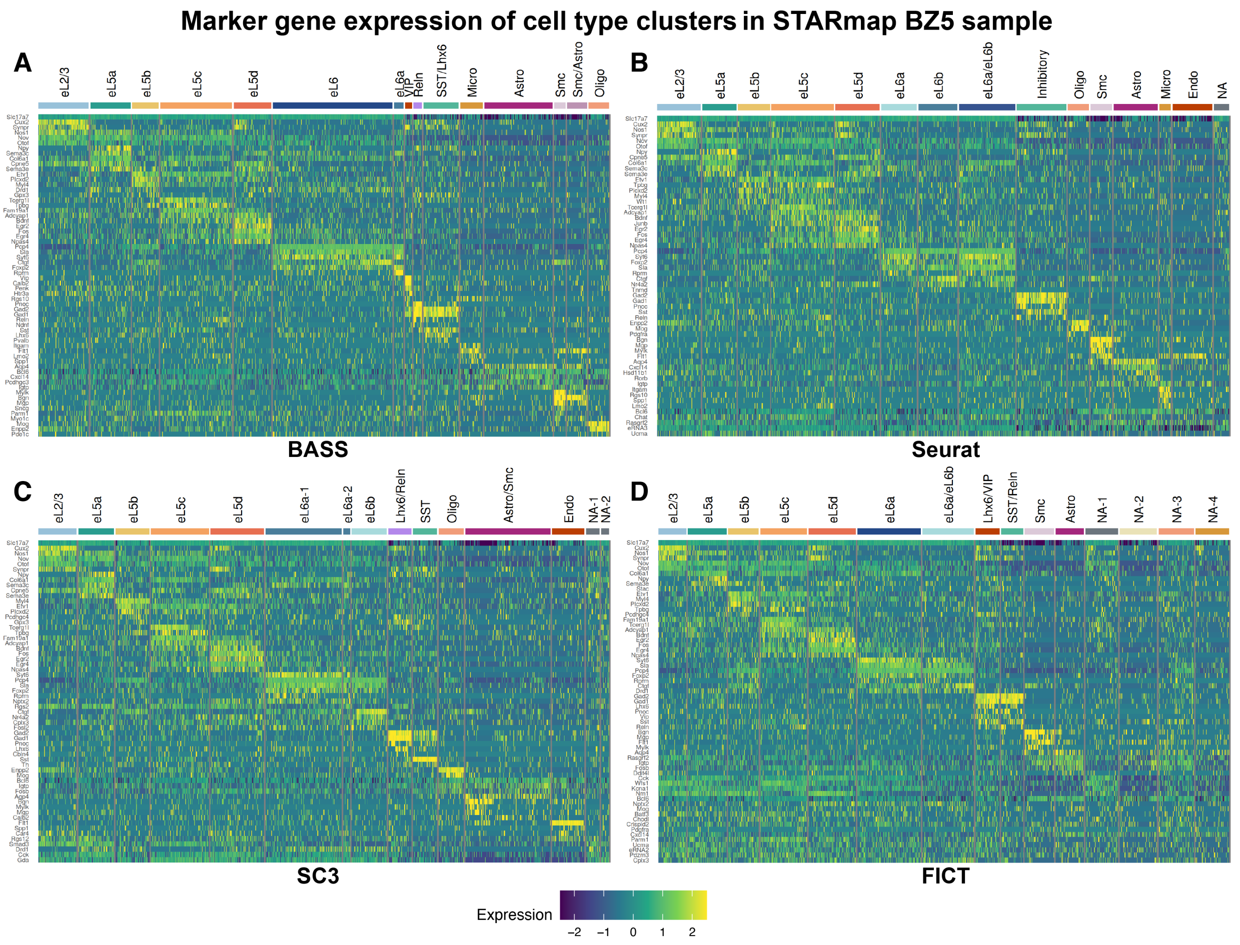

Fig. S24 Gene expression heatmaps of cell type clusters identified by different methods on tissue section BZ5 from the STARmap dataset. Each gene was centered and standardized across all the cells. For each cell type cluster, gene expression of the top five (or all if less than five) differentially expressed (DE) genes are displayed, where DE genes were identified using the Wilcoxon rank-sum test contrasting each cluster of cells against all the remaining cells. Cell type clusters were annotated with specific cell types by comparing the identified DE genes with previously known cell type marker genes. Compared methods include (**A**) BASS, (**B**) Seurat, (**C**) SC3, and (**D**) FICT.

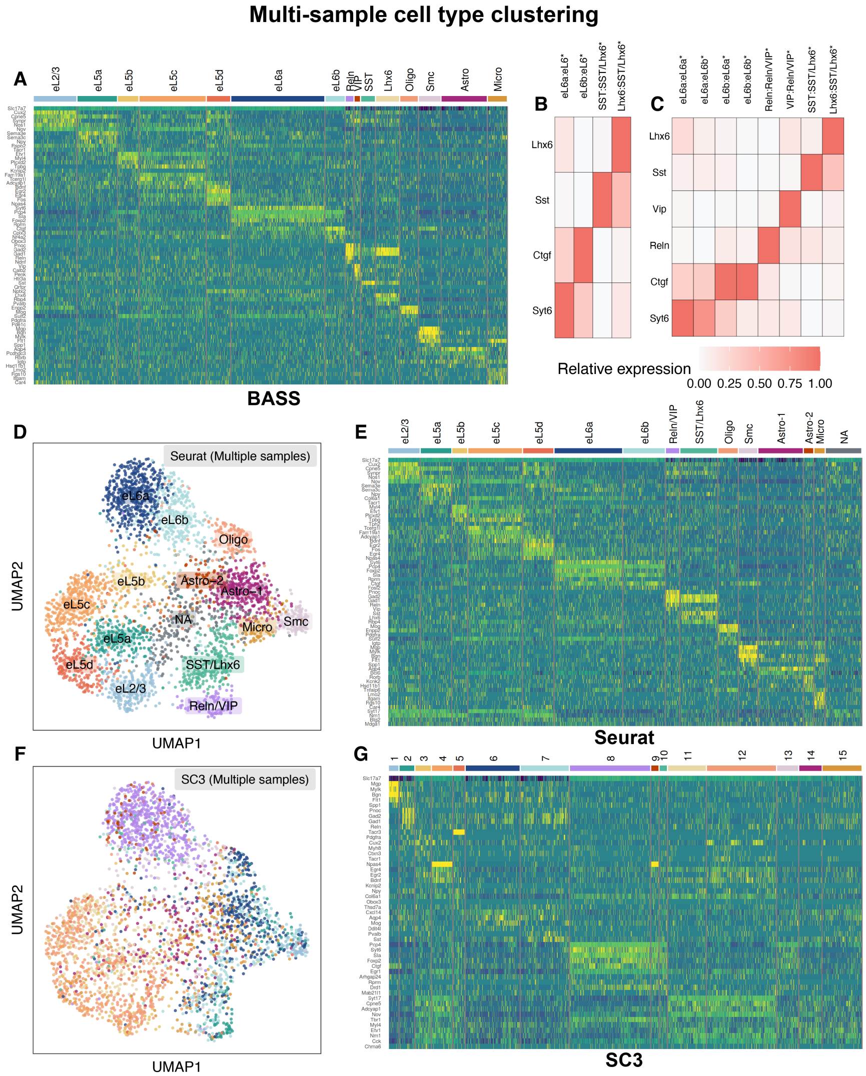

Fig. S25 Multi-sample integrative analysis of cell types in the STARmap dataset. (**A, E, G**) Gene expression heatmaps of cell type clusters identified by (**A**) BASS, (**E**) Seurat, or (**G**) SC3 jointly analyzing three tissue sections (BZ5, BZ9, and BZ14) from the STARmap dataset. (**B-C**) Relative expression of marker genes for cells that have cell types assigned by the multi-sample integrative analysis of BASS, followed by a colon, and followed by the cell types assigned by (**B**) the single-sample analysis of BASS or (**C**) multi-sample analysis of Seurat. The relative expression in (**B**) was calculated with cells from a single tissue section BZ5 while the relative expression in (**C**) was calculated with cells from all three tissue sections. The marker genes were selected for cell types: eL6a (*Syt6*), eL6b (*Ctgf*), SST (*Sst*), Lhx6 (*Lhx6*), Reln (*Reln*), and VIP (*Vip*). (**D, F**) UMAP visualization of cell type clustering results on three tissue sections by the multi-sample analysis of (**D**) Seurat or (**F**) SC3.

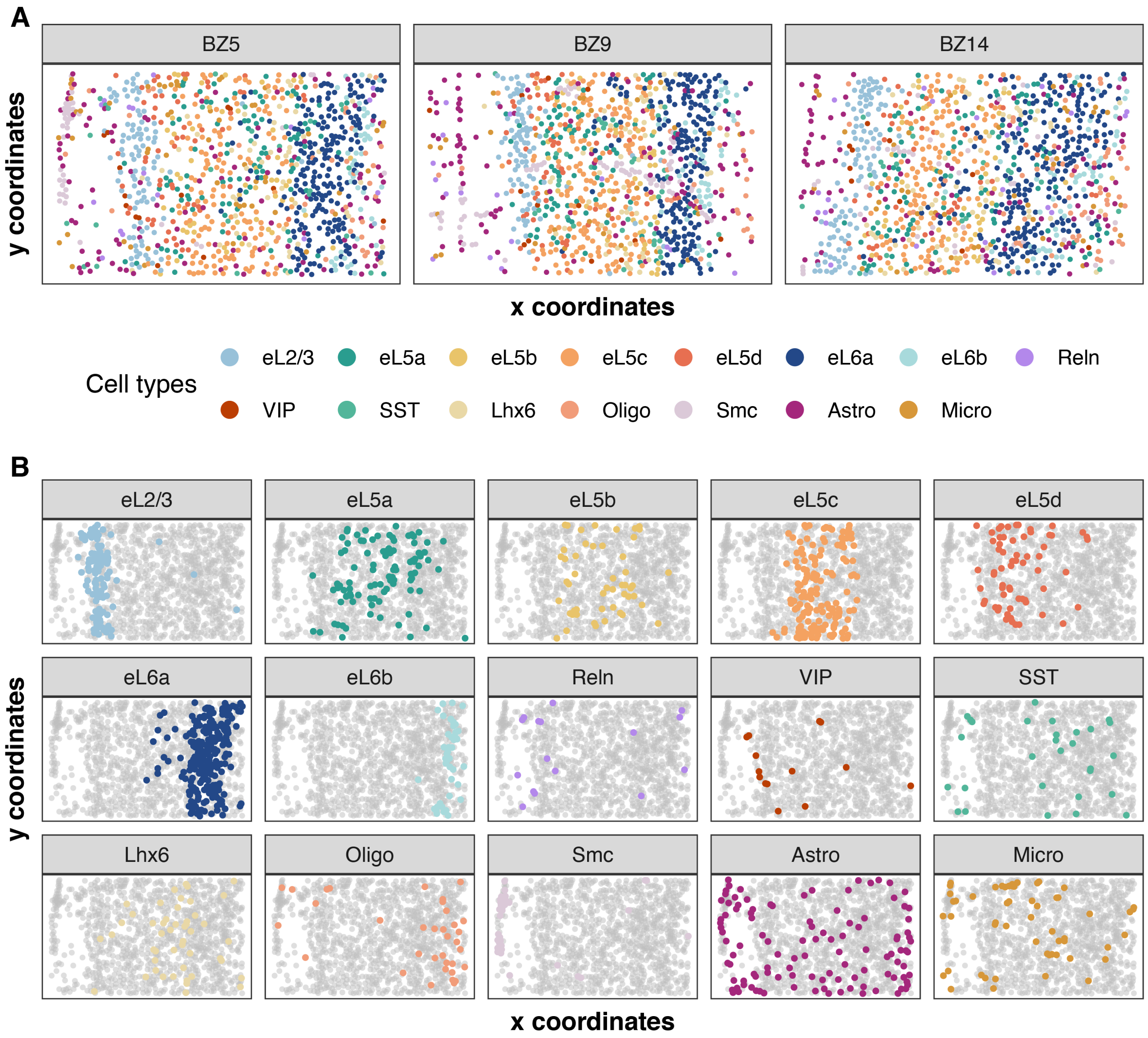

Fig. S26 Spatial distribution of cell types obtained with the multi-sample analysis of BASS on three tissue sections (BZ5, BZ9, and BZ14) from the STARmap dataset. (**A**) Spatial distribution of cell types on the three tissue sections. (**B**) Spatial distribution of each cell type on the tissue section BZ5.

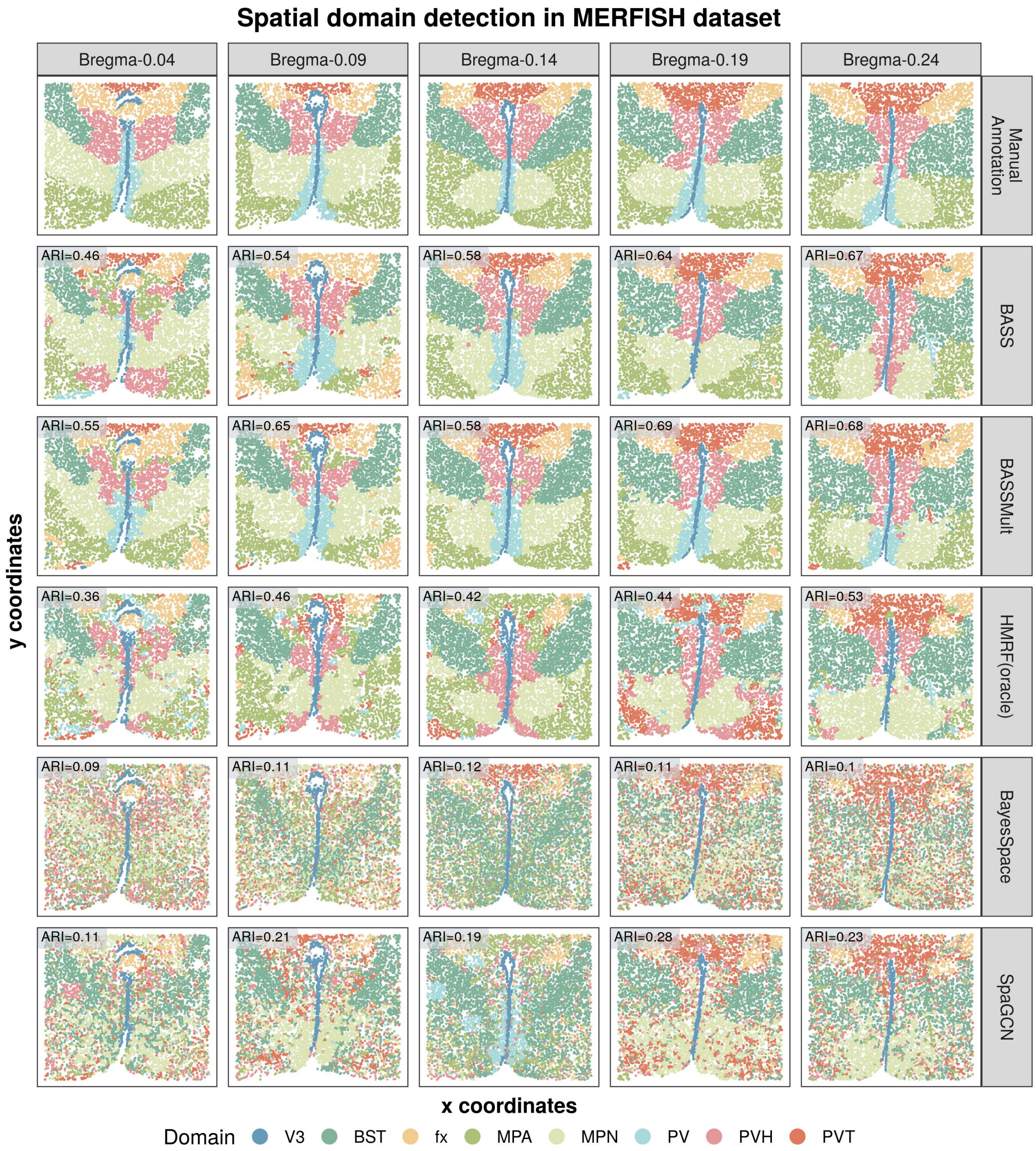

Fig. S27 Detecting spatial domains in the mouse hypothalamus data by MERFISH. (**First row**) Cells from five tissue sections (Bregma-0.04, -0.09, -0.14, -0.19, and -0.24) were manually annotated to eight distinct structures that included V3, BST, fx, MPA, MPN, PV, PVH, and PVT based on the spatial expression of marker genes and the histology diagram of the mouse brain from the Allen's brain atlas. (**Second to fifth row**) The identified spatial domains on the five tissue sections are shown for the single-sample analysis of BASS, multi-sample analysis of BASS, oracle version of HMRF, BayesSpace, and SpaGCN. ARIs in the grey boxes show the accuracy of different methods for spatial domain detection.

Marker gene expression of cell type clusters in MERFISH Bregma-0.14 sample

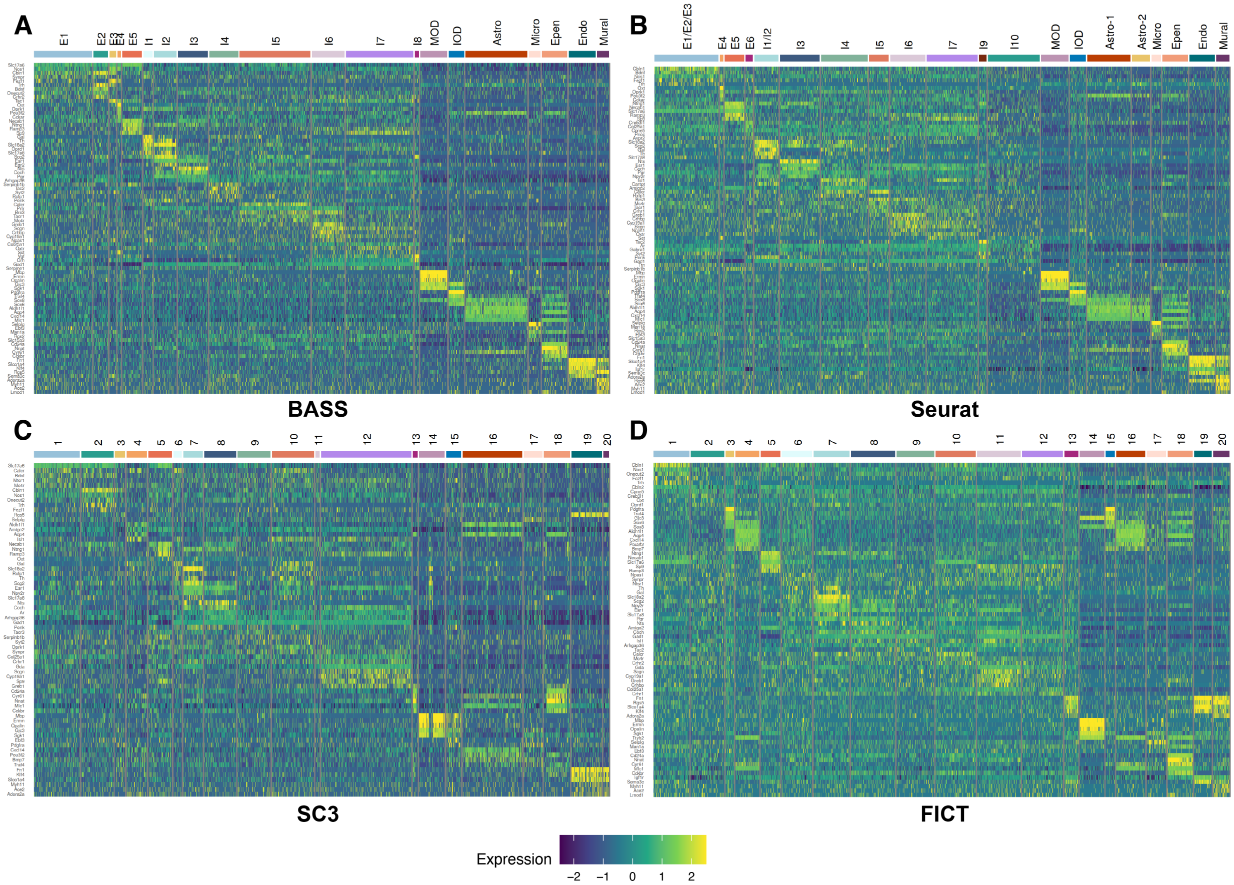

Fig. S28 Gene expression heatmaps of cell type clusters identified by different methods on tissue section Bregma-0.14 from the MERFISH dataset. Each gene was centered and standardized across all the cells. For each cell type cluster, gene expression of the top five (or all if less than five) differentially expressed (DE) genes are displayed, where DE genes were identified using the Wilcoxon rank-sum test contrasting each cluster of cells against all the remaining cells. Cell type clusters were annotated with specific cell types by comparing the identified DE genes with previously known cell type marker genes. Compared methods include (**A**) BASS, (**B**) Seurat, (**C**) SC3, and (**D**) FICT.

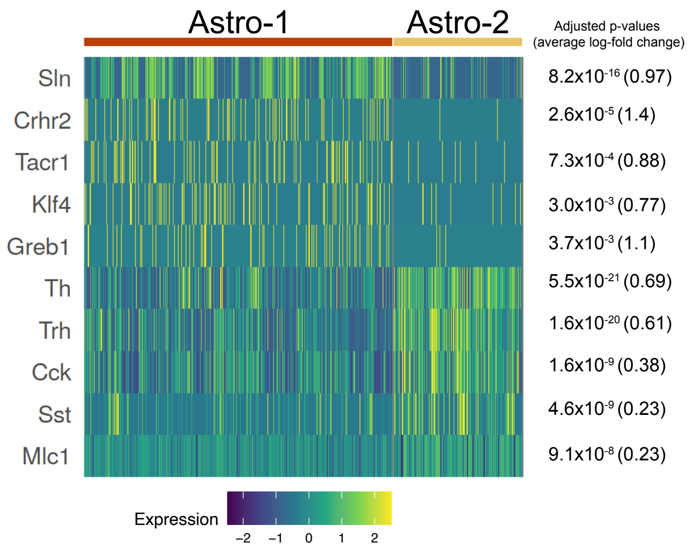

Fig. S29 Gene expression heatmap of Astro-1 and Astro-2 clusters identified by Seurat on tissue section Bregma-0.14 from the MERFISH dataset. Each gene was centered and standardized across all cells in these two clusters. For each cluster, gene expression of the top five up-regulated differentially expressed (DE) genes are displayed, where DE genes were identified using the Wilcoxon rank-sum test contrasting cells in one cluster against the other cluster.

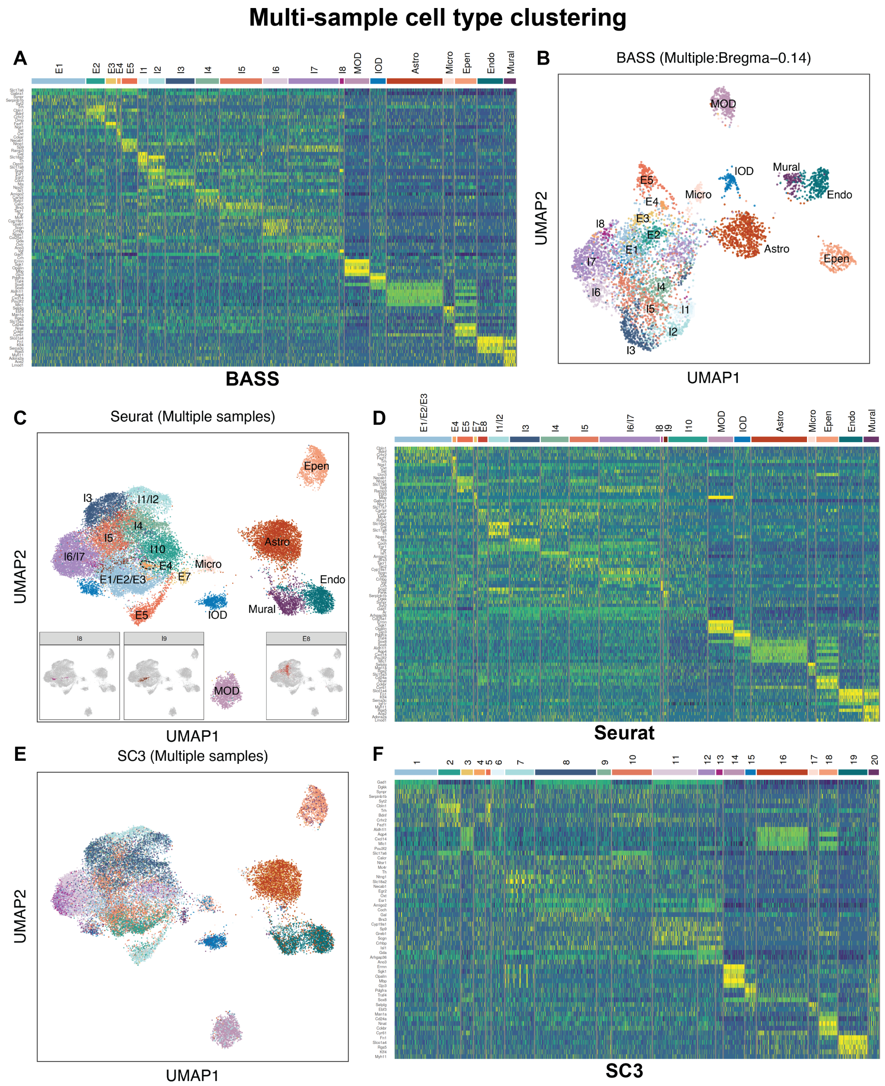

Fig. S30 Multi-sample integrative analysis of cell types in the MERFISH dataset. (**A, D, F**) Gene expression heatmaps of cell type clusters identified by (**A**) BASS, (**D**) Seurat, or (**F**) SC3 jointly analyzing five tissue sections (Bregma-0.04, -0.09, -0.14, -0.19, and -0.24) from the MERFISH dataset. (**B**) UMAP visualization of cell type clustering results on the tissue section Bregma-0.14 by the multi-sample version of BASS. (**C, E**) UMAP visualization of cell type clustering results on five tissue sections by the multi-sample version of (**C**) Seurat or (**E**) SC3.

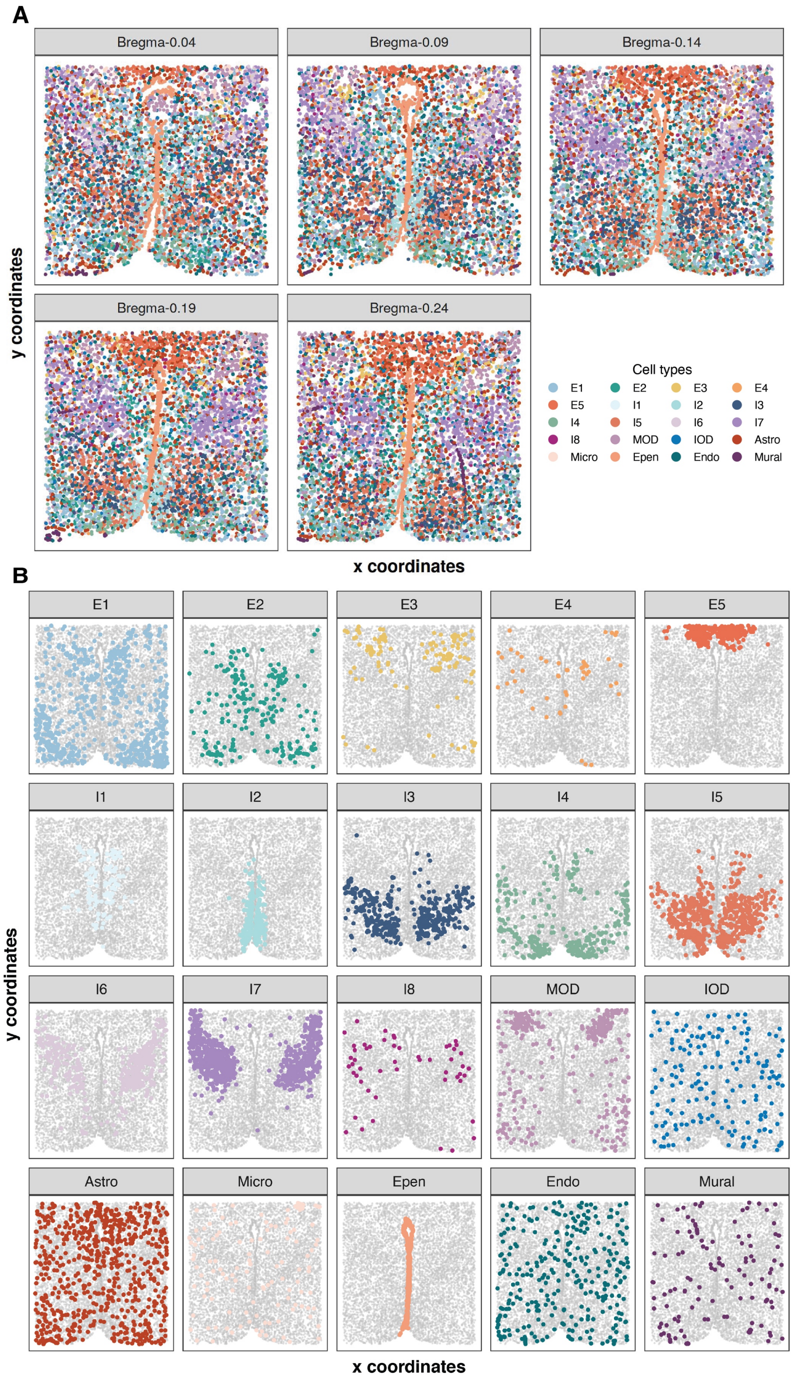

Fig. S31 Spatial distribution of cell types obtained with the multi-sample analysis of BASS on five tissue sections (Bregma-0.04, -0.09, -0.14, -0.19, and -0.24) from the MERFISH dataset. (**A**) Spatial distribution of cell types on the five tissue sections. (**B**) Spatial distribution of each cell type on the tissue section Bregma-0.14.

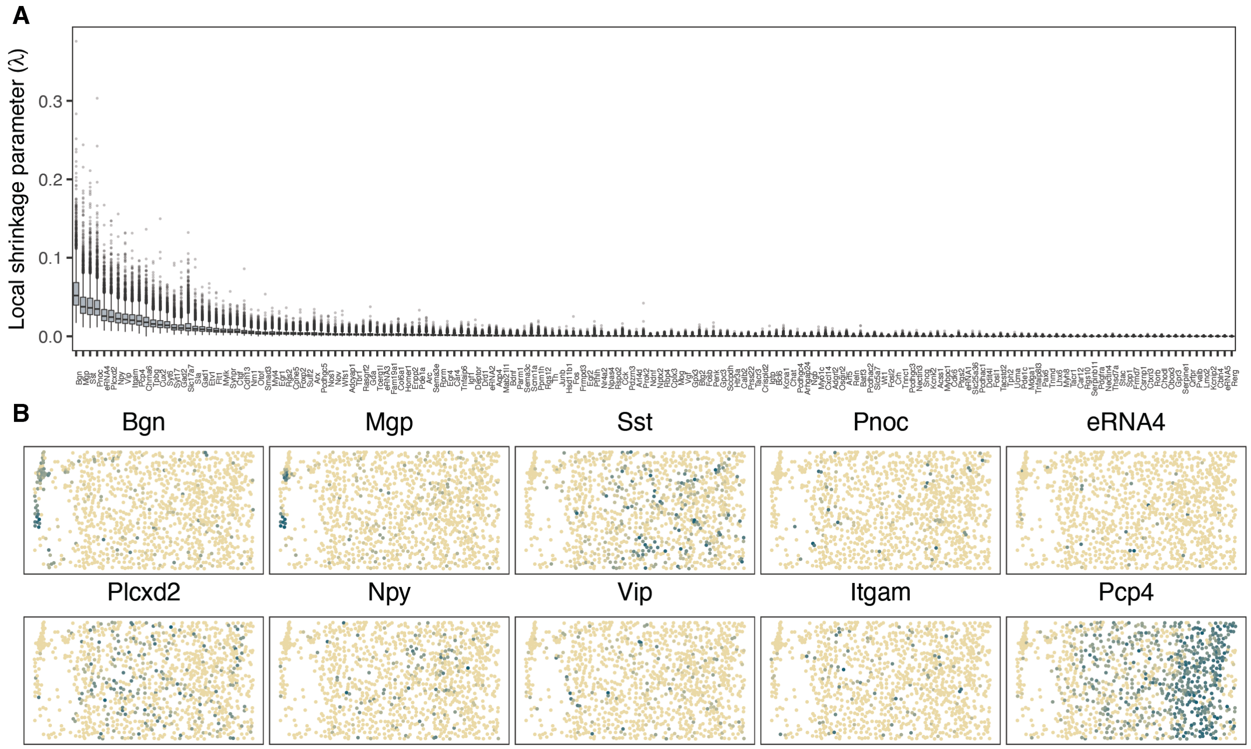

Fig. S32 Selection of cluster informative genes with local shrinkage parameters. BASS was directly applied to the normalized and log-transformed gene expression matrix instead of the low-dimentional embeddings (i.e., PCs). The analysis was carried out using the tissue section BZ5 from the STARmap dataset. (**A**) Boxplots of the local shrinkage parameter (λ_j) across the posterior samples for each gene. (**B**) Relative expression of the top 10 genes with the highest local shrinkage parameter estimates.

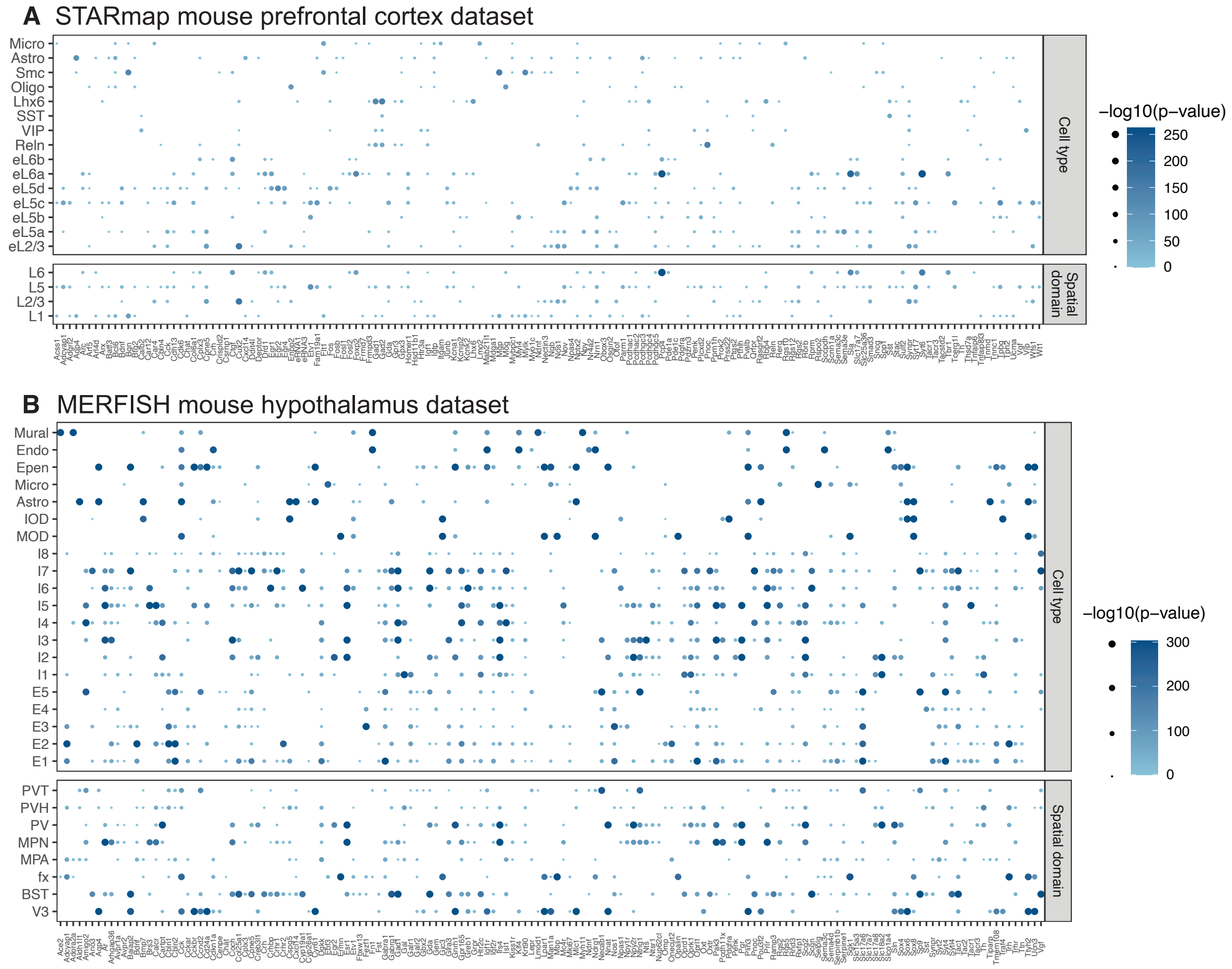

Fig. S33 Dot plots of -log$_{10}$p-values from the differential expression analyses. Differential expression (DE) analyses were carried out in the (**A**) STARmap or (**B**) MERFISH datasets. Genes were tested for differential expression between each cell type cluster/spatial domain and all the remaining clusters/domains. The cell type and spatial domain labels were obtained from the multi-sample integrative analyses of BASS.

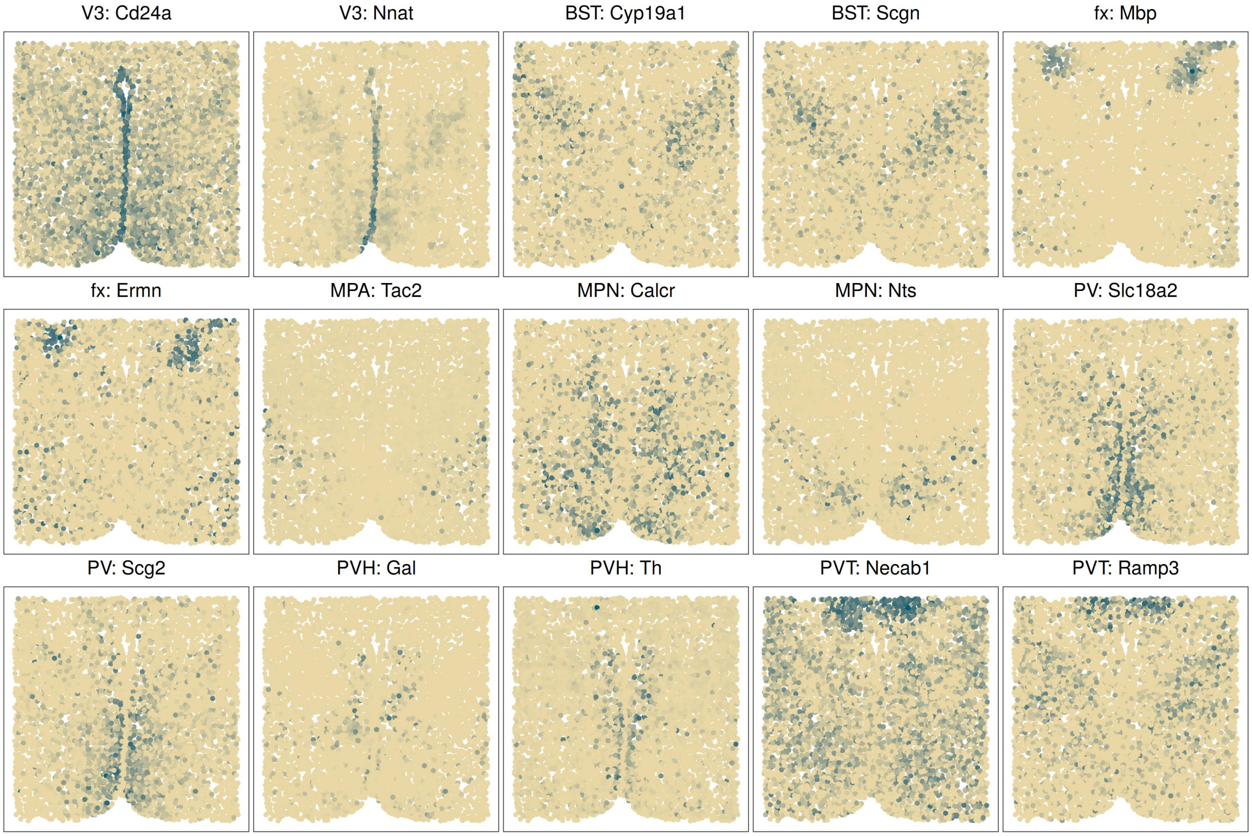

Fig. S34 Visualization of top marker genes for each spatial domain in the mouse hypothalamus. Differentially expression analyses were carried out in the MERFISH dataset for identifying marker genes of each of the eight spatial domains in the mouse hypothalamus based on the spatial domain estimates from BASS. Each panel shows the relative gene-expression levels, with the gene name and the associated domain name displayed on top.

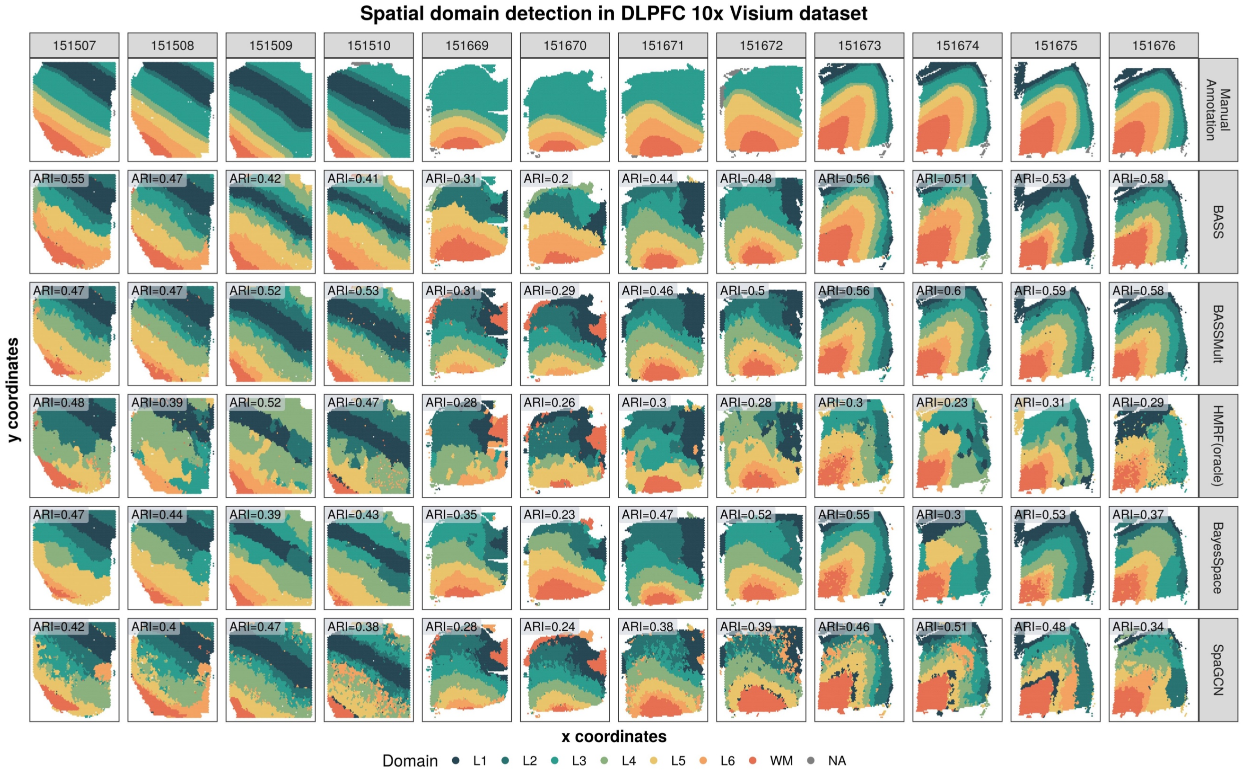

Fig. S35 Detecting spatial domains in the DLPFC data by 10x Visium. (**First row**) Manually annotated labels of seven laminar clusters that included six cortical layers from L1 to L6 and the white matter (WM) by the original study. (**Second to fifth row**) The identified spatial domains on the three tissue sections are shown for the single-sample analysis of BASS, multi-sample analysis of BASS, oracle version of HMRF, BayesSpace, and SpaGCN. ARIs in the grey boxes show the accuracy of different methods for spatial domain detection.

Data	Sample	N	CPU time (miniutes)					Memory usage (GB)				
			BASS	HMRF	BayesSpace	SpaGCN	FICT	BASS	HMRF	BayesSpace	SpaGCN	FICT
STARmap	BZ5	1,049	2	1	9	0.2	4	0.3	0.06	2	0.002	0.1
	BZ9	1,053	2	1	15	0.3	4	0.3	0.06	2	0.002	0.1
	BZ14	1,088	1	1	15	0.3	3	0.3	0.06	3	0.002	0.1
	BZ5+BZ9+BZ14	3,190	6	-	-	-	-	1.2	-	-	-	-
MERFISH	Bregma-0.04	5,488	7	17	65	2	8	2	0.2	12	0.003	0.1
	Bregma-0.09	5,557	15	20	73	4	14	2	0.2	12	0.003	0.1
	Bregma-0.14	5,926	9	20	75	2	15	2	0.2	13	0.003	0.1
	Bregma-0.19	5,803	11	18	76	2	15	2	0.2	13	0.003	0.1
	Bregma-0.24	5,543	11	17	74	2	14	2	0.2	12	0.003	0.1
	Bregma-0.04+-0.09+-0.14+-0.19+-0.24	28,317	49	-	-	-	-	11	-	-	-	-
DLPFC	151507	4,226	8	18	57	2	-	2	4	9	0.002	-
	151508	4,384	8	32	58	3	-	2	4	10	0.002	-
	151509	4,789	10	35	61	3	-	2	6	11	0.002	-
	151510	4,634	10	30	56	3	-	2	5	10	0.002	-
	151669	3,661	5	27	49	1	-	2	4	8	0.002	-
	151670	3,498	8	25	47	1	-	2	4	8	0.002	-
	151671	4,110	7	30	52	2	-	2	4	9	0.002	-
	151672	4,015	10	30	51	2	-	2	4	9	0.002	-
	151673	3,639	9	30	49	1	-	2	3	9	0.002	-
	151674	3,673	6	28	49	3	-	2	5	9	0.002	-
	151675	3,592	6	26	48	1	-	2	4	8	0.002	-
	151676	3,460	6	22	27	2	-	2	3	8	0.002	-
	151507-151510	18,033	25	-	-	-	-	8	-	-	-	-
	151669-151672	15,284	20	-	-	-	-	6	-	-	-	-
	151673-151676	14,364	17	-	-	-	-	4	-	-	-	-

Note: cases not applicable are denoted by "-"

Table S1: Computing time and memory usage of all methods. Computation was carried out on a single thread of an Intel(R) Xeon(R) Gold 6138 CPU @ 2.00GHz. The computing time and memory usage were examined for all the real data applications.

Supplementary notes:

1 BASS algorithm

1.1 Model specifications

The detailed model setup is described in the Methods section. Briefly, we consider that the spatial transcriptomic study measures gene expression values for a common set of P genes from L different tissue sections, each containing $N_l, l \in \{1, \dots, L\}$, cells along with their spatial location information. We assume that the cells across all tissue sections belong to C different cell types and the tissue consists of R different spatial domains, each characterized by a distinct cell type composition. We denote $c_i^{(l)}$ as the cell type label and $z_i^{(l)}$ as the spatial domain label for the ith cell on tissue section l, with $c_i^{(l)} \in \{1, \dots, C\}$ and $z_i^{(l)} \in \{1, \dots, R\}$. To simplify the algebra, we further denote $\boldsymbol{c}^{(l)} = \left(c_1^{(l)}, \dots, c_{N_l}^{(l)}\right)^T$ and $\boldsymbol{z}^{(l)} = \left(z_1^{(l)}, \dots, z_{N_l}^{(l)}\right)^T$. We denote $\boldsymbol{\pi}_r = (\pi_{1r}, \dots, \pi_{Cr})^T$ as the C-vector of the cell type composition in the rth spatial domain, where π_{cr} represents the proportion of cell type c in the spatial domain r, with $\sum_{c=1}^{C} \pi_{cr} = 1$. For the gene expression measurements, we combine cells across all L tissue sections, conduct library size normalization followed by a log2-transformation (after adding a pseudo-count of 1), and perform dimension reduction on the normalized expression matrix to extract J low-dimensional expression features. We denote $\boldsymbol{X}^{(l)}$ as the resulting $N_l \times J$ low-dimensional expression feature matrix for section l, where $\boldsymbol{x}_i^{(l)}$ is the J-vector of expression features for the ith cell there, with $i \in \{1, \dots, N_l\}$. With the extracted low-dimensional expression features, we performed data alignment and batch effect adjustment to align expression data from different tissue sections using Harmony per recommendation of Tran et al. [68]. For the spatial location information, we construct a neighborhood graph $V^{(l)}$ among cells on each tissue section l by identifying for each cell its k nearest neighbors. With both expression and location information, we consider the following equations to model the relationship among gene expression features, cell type labels, spatial domain labels, cell type compositions, and neighborhood graphs in a hierarchical fashion:

$$\boldsymbol{x}_i^{(l)} | c_i^{(l)} = c \sim MVN(\boldsymbol{\mu}_c, \boldsymbol{\Sigma}), \quad (1)$$

$$c_i^{(l)} | z_i^{(l)} = r \sim Cat(\boldsymbol{\pi}_r), \quad (2)$$

$$\boldsymbol{z}^{(l)} \sim Potts\left(V^{(l)}, \beta\right). \quad (3)$$

Above, the first equation models the expression feature of the ith cell on section l, $\boldsymbol{x}_i^{(l)}$, as depending on its cell type label $c_i^{(l)}$. In particular, conditional on the ith cell belonging to the cell type c, $\boldsymbol{x}_i^{(l)}$ follows a multivariate normal distribution with a c-cell-type-specific mean parameter vector $\boldsymbol{\mu}_c$ and a variance-covariance matrix $\boldsymbol{\Sigma}$. The second equation models the probability of the ith cell belonging to the cell type c as depending on the underlying spatial domain. In particular, conditional on the ith cell belonging to the spatial domain r, $c_i^{(l)}$ follows a categorical distribution characterized by the r-domain-specific cell type composition vector $\boldsymbol{\pi}_r$. The third equation models the spatial domain label of the ith cell on section l, $z_i^{(l)}$, as a function of the neighborhood graph $V^{(l)}$ through a homogeneous Potts model characterized by an interaction parameter β. The probability mass function of the corresponding Potts model is defined as

$$\Pr\left(\boldsymbol{z}^{(l)} | V^{(l)}, \beta\right) = \frac{1}{C^{(l)}(\beta)} \exp\left\{\beta \sum_{i \sim i'} I\left(z_i^{(l)} = z_{i'}^{(l)}\right)\right\}, \tag{4}$$

where $i \sim i'$ denotes all neighboring pairs in the graph $V^{(l)}$; $I(z_i^{(l)} = z_{i'}^{(l)})$ is an indicator function that equals 1 if both the ith and i'th cells belong to the same spatial domain and equals 0 otherwise; β is the interaction parameter that determines the extent of spatial domain similarity among neighboring locations; and $C^{(l)}(\beta)$ is the normalizing constant, also known as the partition function, that ensures the above probability mass function to have a summation of one across all possible configurations of $\boldsymbol{z}^{(l)}$.

1.2 Prior specifications

We treat all the hyper-parameters in the above equations $(\boldsymbol{\mu}_c, \boldsymbol{\Sigma}, \boldsymbol{\pi}_r, \beta)$ as unknown and specify priors on them in order to infer them based on the data at hand. Specifically, we specify a normal-gamma prior on $\boldsymbol{\mu}_c$:

$$\mu_{jc} | \lambda_j \sim Normal\left(d_j, \lambda_j R_j^2\right), \tag{5}$$

$$d_j \propto 1 \tag{6}$$

$$\lambda_j \sim Gamma(v_1, v_2). \tag{7}$$

Above, we assume that in each feature dimension j, the mean parameter of cell type c, μ_{jc}, follows a normal distribution with a feature-specific mean parameter d_j and a variance parameter $\lambda_j R_j^2$, where λ_j is a feature-specific scaling factor following *a priori* a gamma distribution with

parameters v_1 and v_2 and R_j is the range of the jth expression feature. To simplify the algebra, we further denote $\boldsymbol{d} = (d_1, \ldots, d_J)^T$ and $\boldsymbol{\Lambda} = diag(\lambda_1 R_1^2, \ldots, \lambda_J R_J^2)$. The normal-gamma prior is applied as a shrinkage prior on the mean parameter of expression features. Intuitively, it pulls together the mean parameters of different cell types $(\mu_{j1}, \ldots, \mu_{jC})$ by shrinking λ_j to a relatively small value if the jth feature is uninformative for distinguishing different cell types, thus yielding more precise estimates of the mean parameters. Following Malsiner-Walli et al. [75], we specify hyper-parameters v_1 and v_2 to be 0.5 to allow considerable shrinkage of the prior variance of the mean parameters.

For the other parameters $(\boldsymbol{\Sigma}, \boldsymbol{\pi}_r, \beta)$, we specify the following priors:

$$\boldsymbol{\Sigma} \sim Wishirt^{-1}(n_0, \boldsymbol{\Psi}_0), \tag{8}$$

$$\boldsymbol{\pi}_r \sim Dir(\alpha_0 \mathbf{1}_C^T), \tag{9}$$

$$\beta \sim Uniform(0, \beta_{max}). \tag{10}$$

Above, we place an inverse Wishart prior on $\boldsymbol{\Sigma}$ with n_0 degrees of freedom and a symmetric positive definite scale matrix $\boldsymbol{\Psi}_0$ of size $J \times J$; a Dirichlet prior on $\boldsymbol{\pi}_r$ with concentration parameter α_0; and a uniform prior on β with a lower bound of 0 and an upper bound of β_{max}. We set n_0 to be 1 and $\boldsymbol{\Psi}_0$ to be $\mathbf{I}$ to provide a weak prior on the covariance matrix; set α_0 to be 1 to encode equal prior probabilities for all possible cell type compositions; and set β_{max} to be 4 to represent the extreme case where the spatial domain boundaries are extremely smooth.

1.3 Posterior sampling algorithm

With the above model setup and prior specifications, we develop a Gibbs sampling algorithm in combination with a Metropolis-Hastings algorithm to infer all the parameters including $c_i^{(l)}$, $z_i^{(l)}$, $\boldsymbol{\pi}_r$, β, μ_c, $\boldsymbol{\Sigma}$, d_j, and λ_j.

1.3.1 Posterior sampling of cell type labels $\boldsymbol{c}_i^{(l)}$:

The full conditional distribution of the cell type labels takes the form of a categorical distribution, where the probability of being cell type c for the ith cell on tissue section l is given by:

$$f\left(c_i^{(l)} = c \mid \cdot\right) \propto f\left(\boldsymbol{x}_i^{(l)} \mid c_i^{(l)} = c, \boldsymbol{\mu}_c, \boldsymbol{\Sigma}\right) f\left(c_i^{(l)} = c \mid z_i^{(l)} = r, \pi_{c,r}\right)$$
$$\propto \exp\left\{-\frac{1}{2}\left(\boldsymbol{x}_i^{(l)} - \boldsymbol{\mu}_c\right)^T \boldsymbol{\Sigma}^{-1}\left(\boldsymbol{x}_i^{(l)} - \boldsymbol{\mu}_c\right)\right\} \cdot \pi_{c,r}. \tag{11}$$

1.3.2 Posterior sampling of $z^{(l)}$ with the Swendsen-Wang algorithm:

We introduce auxiliary variables, $u_{i,i'}$, between every pair of cells to facilitate the posterior sampling of the spatial domain labels ($\boldsymbol{z}^{(l)}$) in each tissue section. Conditional on $\boldsymbol{z}^{(l)}$, $u_{i,i'}$ takes a uniform distribution with the following form:

$$f\left(u_{i,i'}|\boldsymbol{z}^{(l)},\beta\right) \propto e^{-\beta I\left(z_i^{(l)}=z_{i'}^{(l)}\right)} I\left(0 \leq u_{i,i'} \leq e^{\beta I\left(z_i^{(l)}=z_{i'}^{(l)}\right)}\right). \quad (12)$$

Then, the full conditional distribution of $\boldsymbol{z}^{(l)}$ given the auxiliary variables and all the other parameters ($\boldsymbol{c}^{(l)}, \boldsymbol{\pi}, \beta$) takes the following form:

$$f\left(\boldsymbol{z}^{(l)}|\boldsymbol{u},\beta,\boldsymbol{c}^{(l)},\boldsymbol{\pi};V^{(l)}\right)$$

$$\propto f\left(\boldsymbol{z}^{(l)}|\beta;V^{(l)}\right) f\left(\boldsymbol{u}|\boldsymbol{z}^{(l)},\beta\right) f\left(\boldsymbol{c}^{(l)}|\boldsymbol{z}^{(l)},\boldsymbol{\pi}\right)$$

$$\propto \prod_{i\sim i'} e^{\beta I(z_i^{(l)}=z_{i'}^{(l)})} \prod_{i\sim i'} e^{-\beta I\left(z_i^{(l)}=z_{i'}^{(l)}\right)} I\left(0 \leq u_{i,i'} \leq e^{\beta I\left(z_i^{(l)}=z_{i'}^{(l)}\right)}\right) \prod_{i=1}^{N_l} \pi_{c_i^{(l)},z_i^{(l)}}$$

$$\propto \prod_{i=1}^{N_l} \pi_{c_i^{(l)},z_i^{(l)}} \prod_{i\sim i'} I\left(0 \leq u_{i,i'} \leq e^{\beta I\left(z_i^{(l)}=z_{i'}^{(l)}\right)}\right). \quad (13)$$

The Swendsen-Wang algorithm proceeds by first sampling the auxiliary variables $\boldsymbol{u}$ given the spatial domain labels $\boldsymbol{z}^{(l)}$ based on Eq. (12). As will be indicated below, it only matters whether $u_{i,i'}$ is greater than 1 or not. Therefore, it suffices to sample $I(u_{i,i'} > 1)$ and we claim two cells are bonded if $I\left(u_{i,i'} > 1\right) = 1$. The auxiliary variable $u_{i,i'}$ is thus often referred to as a bond variable. Consequently, we can sample $I(u_{i,i'} > 1)$ with the following steps: if $z_i^{(l)} \neq z_{i'}^{(l)}$, then $I\left(u_{i,i'} > 1\right) = 0$; and if $z_i^{(l)} = z_{i'}^{(l)}$, then we sample $I\left(u_{i,i'} > 1\right)$ from a Bernoulli distribution with probability $1 - e^{-\beta}$. Next, we sample the spatial domain labels $\boldsymbol{z}^{(l)}$ given the auxiliary variables based on Eq. (13). Specifically, if two cells are bonded (i.e., $I\left(u_{i,i'} > 1\right) = 1$), then the spatial domain labels of the two cells must be the same (i.e., $z_i^{(l)} = z_{i'}^{(l)}$). On the other hand, if two cells are not bonded, then there is no constraint on the spatial domain labels of the two cells. Consequently, the bond variable partitions all cells into multiple clusters of cells that belong to the same spatial domain. For cell cluster K, the probability of belonging to spatial domain r is:

$$\Pr\left(\boldsymbol{z}_K^{(l)} = \boldsymbol{r}|\cdot\right) \propto \prod_{i\in K} \pi_{c_i^{(l)},z_i^{(l)}}, \quad (14)$$

where $\boldsymbol{z}_K^{(l)} = \boldsymbol{r}$ is a shorthand notation for $z_i^{(l)} = r$ for all $i \in K$.

1.3.3 Posterior sampling of cell type compositions $\boldsymbol{\pi}_r$:

The full conditional distribution of the cell type composition vector, $\boldsymbol{\pi}_r$, takes the following form:

$$f(\boldsymbol{\pi}_r|\boldsymbol{c},\boldsymbol{z}) \propto f(\boldsymbol{c}|\boldsymbol{z},\boldsymbol{\pi}_r)f(\boldsymbol{\pi}_r) \propto \prod_c \pi_{c,r}^{\sum_l |U^{(l)}(c,r)|+\alpha_0-1}, \tag{15}$$

where $U^{(l)}(c,r)$ is the index set of cells with cell type label c and spatial domain label r in the lth tissue section and $|\cdot|$ is the cardinality of the corresponding index set. Therefore, the full conditional distribution of $\boldsymbol{\pi}_r$ takes the form of a Dirichlet distribution, that is

$$\boldsymbol{\pi}_r|\boldsymbol{c},\boldsymbol{z} \sim Dir\left(\sum_l \left|U^{(l)}(1,r)\right| + \alpha_0, \dots, \sum_l \left|U^{(l)}(C,r)\right| + \alpha_0\right). \tag{16}$$

1.3.4 Posterior sampling of $\boldsymbol{\beta}$:

The full conditional distribution of β takes the following form:

$$\begin{aligned} &f\left(\beta\middle|\boldsymbol{z}^{(1)}, \dots \boldsymbol{z}^{(L)}; V^{(1)}, \dots V^{(L)}\right) \\ &= \prod_l f\left(\boldsymbol{z}^{(l)}\middle|V^{(l)}\right) f(\beta) \\ &= \prod_l \frac{1}{C^{(l)}(\beta)} e^{\beta \sum_{i \sim i'} I\left(z_i^{(l)} = z_{i'}^{(l)}\right)} I(0 \le \beta \le \beta_{max}). \end{aligned} \tag{17}$$

The hyper-parameter β in the Potts model is difficult to infer algorithmically because of the normalization constant $C^{(l)}(\beta)$. In particular, the computation of $C^{(l)}(\beta)$ requires evaluating the probability mass function of the Potts model over all possible configurations of $\boldsymbol{z}^{(l)}$ and is thus known to be NP hard. Instead of computing the normalizing constant directly, we estimate the ratio of two normalizing constants by adapting the Swendsen-Wang algorithm, which allows us to sample β from its conditional distribution through a Metropolis-Hastings algorithm. Specifically, we use a uniform distribution centered at the current value of β as our proposal distribution, where the step size ϵ is set to be 0.1 by default. Then, the acceptance probability for the proposed value β' is given by:

$$A = \min\left\{1, \frac{f\left(\beta'|\boldsymbol{z}^{(1)}, \dots \boldsymbol{z}^{(L)}; V^{(1)}, \dots V^{(L)}\right)}{f\left(\beta|\boldsymbol{z}^{(1)}, \dots \boldsymbol{z}^{(L)}; V^{(1)}, \dots V^{(L)}\right)}\right\} \tag{18}$$

$$\Rightarrow A = \min\left\{1, \frac{\prod_l C^{(l)}(\beta) e^{\beta' \sum_{i\sim i'} I\left(z_i^{(l)} = z_{i'}^{(l)}\right)}}{\prod_l C^{(l)}(\beta') e^{\beta \sum_{i\sim i'} I\left(z_i^{(l)} = z_{i'}^{(l)}\right)}} I(0 \le \beta' \le \beta_{max})\right\}. \quad (19)$$

There, the ratio of the normalizing constant, $\frac{C^{(l)}(\beta)}{C^{(l)}(\beta')}$, can be represented with the following derivations:

$$\sum_{\{\mathbf{z}^{(l)}\}} f\left(\mathbf{z}^{(l)} | V^{(l)}, \beta\right) = 1$$

$$\Rightarrow \sum_{\{\mathbf{z}^{(l)}\}} \frac{1}{C^{(l)}(\beta)} e^{\beta \sum_{i\sim i'} I\left(z_i^{(l)} = z_{i'}^{(l)}\right)} = 1$$

$$\Rightarrow C^{(l)}(\beta) = \sum_{\{\mathbf{z}^{(l)}\}} e^{\beta \sum_{i\sim i'} I\left(z_i^{(l)} = z_{i'}^{(l)}\right)}$$

$$\Rightarrow C^{(l)}(\beta) = \sum_{\{\mathbf{z}^{(l)}\}} e^{(\beta-\beta') \sum_{i\sim i'} I\left(z_i^{(l)} = z_{i'}^{(l)}\right)} C^{(l)}(\beta') f\left(\mathbf{z}^{(l)} | V^{(l)}, \beta'\right)$$

$$\Rightarrow \frac{C^{(l)}(\beta)}{C^{(l)}(\beta')} = \sum_{\{\mathbf{z}^{(l)}\}} e^{(\beta-\beta') \sum_{i\sim i'} I\left(z_i^{(l)} = z_{i'}^{(l)}\right)} f\left(\mathbf{z}^{(l)} | V^{(l)}, \beta'\right)$$

$$\Rightarrow \frac{C^{(l)}(\beta)}{C^{(l)}(\beta')} = E_{\mathbf{z}^{(l)}|\beta'}\left(e^{(\beta-\beta') \sum_{i\sim i'} I\left(z_i^{(l)} = z_{i'}^{(l)}\right)}\right). \quad (20)$$

We approximate the ratio of the two normalizing constants by sampling from the Potts model $f\ (\mathbf{z}^{(l)}|\beta')$ and estimating the expectation with the sample mean. We apply the Swendsen-Wang algorithm by following similar procedures as described in section 1.3.2 to draw samples from the Potts model. By default, we collect 10 Potts samples after 10 burn-in steps for the approximation.

The approximation of the normalizing constants creates a heavy computational burden on the algorithm. Therefore, when analyzing multiple tissue sections, we infer β based on the first tissue section to reduce the computational burden. Then, the acceptance probability in Eq.(19) reduces to

$$A = \min\left\{1, \frac{C^{(1)}(\beta) e^{\beta' \sum_{i\sim i'} I\left(z_i^{(1)} = z_{i'}^{(1)}\right)}}{C^{(1)}(\beta') e^{\beta \sum_{i\sim i'} I\left(z_i^{(1)} = z_{i'}^{(1)}\right)}} I(0 \le \beta' \le \beta_{max})\right\}, \quad (21)$$

where the ratio of the normalizing constant, $\frac{C^{(1)}(\beta)}{C^{(1)}(\beta')}$, can be approximated based on

$$\frac{C^{(1)}(\beta)}{C^{(1)}(\beta')} = E_{\boldsymbol{z}^{(1)}|\beta'}\left(e^{(\beta-\beta')\sum_{i\sim i'} I\left(z_i^{(1)}=z_{i'}^{(1)}\right)}\right). \tag{22}$$

In addition, the BASS software implements a stopping rule to automatically determine the number of MCMC samples required for inferring β. Specifically, we calculate the mean of the sampled β in every 100 iterations and stop the sampling if the difference in the consecutive two means is below a certain threshold (0.001 by default). Such stopping rule can help improve computation efficiency and works well in all our real data applications (Fig. S22).

1.3.5 Posterior sampling of $\boldsymbol{\mu}_c$:

The full conditional distribution of the mean parameter of gene expression features takes the following parametric form:

$$f\left(\boldsymbol{\mu}_c|\boldsymbol{X}^{(1)},\dots,\boldsymbol{X}^{(L)},\boldsymbol{\Sigma},\boldsymbol{d},\boldsymbol{\Lambda}\right)$$

$$\propto \prod_l \prod_{i\in T^{(l)}(c)} f\left(\boldsymbol{x}_i^{(l)}|\boldsymbol{\mu}_c,\boldsymbol{\Sigma}\right) f(\boldsymbol{\mu}_c|\boldsymbol{d},\boldsymbol{\Lambda})$$

$$\propto \exp\left\{-\frac{1}{2}\sum_l \sum_{i\in T^{(l)}(c)} \left(\boldsymbol{x}_i^{(l)}-\boldsymbol{\mu}_c\right)^T \boldsymbol{\Sigma}^{-1}\left(\boldsymbol{x}_i^{(l)}-\boldsymbol{\mu}_c\right)\right\} \exp\left\{-\frac{1}{2}(\boldsymbol{\mu}_c-\boldsymbol{d})^T\boldsymbol{\Lambda}^{-1}(\boldsymbol{\mu}_c-\boldsymbol{d})\right\}$$

$$\propto \exp\left\{-\frac{1}{2}\boldsymbol{\mu}_c^T\left(\sum_l \left|T^{(l)}(c)\right|\boldsymbol{\Sigma}^{-1}+\boldsymbol{\Lambda}^{-1}\right)\boldsymbol{\mu}_c + \boldsymbol{\mu}_c^T\left(\sum_l \sum_{i\in T^{(l)}(c)} \boldsymbol{\Sigma}^{-1}\boldsymbol{x}_i^{(l)}+\boldsymbol{\Lambda}^{-1}\boldsymbol{d}\right)\right\}, \tag{23}$$

where $T^{(l)}(c)$ is the index set of cells with cell type label c on tissue section l and $|\cdot|$ is the cardinality of the corresponding index set. Therefore, the full conditional distribution of $\boldsymbol{\mu}_c$ takes the form of a normal distribution, that is

$$\boldsymbol{\mu}_c|\cdot \sim Normal(\boldsymbol{\mu}_c^*,\boldsymbol{\Sigma}_c^*), \qquad c=1,\dots,C, \tag{24}$$

where

$$\boldsymbol{\mu}_c^* = \boldsymbol{\Sigma}_c^*\left(\sum_l \sum_{i\in T^{(l)}(c)} \boldsymbol{\Sigma}^{-1}\boldsymbol{x}_i^{(l)}+\boldsymbol{\Lambda}^{-1}\boldsymbol{d}\right) \tag{25}$$

$$\boldsymbol{\Sigma}_c^{*-1} = \sum_l \left|T^{(l)}(c)\right|\boldsymbol{\Sigma}^{-1}+\boldsymbol{\Lambda}^{-1} \tag{26}$$

1.3.6 Posterior sampling of $\boldsymbol{\Sigma}$:

The full conditional distribution of the variance-covariance parameter of gene expression features takes the following parametric form:

$$f\left(\boldsymbol{\Sigma}|\boldsymbol{X}^{(1)},\dots,\boldsymbol{X}^{(L)},\boldsymbol{\mu}_1,\dots,\boldsymbol{\mu}_C\right)$$
$$\propto \prod_l \prod_i f\left(\boldsymbol{x}_i^{(l)}|\boldsymbol{\mu}_{c_i^{(l)}},\boldsymbol{\Sigma}\right) f(\boldsymbol{\Sigma})$$
$$\propto |\boldsymbol{\Sigma}|^{-\frac{\sum_l N_l}{2}} \exp\left\{-\frac{1}{2}\sum_l \sum_i \left(\boldsymbol{x}_i^{(l)} - \boldsymbol{\mu}_{c_i^{(l)}}\right)^T \boldsymbol{\Sigma}^{-1}\left(\boldsymbol{x}_i^{(l)} - \boldsymbol{\mu}_{c_i^{(l)}}\right)\right\} |\boldsymbol{\Sigma}|^{-\frac{n_0+J+1}{2}} \exp\left\{-\frac{1}{2} tr(\boldsymbol{\Psi}_0 \boldsymbol{\Sigma}^{-1})\right\}$$
$$\propto |\boldsymbol{\Sigma}|^{-\frac{\sum_l N_l + n_0 + J + 1}{2}} \exp\left\{-\frac{1}{2} tr\left[\left(\sum_l \sum_i \left(\boldsymbol{x}_i^{(l)} - \boldsymbol{\mu}_{c_i^{(l)}}\right)\left(\boldsymbol{x}_i^{(l)} - \boldsymbol{\mu}_{c_i^{(l)}}\right)^T + \boldsymbol{\Psi}_0\right)\boldsymbol{\Sigma}^{-1}\right]\right\}, \quad (27)$$

which is the kernel of an inverse Wishart distribution. Therefore, we have

$$\boldsymbol{\Sigma}| \cdot \sim Wishart^{-1}(n_0^*, \boldsymbol{\Psi}_0^*), \quad (28)$$

where

$$\boldsymbol{\Psi}_0^* = \sum_l \sum_i \left(\boldsymbol{x}_i^{(l)} - \boldsymbol{\mu}_{c_i^{(l)}}\right)\left(\boldsymbol{x}_i^{(l)} - \boldsymbol{\mu}_{c_i^{(l)}}\right)^T + \boldsymbol{\Psi}_0 \quad (29)$$

$$n_0^* = \sum_l N_l + n_0 \quad (30)$$

1.3.7 Posterior sampling of d_j:

The full conditional distribution of $\boldsymbol{d}$ takes the following parametric form:

$$f(\boldsymbol{d}|\boldsymbol{\mu}_1,\dots,\boldsymbol{\mu}_C,\boldsymbol{\Lambda})$$
$$\propto \prod_c f(\boldsymbol{\mu}_c|\boldsymbol{d},\boldsymbol{\Lambda}) f(\boldsymbol{d})$$
$$\propto \prod_c \exp\left\{-\frac{1}{2}(\boldsymbol{\mu}_c - \boldsymbol{d})^T \boldsymbol{\Lambda}^{-1}(\boldsymbol{\mu}_c - \boldsymbol{d})\right\}$$
$$\propto \prod_j \exp\left\{-\frac{\sum_c \left(\mu_{jc} - d_j\right)^2}{2\lambda_j R_j^2}\right\}$$
$$\propto \prod_j \exp\left\{-\frac{\left(d_j - \frac{1}{C}\sum_c \mu_{jc}\right)^2}{2\lambda_j R_j^2 / C}\right\}. \quad (31)$$

which is the kernel of a normal distribution. Therefore, we have

$$d_j| \cdot \sim N\left(\frac{1}{C}\sum_c \mu_{jc}, \frac{1}{C}\lambda_j R_j^2\right). \quad (32)$$

1.3.8 Posterior sampling of λ_j:

The full conditional distribution of λ_j takes the following parametric form:

$$
\begin{aligned}
& f(\lambda_j | \mu_{j1}, \dots, \mu_{jC}, d_j) \\
& \propto \prod_c f(\mu_{jc} | d_j, \lambda_j) f(\lambda_j) \\
& \propto \lambda_j^{-\frac{C}{2}} \exp\left\{ \frac{\sum_c (\mu_{jc} - d_j)^2}{2\lambda_j R_j^2} \right\} \lambda_j^{v_1 - 1} \exp\{-v_2 \lambda_j\} \\
& \propto \lambda_j^{v_1 - \frac{C}{2} - 1} \exp\left\{ -\frac{1}{2} \left[\frac{\sum_c (\mu_{jc} - d_j)^2}{R_j^2} \cdot \frac{1}{\lambda_j} + 2 v_2 \lambda_j \right] \right\},
\end{aligned} \tag{33}
$$

which is the kernel of a generalized inverse Gaussian distribution (GIG). Therefore, we have

$$
\lambda_j | \cdot \sim GIG(a^*, b^*, p^*), \tag{34}
$$

where

$$
a^* = 2v_2
$$

$$
b^* = \frac{\sum_c (\mu_{jc} - d_j)^2}{R_j^2}
$$

$$
p^* = v_1 - \frac{C}{2}
$$

1.4 Parameter estimation

As has been discussed in the Methods section, we post-process the sampling results to mitigate the label switching issue associated with the sampling of $\boldsymbol{c}$ and $\boldsymbol{z}$ in the mixture model. In particular, we deal with the label switching problem by post-processing the posterior samples based on the iterative version 1 of the equivalence class representation (ECR) algorithm implemented in the label.switching package (version 1.8). Finally, we estimate $\boldsymbol{c}$ and $\boldsymbol{z}$ as the mode of all their posterior samples and estimate $\boldsymbol{\pi}_r$ using the final estimates of $\boldsymbol{c}$ and $\boldsymbol{z}$. In particular, we have $\pi_{cr} = \frac{\sum_l \sum_i I(c_i^{(l)}=c, z_i^{(l)}=r)}{\sum_l \sum_i I(z_i^{(l)}=r)}$ and if $\sum_l \sum_i I(z_i^{(l)} = r) = 0$, we set $\boldsymbol{\pi}_r = \boldsymbol{0}$. We did not estimate $\boldsymbol{\pi}_r$ by summarizing its posterior samples because the label switching issue could not be fully resolved by the ECR-1 algorithm and the posterior mean or posterior median estimates of $\boldsymbol{\pi}_r$ could be sensitive to the label switching issue. On the other hand, the posterior estimates of $\boldsymbol{c}$ and $\boldsymbol{z}$ were less sensitive to the label switching issue, allowing us to estimate $\boldsymbol{\pi}_r$ more accurately.
