# Supplementary material for: BASS: multi-scale and multi-sample analysis enables accurate cell type clustering and spatial domain detection in spatial transcriptomic studies
Source: Genome Biol. 2022 Aug 4;23:168. doi: 10.1186/s13059-022-02734-7 (PMC9351148; doi:10.1186/s13059-022-02734-7)
Supplement: Supplementary file 2 — Additional file 2. Review History. [file 13059_2022_2734_MOESM2_ESM.docx]

Review History

**First round of review**

**Reviewer 1**

**Were you able to assess all statistics in the manuscript, including the appropriateness of statistical tests used?**

No

**Comments to author:**

This paper presents a method BASS for simultaneous clustering of cell types and spatial domains in spatial transcriptomics data. The method is based a Bayesian hierarchical modeling framework, and enables multi-scale and multi-sample analysis. The authors used several simulation experiments and three real spatial transcriptomic datasets to illustrate the performance of the proposed method, comparing to several existing methods for similar tasks. This is a promising work, but there are several aspects that may need improvement.

1. The biological significance of the work was not well presented. The authors showed improvements of some major criteria in comparing with peer methods, but didn't put effort to explain or discuss the biological significance of the degree of improvement to the biology behind the data. In fact, the paper didn't try to explain the backgrounds of the data they used for experiments. Readers will have a better understanding on the advantage of proposed method if we can read about the biological significance of the results.

2. The presented experiments are based on known settings of the number of clusters and number of spatial domains. What if we don't know them in advance or what we assumed were wrong? What is the impact of the number of cell clusters on the spatial domain segmentation, and what is impact of the number of spatial domains on the clustering results?

3. The multi-section analysis is a unique feature of the proposed method. What is the major application scenario of such analysis? Is there any requirement on the data for using this type of analysis?

4. In section 4.1, the author reported the value changes of interaction parameter beta of the HMRF method. So what are the values inferred by BASS? Do they also differ under different simulation settings?

5. Some typos exist in the manuscript.

**Reviewer 2**

**Were you able to assess all statistics in the manuscript, including the appropriateness of statistical tests used?**

Ya

**Comments to author:**

Here, Li and Zhou present BASS, a computational tool for spatial domain detection and unsupervised clustering of spatial transcriptomics studies. In recent years, technologies for high-throughput, high-resolution profiling of the transcriptome have become more popular. The advantage over traditional scRNA-seq is obvious as spatial information is provided in addition to transcriptome profile. However, to take full advantage of these datasets, novel computational methods are required. BASS addresses one of the central challenges in scRNA-seq - unsupervised clustering - as well as the closely related problem of identifying domains in spatial data. As there are currently several complementary technologies available, there are additional challenges for developing computational methods as one would ideally like each method to be applicable across multiple technologies. The authors demonstrate that BASS outperforms existing methods across several platforms which is crucial for this type of method.

I would like for the authors to address the following major issues:

1 Also take run time and memory usage into consideration when reporting on the performance of the different methods. In particular, users will be interested in learning how well methods scale with the size of the data.

2 Although the ARI is a useful metric for evaluating clustering performance, it is not without flaws. One particular issue is the fact that it can give a relatively small penalty if small cluster of cells is miscategorized. However, from a biological point of view this can be undesirable as there are many applications where one is interested in rare cell populations. Thus, I would like for the authors to comment on how BASS (and other methods) perform with regards to small clusters. It would be ideal if this aspect was investigated as part of the simulations. I think the setup of the simulations is nice, but the main shortcoming is that they only contain four cell types which is not realistic for most real use cases.

3 Similarly, sometimes the ARI can be overly pessimistic. For example if a large cluster is split into two this can result in a poor score when in reality it may not be such a bad decision. I would like for the authors to check if this is the case for any of the methods and datasets to make sure that erroneous conclusions are avoided.

4 Different technologies will typically differ in terms of the number of transcripts that they are able to profile, ranging from ~100 to the full transcriptome. It would be interesting to understand how the performance of BASS (and other methods) depends on the number of genes used. Could the authors carry out simulations where genes (rows) are randomly excluded from the count matrix to understand how many genes are required for good performance? Related to this point, is there some way that the method can inform the user which genes are most informative in terms of the clustering? This information could be very helpful to users interested in developing novel gene panels for future experiments.

5 In fig 8 the ARI results are shown as a jitter plot, including the results from multiple sections. It is not clear to me why the results in fig 4 & 6 were not displayed in a similar manner as there are clearly several sections available.

6 I find it interesting that BASS shows drastically better performance compared to HMRF and BayesSpace, even though all three methods are based on the Potts model. I would like the authors to comment on this in the discussion and provide their interpretation of what aspect of their model is responsible for the improvements.

7 What happened for some of the points in 3d for 5 and 10 slices? These are clearly outliers, so I wonder if the authors could analyze why their algorithm has failed here? Except for those examples, the method performs extremely well for all four scenarios and this worries me a bit. In particular since the performance is never this good for any of the real datasets, I am concerned that the simulated data is a bit too easy here. I would like for the authors to alter some of the parameters to ensure that the simulations become more challenging and hence more representative of real data.

8 I was able to successfully download, install and run BASS using a Nanostring dataset without any problems. As I do not have a ground truth here, it is hard to judge the quality of the results but overall the output seemed very reasonable, so the fact that the method worked on a platform that had not been tested in the manuscript is a very good thing.

Minor issues:

I find it a little strange that sections 3.5 and 3.6 are named by the method, section 3.7 is named by the brain region rather than the method used. Could the authors explain why this is the case?

**Authors Response**

**Point-by-point responses to the reviewers’ comments:**

Reviewer #1: This paper presents a method BASS for simultaneous clustering of cell types and spatial domains in spatial transcriptomics data. The method is based on a Bayesian hierarchical modeling framework, and enables multi-scale and multi-sample analysis. The authors used several simulation experiments and three real spatial transcriptomic datasets to illustrate the performance of the proposed method, comparing to several existing methods for similar tasks. This is a promising work, but there are several aspects that may need improvement.

*Responses: Thank you for your positive and constructive comments. Our detailed responses to your specific comments are listed below.*

1. The biological significance of the work was not well presented. The authors showed improvements of some major criteria in comparing with peer methods but didn't put effort to explain or discuss the biological significance of the degree of improvement to the biology behind the data. In fact, the paper didn't try to explain the backgrounds of the data they used for experiments. Readers will have a better understanding on the advantage of proposed method if we can read about the biological significance of the results.

*Response: Thank you for the comment. We apologize for not making the biological significance of the degree of improvement clear in the previous version of the manuscript. Following your comments, we have included additional details to explain the background of the data and highlight the biological significance of the results. Specifically, we applied BASS to analyze three spatial transcriptomic datasets. In all three applications, BASS accurately identified cell types and spatial domains on the tissue that were missed by the other methods, thus providing important biological insights into the transcriptomic and cellular landscape in the cortex and hypothalamus. For example, the MERFISH data contains spatial transcriptomic measurements on the hypothalamus, which is an important region in the center of the brain that comprises multiple nuclei and controls many social behaviors such as reproduction and circadian rhythms as well as homeostatic functions such as neuroendocrine and cardiovascular regulation [1]. The hypothalamus consists of a diverse array of neuronal and glial cell types, many of which are spatially segregated in distinct anatomical structures, interconnecting the other parts of the central nervous system such as brainstem and areas of the autonomous nervous system [2, 3]. In this data, only BASS accurately revealed eight brain structures (Figure 6) and delineated the excitatory neuronal subtypes (e.g., E1, E2, and E3) and inhibitory neuronal subtypes (e.g., I1 and I2; Figure 7B), allowing us to fully characterize the spatial distribution of cell types across distinct functional nuclei/structures in the hypothalamus (Figures 7G and S30). In contrast, the other methods all failed to identify certain brain structures (Figure 6) or neuronal subtypes (Figure 7C-E). For example, none of the other domain detection methods could identify and segregate the periventricular hypothalamic nucleus (PV) region and the medial preoptic nucleus (MPN) region (Figure 6D). In addition, none of the other cell type clustering methods could identify and segregate the inhibitory neuronal subtype I2 located in the PV region (Figures 7C-E and S30B). PV and MPN regions possess important structural and functional roles in the nervous system. Specifically, PV consists of a thin sheet of small neurons located in the wall of the third ventricle and these neurons regulate the release of gonadotropin-releasing hormone (GnRH) and growth hormone (GH) [4, 5]. MPN occupies a more lateral location than PV and plays an important role in reproductive and parental behavior [6]. The capability of BASS to identify these two regions using spatial transcriptomics and characterize their compositions of cell types provided important insights into the transcriptomic basis underlying the function of these two regions.*

*The accurate detection of tissue domains by BASS also allows us to perform additional downstream analysis to further characterize the transcriptomic architecture underlying complex tissues. Here, following your comments, we have included an additional differential expression (DE) analysis to identify both cell type marker genes and domain marker genes based on the cell type or spatial domain estimates from BASS. In the analysis, we found that each cell type/spatial domain exhibit a unique expression signature, allowing us to identify important gene markers for each cell type and each spatial domain (Supplementary Figures S32-S33). In particular, in the MERFISH dataset, we identified top marker genes Slc18a2 and Scg2 for the PV domain and Calcr and Nts for the MPN domain, which are important gene candidates for future studies. Specifically, Slc18a2 (Solute Carrier Family 18 Member A2) gene encodes a transmembrane protein that facilitates the uptake of monoamine neurotransmitters into synaptic vesicles and plays an essential role in dopamine regulation [7], which inhibits gonadotrophin secretion [8-11]. Indeed, genetic variants in Slc18a2 in human have been identified to be associated with the polycystic ovary syndrome (PCOS), a hormone disorder related to ovaries [12]. The important role of Slc18a2 in gonadotrophin secretion may underlie the well-known function of PV domain on regulating the release of gonadotropin-releasing hormone [4]. As a second example, Calcr (Calcitonin Receptor) encodes a high affinity receptor for the peptide hormone calcitonin. In mice, the amylin-calcitonin receptor signaling has been reported to mediate affiliative social contacts [13]. Therefore, the identification of Calcr by BASS may provide the transcriptomic mechanism underlying the regulatory function of the MPN region in reproductive and parental behaviors [6].*

*Finally, the accurate cell types and spatial domains detected by BASS can also be paired with many other analytic tools to further improve various downstream applications to reveal additional biological insights. For example, SpatialCorr [14] is a recent method that aims to identify sets of genes with spatially varying correlation structures within or between pre-determined spatial domains. The spatial domains detected by BASS can be paired with SpatialCorr to detect genes with coordinated regulation within or between tissue regions. Knowing such coordinated regulation can help identify novel cell subpopulations and provide important insights into the gene regulatory network within the subpopulation [14]. As another example, trajectory analysis can be carried out on the cell types or the spatial domains inferred by BASS to facilitate the investigation into cancer progression, cell type differentiation, as well as structural feature development [15, 16]. In addition, because spatial transcriptomic studies often collect data from multiple adjacent sections, tools such as PASTE [17] and GPSA [18] for aligning adjacent tissue sections can be paired with BASS for 3D tissue construction [17] or 2D construction of a larger tissue area [19]. There, the accurately detected cell types and spatial domains from BASS allow us to explore the spatial domain structures and spatial distribution patterns of cell types in 3D or a larger 2D tissue area.*

*We have included the above details and discussions into the Results section at lines 696-703, 785-788, 805-811, 876-896 on pages 24, 27, 29, and 30 and the Discussion section at lines 1030-1045 on pages 34-35.*

2. The presented experiments are based on known settings of the number of clusters and number of spatial domains. What if we don't know them in advance or what we assumed were wrong? What is the impact of the number of cell clusters on the spatial domain segmentation, and what is impact of the number of spatial domains on the clustering results?

*Response: Thank you. Following your comment, we have performed additional simulations to evaluate the influence of the specified number of cell types and the number of spatial domains on method performance. The simulation details are provided in the updated Methods and material section (lines 316-325 on page 12). Specifically, we focused on the baseline simulation setting of scenario III (i.e., nGenes=200,de.prob=0.2, and de.facloc=1.1) and specified either the number of cell types or the number of spatial domains to be 2, 4, 6, 8, or 10 while fixing the other to be the truth (4). We then evaluated the impact of the two parameters on both cell type clustering and spatial domain detection for BASS and the corresponding task for the other methods based on three criteria: the overall agreement between the estimated labels and true labels measured by the adjusted random index (ARI); the number of estimated cell types and/or spatial domains; and the proportion of cells in each cell type cluster and/or spatial domain. The new results are displayed in the Supplementary Figures S16-S17 and described in the Results section (lines 600-641 on pages 21-22).*

*First, we evaluated the influence of the specified number of cell types on cell type clustering. As expected, when the number of cell types is under-specified, all methods aggregated cells of multiple types into the same clusters and produced lower ARIs than before (median ARIs = 0.36, 0.41, 0.31, and 0.37 for BASS, SC3, Seurat, and FICT; Figure S16A-C). On the other hand, when the number of cell types was over-specified, we found that BASS could still accurately assign cells of the same type into the same cluster while keeping the redundant clusters to be of minimal sizes, leading to only a small ARI loss. For example, when the number of cell types was specified to be 10 (truth = 4), the median ARI of cell type clustering from BASS was 0.90, which represented only a small reduction compared to the median ARI of 0.95 when the number of cell types was specified to be the truth (4) (Figure S16A). In this case, the top 4 clusters with the largest number of cells (Cluster 1-4; Figure S16C) corresponded to the 4 cell types, while the remaining 6 clusters were small and consisted of only an average of 6% of the total cell population (Figure S16C). In contrast, SC3, Seurat, and FICT segregated cells of the same type into multiple clusters and produced much poorer ARIs than before. Specifically, in the same example above, the median ARIs from SC3, Seurat, and FICT are 0.53, 0.53, and 0.45, respectively, which were much worse compared to the median ARIs of 0.91, 0.87, and 0.61 when the number of cell types was specified to be the truth (4). For these three methods, the 6 clusters that did not clearly correspond to the true cell types were large and consisted of an average of 30%, 41%, and 54% of cells, respectively (Figure S16C).*

*Next, we evaluated the influence of the specified number of spatial domains on spatial domain detection (Figure S17). As expected, when the number of spatial domains was under-specified, all methods tended to merge different spatial domains into large ones and produced poorer ARIs than before (median ARIs = 0.52, 0.11, 0.03, and 0.14 for BASS, HMRF, BayesSpace, and SpaGCN; Figure S17A). On the other hand, when the number of spatial domains was over-specified, similarly to what we have observed in the cell type clustering, BASS could still accurately detect the 4 spatial domains while keeping the redundant domains to be small and contained only a small proportion of cells, resulting in only a small reduction in ARI (Figure S17A-C). HMRF was also relatively robust to the over-specification of the number of spatial domains, presumably because we explored a list of spatial parameters β in HMRF and chose the oracle version of HMRF that likely over-fitted the data. In contrast, BayesSpace and SpaGCN separated one tissue domain into multiple domains, leading to substantial loss in ARI (Figure S17C).*

*Finally, we evaluated the influence of the specified number of cell types on spatial domain detection in BASS (Figure S16D-F) and the influence of the specified number of spatial domains on cell type clustering in BASS (Figure S17D-F). We found that a mis-specified number of spatial domains, regardless of whether it was under-specified or over-specified, did not have much influence on the performance of cell type clustering. An over-specified number of cell type clusters did not influence the performance of spatial domains either. Only an under-specified number of cell type clusters reduced the performance of spatial domain detection, which is presumably due to the relatively poor cell type clustering performance there.*

*Given the above results, we have added a practical guideline into the Discussion section (lines 971-987 on page 33) for selecting the optimal number of cell types and spatial domains for BASS when the underlying truths are unknown. Our recommendation is to start with a relatively large number of cell types and a relatively large number of spatial domains. We recommend to first select the optimal number of spatial domains, given that spatial domains can be robustly detected by BASS with mis-specified cell type number. To do so, we calculate the number of cells in each spatial domain and set the number of spatial domains to be the largest one that still leads to a relatively large number of cells in each domain determined by the user (e.g. with > 3% of cells per domain). After selecting the optimal number of spatial domains, we proceed to select the optimal number of cell types. Here, care needs to be taken when selecting the optimal number of cell types because the small cell type clusters with a relatively small number of cells can either be redundant clusters or represent rare cell types. Therefore, we recommend examining each small cell type cluster carefully, by, for example, performing a differential expression analysis to examine cell type cluster marker genes. Careful examination will allow us to determine whether the small cell types are rare cell types or redundant clusters, thus informing the choice of the optimal number of cell types.*

3. The multi-section analysis is a unique feature of the proposed method. What is the major application scenario of such analysis? Is there any requirement on the data for using this type of analysis?

*Response: Thank you for the comment. For your first question, many, if not most, spatial transcriptomic studies collect multiple adjacent sections from the same tissue or collect tissue samples from multiple individuals. Different sections from the same or similar tissues often contain a similar set of spatial domains and a similar composition of cell types. Consequently, modeling multiple tissue sections/samples together can borrow information across samples to potentially enhance the performance of both cell type clustering and spatial domain detection for single-cell spatial transcriptomics. Therefore, enable powerful cell type clustering and spatial domain detection for these spatial transcriptomics studies is the major application scenario of the proposed multi-section analysis. For your second question, the multi-section analysis of BASS only requires the spatial transcriptomic data to contain multiple tissue sections. For spatial transcriptomic data that contain only one tissue section, one can directly apply the single-section analysis of BASS. We have modified the Introduction section to make these two important points clear (lines 86-93 and 102-105 on pages 4-5).*

4. In section 4.1, the author reported the value changes of interaction parameter beta of the HMRF method. So what are the values inferred by BASS? Do they also differ under different simulation settings?

*Reponse: Thank you for the comment. Following your suggestion, we have included a new figure (Figure S14) to report the estimates of the parameter β across all simulation settings for the four scenarios (scenario I, II, III and IV). The new results are described in the Results section (line 535-543 on page 19). Briefly, the β estimates varied both within and across different simulation settings, highlighting the importance of estimating β in each dataset (Figure S14). In addition, the β estimates in scenario I were larger than those in the other scenarios, which was consistent with the relatively high spatial correlation in scenario I induced by homogenous cell type composition in each spatial domain as compared to the relatively low spatial correlation in the other scenarios due to the heterogenous cell type compositions in each domain. Note that while both HMRF and BASS use the Potts model that contains the interaction parameter β, the β parameter is not directly comparable between the two methods due to the additional hierarchical modeling components introduced in BASS.*

5. Some typos exist in the manuscript.

*Response: Thank you. We have carefully read through the manuscript multiple times and corrected typos.*

Point-by-point Responses to Reviewer 2

Reviewer #2: Here, Li and Zhou present BASS, a computational tool for spatial domain detection and unsupervised clustering of spatial transcriptomics studies. In recent years, technologies for high-throughput, high-resolution profiling of the transcriptome have become more popular. The advantage over traditional scRNA-seq is obvious as spatial information is provided in addition to transcriptome profile. However, to take full advantage of these datasets, novel computational methods are required. BASS addresses one of the central challenges in scRNA-seq - unsupervised clustering - as well as the closely related problem of identifying domains in spatial data. As there are currently several complementary technologies available, there are additional challenges for developing computational methods as one would ideally like each method to be applicable across multiple technologies. The authors demonstrate that BASS outperforms existing methods across several platforms which is crucial for this type of method. I would like for the authors to address the following major issues.

*Response: Thank you for your positive and constructive comments. Our detailed responses to your specific comments are listed below.*

1. Also take run time and memory usage into consideration when reporting on the performance of the different methods. In particular, users will be interested in learning how well methods scale with the size of the data.

*Response: Thank you for the comment. We have included the running time and memory usage of BASS for all the real data applications along with the corresponding sample sizes in these data in Supplementary Table S1. Overall, BASS is comparable to the other spatial domain detection methods and cell type clustering methods in terms of both running time and memory usage. For a typical 10x Visium data with about 5,000 spots, BASS takes about 8 minutes and uses 2 gigabytes of memory. In addition, both the running time and memory usage of BASS scale linearly with the sample size of the data, which makes BASS scalable to analyzing tens to hundreds of thousands of cells/spots. We have included this information to the Results section (lines 931-937 on page 31).*

2. Although the ARI is a useful metric for evaluating clustering performance, it is not without flaws. One particular issue is the fact that it can give a relatively small penalty if small cluster of cells is miscategorized. However, from a biological point of view this can be undesirable as there are many applications where one is interested in rare cell populations. Thus, I would like for the authors to comment on how BASS (and other methods) perform with regards to small clusters. It would be ideal if this aspect was investigated as part of the simulations. I think the setup of the simulations is nice, but the main shortcoming is that they only contain four cell types which is not realistic for most real use cases.

*Response: Thank you for the comment. Following your suggestion, we have added a new simulation scenario (scenario V) to evaluate the performance of all methods in the presence of rare cell types. The simulation details are provided in the updated Methods and material section (lines 327-341 on pages 12-13). Specifically, we assumed that the tissue consisted of four major cell types along with either six or ten rare cell types. We assumed that the major cell types consisted of 70% of cells in the data while the rare cell types consisted of the remaining 30% of cells. This way, each rare cell type consisted of 5% (~ 56 cells) or 3% (~ 34 cells) of the total cell population. We assumed that the composition of major cell types in different spatial domains was the same as that in the scenario III, where each spatial domain contained three major cell types with a 2:1:1 ratio. For rare cell types, we examined two settings where the rare cell types exhibit either a random distribution pattern or a domain-specific pattern. In the setting of a random distribution pattern, we assumed that the rare cell types were randomly distributed across the entire tissue. In the setting of a domain specific pattern, we assume that each rare cell type was located only in one spatial domain. In these rare cell type simulations, in addition to ARI, we also evaluated the performance of all methods using the F_1 score and Matthews correlation coefficient (MCC) following previous papers on rare cell type clustering [20-22]. The new results are displayed in the Supplementary Figures S18-S19 and described in the Results section (lines 643-666 on pages 22-23). In the analysis, we found that the performance of all methods decreased when we increased the number of rare cell types from six to ten, although the performance of BASS remained much better than the other methods (Figure S18). For example, when the rare cell types were randomly distributed across the entire tissue, the median F_1 scores (MCCs) of BASS, SC3, Seurat, and FICT decreased from 0.78 (0.78), 0.63 (0.63), 0.57 (0.55), and 0.40 (0.37) to 0.51 (0.50), 0.40 (0.39), 0.35 (0.34), and 0.27 (0.25), respectively, when the number of rare cell types increased from six to ten. On the other hand, the median ARI from BASS only slightly decreased from 0.84 to 0.76 when the number of rare cell types increased from six to ten, indicating that BASS can still accurately detect the major cell types and that the ARI is not an appropriate metric for evaluating the clustering of rare cell types. In contrast, the median ARI from SC3, Seurat, and FICT decreased substantially from 0.65, 0.56, and 0.43 to 0.34, 0.43, and 0.35, indicating their performance on detecting major cell types is substantially impacted by the presence of rare cell types. In addition, the performance of BASS on detecting rare cell types improved when rare cell types exhibit a domain specific pattern (Figure S18). For example, when the number of rare cell types was set to be ten, the median F_1 score (MCC) of BASS increased from 0.51 (0.50) to 0.59 (0.59) when the distribution patterns of rare cell types changed from random to domain specific. In contrast, the performance of SC3, Seurat, and FICT remained largely similar. Finally, we found that the performance of all methods on detecting spatial domains remained largely similar regardless of the number of rare cell types and slightly increased when rare cell types exhibit domain-specific distribution patterns, presumably because spatial domains are largely characterized by major cell types and thus their detection is not influenced much by the presence of rare cell types (Figure S19).*

3. Similarly, sometimes the ARI can be overly pessimistic. For example if a large cluster is split into two this can result in a poor score when in reality it may not be such a bad decision. I would like for the authors to check if this is the case for any of the methods and datasets to make sure that erroneous conclusions are avoided.

*Response: Thank you. Following your suggestion, we carefully examined the settings where a large cluster was split into two or more clusters in both simulations and real data applications. In the simulations, we have performed additional experiments where we specified the number of cell types and the number of spatial domains to be higher than the underlying truth, which will lead to the situation where a true cluster may be split into two or more clusters (details in the response to reviewer #1’s second comment). In this case, splitting one cluster into multiple clusters, especially multiple clusters of similar sizes, is not ideal and could lead to a loss in ARI. In the analysis, we indeed found that, when the number of cell types or spatial domains was over-specified, cells belonging to the same type or domain could be split into two or more clusters, leading to a decrease in ARI (Figures S16A-C and S17A-C). Among the compared methods, however, we also found that BASS was relatively robust to such over-specification in the number of cell types. In particular, the redundant clusters from BASS comprised only a small percentage of the total cell population (4%-6% for cell type clustering and 0.2%-0.6% for domain detection), leading to only a small decrease in ARI for BASS (0.027-0.051 for cell type clustering and 0.001-0.003 for domain detection). In both STARmap and MERFISH datasets, similar to what we have observed in simulations, we also found that some cell types were split into multiple cell type clusters, especially by the other methods. For example, in the STARmap dataset, Seurat, SC3, and FICT all produced NA clusters with no clear marker gene expression (Figure S23B-D). This is likely because cells of different cell types were somehow split from their main clusters and then combined together. In the MERFISH dataset, we also noticed that Seurat has split the astrocytes into two clusters (Astro-1 and Astro-2; Figure 7C) with very similar overall gene expression pattern (Figure S27B). We performed a careful differential expression analysis and identified a few genes that displayed very subtle expression differences between the two clusters (Figure S28). Therefore, it is possible that the two clusters may represent two astrocyte subtypes with very similar expression patterns. We have updated the manuscript in the real data results section (lines 600-641, 737-739, 827-832 on pages 21, 22, 25 and 28) to reflect the above discussions.*

4. Different technologies will typically differ in terms of the number of transcripts that they are able to profile, ranging from ~100 to the full transcriptome. It would be interesting to understand how the performance of BASS (and other methods) depends on the number of genes used. Could the authors carry out simulations where genes (rows) are randomly excluded from the count matrix to understand how many genes are required for good performance? Related to this point, is there some way that the method can inform the user which genes are most informative in terms of the clustering? This information could be very helpful to users interested in developing novel gene panels for future experiments.

*Response: Thank you for the comment. Following your suggestion, we have added a new simulation analysis in which we randomly excluded genes from the gene expression matrix and evaluated its influence on the performance of all methods. The new simulation is described in detail in the Methods and material section (lines 343-350 on page 13). Specifically, we focused on the setting where the number of genes (nGenes) was set to be 1,000 and where the other parameters were set to be the baseline (de.prob=0.2 and de.facloc=1.1) for all four simulation scenarios. We randomly excluded genes from the simulated expression count matrix and evaluated settings where 100, 200, or 500 genes were retained. The new results are displayed in the Supplementary Figure S20 and described in the Results section (lines 668-690 on pages 23-24). As expected, for both cell type clustering and spatial domain detection, the performance of all methods decreased when fewer genes were retained in the expression matrix, though the performance of BASS remained better than the other methods. For cell type clustering, the performance of BASS was also less affected by a random exclusion of genes than the other methods (Figure S20A). For example, in scenario III, the median ARI of SC3, Seurat, and FICT decreased substantially from 0.92, 0.88, and 0.93 to 0.27, 0.34, and 0.40 while the median ARI of BASS decreased from 0.97 to 0.74 when the number of retained genes decreased from 500 to 200. In addition, the performance of BASS on cell type clustering was more robust to the random exclusion of genes in scenarios I and II than in scenarios III and IV while the performance of SC3, Seurat, and FICT remained largely similar across the four scenarios, suggesting that BASS has advantages over the other three methods on detecting cell types that display strong spatial patterns (Figure S20A). For spatial domain detection, we found that both BASS and the oracle version of HMRF were more robust to a random exclusion of genes than BayesSpace and SpaGCN (Figure S20B). For example, in scenario III, when the number of retained genes decreased from 500 to 200, the performance reduction in terms of ARI was 2% and 20% for BASS and HMRF while the performance reduction was 88% and 63% for BayesSpace and SpaGCN. We also noticed that the performance of BASS on spatial domain detection varied a lot in scenarios III and IV compared to scenarios I and II. Presumably, this is because spatial domains cannot be accurately detected when cell types are not well clustered, which is more likely to occur in scenarios III and IV as mentioned above.*

*For your second comment on identifying clustering informative genes, BASS does not explicitly perform gene selection for clustering and thus does not directly provide such information at the moment. However, we note that two strategies can be adopted to identify cluster informative genes. First, we can apply a post-hoc differential expression analysis to identify both cell type marker genes and domain marker genes based on the corresponding cell type or spatial domain estimates from BASS. We applied this strategy to both STARmap and MERFISH datasets. There, we found that each cell type/spatial domain exhibit a unique expression signature, allowing us to identify important gene markers for each cell type/spatial domain (Supplementary Figure S32). For example, in the MERFISH dataset, we identified top marker genes Cd24a and Nnat for V3 domain; Cyp19a and Scgn for BST domain; Mbp and Ermn for fx domain; Tac2 for MPA domain; Calcr and Nts for MPN domain; Slc18a2 and Scg2 for PV domain; Gal and Th for PVH domain; and Necab1 and Ramp3 for PVT domain (Supplementary Figure S33). Second, we can modify BASS to take advantage of its normal-gamma prior on the mean parameters of the gene expression (details in the Supplementary notes on pages 37-38) for informative gene selection. Specifically, the feature-specific scaling factor (λ_j) in BASS reflects how informative the jth feature is in distinguishing different cell types, with a larger λ_j indicating a more informative expression feature. Therefore, when the gene expression matrix is used as input for BASS, we can identify cluster informative genes by inspecting the parameter λ_j. We followed this idea and applied BASS to the BZ5 sample in the STARmap data set. Indeed, genes with large λ_j estimates are marker genes of different cell types (Figure S31). For example, Bgn and Mgp, which had the highest λ_j estimates, are marker genes of the smooth muscle cells; Sst, Pnoc, Npy, Vip are marker genes of different inhibitory neurons; Itgam is a marker gene of the microglial cells; and Pcp4 is a marker gene of the excitatory neurons in layer 6 (eL6a and eL6b) (Figure S31B). We have included paragraphs to discuss both strategies in line of above in the Results section (lines 876-896 on pages 29-30) and Discussion section (lines 1015-1029 on page 34).*

5. In fig 8 the ARI results are shown as a jitter plot, including the results from multiple sections. It is not clear to me why the results in fig 4 & 6 were not displayed in a similar manner as there are clearly several sections available.

*Response: Thank you for pointing out this discrepancy. In the Figure 8, we benchmarked different methods on spatial domain detection with the DLPFC dataset because a manual annotation of cortical layers has been provided as the underlying true domain labels for all the twelve samples in this dataset. However, the original STARmap [23] and MERFISH [2] papers did not provide such information. Therefore, in the single-sample analysis of STARmap and MERFISH datasets, we focused on one sample in each dataset and manually annotated the sample with spatial domain labels, allowing us to quantitively evaluate different methods with ARI. There, the manual annotation of spatial domains was based on the spatial expression of marker genes and the histology diagram of the mouse brain from the Allen’s brain atlas [24] following previous papers [25, 26]. Such manual annotation is labor-intensive, so we previously only annotated one tissue section. Therefore, previously in the multi-sample analysis of STARmap and MERFISH datasets, instead of ARIs, we simply relied on a visual inspection to evaluate the performance of different methods. There, we have identified consistent spatial domains across tissue sections in each dataset (Figures 4H and 6H). In particular, the varying shapes and sizes of spatial domains across the consecutive sections from MERFISH dataset provided insights into the structural organization of the preoptic region of hypothalamus (Figure 6H).*

*Following your comments, we manually annotated all the remaining samples in the STARmap and MERFISH datasets (two for STARmap and four for MERFISH dataset) with spatial domain labels based on the spatial expression of marker genes and the histology diagram of the mouse brain from the Allen’s brain atlas. The manual annotations were uploaded to our GitHub repository (https://github.com/zhengli09/BASS-Analysis) to facilitate future research in the community. With these annotations, we reported ARIs of different methods on spatial domain detection for these samples. Similar to what we have found in the sample of focus, BASS detected spatial domains that largely resemble the underlying annotations, more so than the other methods. In addition, the multi-sample integrative analysis of BASS further improved its performance on spatial domain detection and produced consistent spatial domains across samples. For example, in the single-sample analysis of the MERFISH dataset, the ARIs from BASS are 0.46 (Bregma-0.04), 0.54 (Bregma-0.09), 0.58 (Bregma-0.14), 0.64 (Bregma-0.19), and 0.67 (Bregma-0.24) while in the multi-sample analysis, the ARIs from BASS are 0.55 (Bregma-0.04), 0.65 (Bregma-0.09), 0.58 (Bregma-0.14), 0.69 (Bregma-0.19), and 0.68 (Bregma-0.24). We updated the corresponding Methods and material section (lines 425-426 and 459-460 on pages 15 and 16) and included the new results in the Supplementary Figures S22 and S26 and the Results section (lines 723-726 and 811-814 on pages 25 and 27).*

6. I find it interesting that BASS shows drastically better performance compared to HMRF and BayesSpace, even though all three methods are based on the Potts model. I would like the authors to comment on this in the discussion and provide their interpretation of what aspect of their model is responsible for the improvements.

*Response: Following your suggestion, we have included a paragraph of discussion to explain the methodological advantages of BASS over HMRF and BayesSpace in the Discussion section (lines 947-970 on page 32). Briefly, while all three methods employ a Potts model, BASS introduces additional hierarchical modeling structures on top of the Potts model to allow for flexible and effective spatial transcriptomic modeling. The additional hierarchical modeling structures allow BASS to make fundamentally different and more effective assumptions on the composition of spatial domains as compared to HMRF and BayesSpace. Specifically, both HMRF and BayesSpace define the spatial domain to be a region with a homogenous gene expression and model the gene expression of all cells in a spatial domain with the same distribution, either a multivariate normal distribution for HMRF or a multivariate t-distribution for BayesSpace. However, spatial domains are often composed of multiple cell types characterized by distinct gene expression profiles. Modelling gene expression in a spatial domain with the same distribution can be insufficient for capturing the gene expression heterogeneity across cell types. Therefore, BASS introduces the cell type composition as an intermediate layer in the hierarchical modelling structure to explicitly model distinct gene expression of different cell types (Eq.(1) at line 164 on page 7). Consequently, BASS is able to define a spatial domain to be a region with a unique cell type composition (Eq.(2) at line 164 on page 7) to better capture the gene expression heterogeneity within each spatial domain and achieve improved performance. In addition, BASS infers the spatial interaction parameter β in the Potts model based on the data at hand while HMRF and BayesSpace fix the parameter to a user-specified value. The inference of β in BASS also contributes to its improved performance. Finally, from the model inference perspective, although both BASS and BayesSpace are based on a Bayesian framework, BASS uses the Swendsen-Wang algorithm to sample the spatial domain labels while BayesSpace uses a Gibbs sampling algorithm. The Swendsen-Wang algorithm is known to have a much better mixing rate than the Gibbs sampling algorithm, thus also helping BASS to achieve improved accuracy [27].*

7. What happened for some of the points in 3d for 5 and 10 slices? These are clearly outliers, so I wonder if the authors could analyze why their algorithm has failed here? Except for those examples, the method performs extremely well for all four scenarios and this worries me a bit. In particular since the performance is never this good for any of the real datasets, I am concerned that the simulated data is a bit too easy here. I would like for the authors to alter some of the parameters to ensure that the simulations become more challenging and hence more representative of real data.

*Response: Thank you for pointing out those outlying cases. We investigated those cases and found that the issue arose from an inaccurate estimation of the spatial parameter β in the Potts model in those cases due to an insufficient number of MCMC samples. Specifically, the BASS software currently implements a stopping rule to automatically determine the number of MCMC samples required for model fitting. In particular, we calculate the mean of the sampled β in every 100 iterations and stop sampling if the difference in the consecutive two means is below a certain threshold. Such stopping rule can help improve computation efficiency and works well in almost all cases except for those few outlying cases. After a careful examination of those outlying cases, we found that all those cases were caused either by an insufficient number of burn-in iterations (2,000 by default in the previous version) or a relatively loose threshold for the stopping rule (0.1 by default in the previous version). After specifying a larger number of burn-in iterations (5,000) and a more stringent threshold (0.001) for the MCMC, we found that BASS could accurately detect spatial domains in all those outlying simulation replicates. Given these observations in the simulation study, we have decided to change the default threshold in BASS to 0.001. In addition, in cases where the default number of MCMC iterations is not sufficient to reach the stopping threshold, we have also incorporated a warning message into the software to encourage users to manually inspect the trace plot of β to determine if a larger number of burn-in iterations is needed. We have updated all the simulation results in Figure 3 and provided the trace plots of β for all our real data applications in the Supplementary Figure S21. The results did not change for all the other simulation replicates, nor the real data, given that our earlier choice of 2,000 MCMC iterations was sufficient for all those datasets.*

*For your second point on more challenging simulation settings, we have created additional, more challenging, simulations to evaluate the performance of different methods. First, following your comment #2, we have evaluated the performance of BASS and the other methods in the presence of rare cell types in the simulations (details in the response to your comment #2; Figures S18-S19). There, for both cell type clustering and spatial domain detection, we found that the performance of all methods decreased as we have more rare cell types in the data, though the performance of BASS was still much better than the compared methods. In addition, the performance of BASS on clustering major cell types and on detecting spatial domains was robust to the presence of rare cell types, more so than the other compared methods. Second, following your comment #4, we have evaluated the performance of BASS and the other methods when genes were randomly excluded from the expression matrix (details in the response to your comment #4; Figure S20). There, for both cell type clustering and spatial domain detection, the performance of all methods decreased when fewer genes were retained in the expression matrix, though the performance of BASS remained better than the other methods. In addition, the performance of BASS on both cell type clustering and spatial domain detection was more robust to a random exclusion of genes than the compared methods, especially when cell types displayed strong spatial patterns. Finally, we have added one more setting to our main simulation settings where the DE gene strength was set to be a lower value of 0.5, corresponding to an approximately 1.5-fold change in expression between the cell types. The simulation results are displayed in the updated Supplementary Figures S3-S13. As expected, for both cell type clustering and spatial domain detection, the performance of BASS and the other methods decreased when the DE gene strength become weaker, especially in scenarios III and IV where cell types displayed weak spatial patterns (Figures S5-S6 and S9-S10). However, the performance of BASS remained higher than the other methods. The first two sets of new simulations results are described in our responses to your earlier comments while the last set of new simulations results are included in the updated Results section (lines 548-555 on pages 19-20).*

8. I was able to successfully download, install and run BASS using a Nanostring dataset without any problems. As I do not have a ground truth here, it is hard to judge the quality of the results but overall the output seemed very reasonable, so the fact that the method worked on a platform that had not been tested in the manuscript is a very good thing.

*Response: Thank you for trying our method and for the positive comment.*

Minor issues:

1. I find it a little strange that sections 3.5 and 3.6 are named by the method, section 3.7 is named by the brain region rather than the method used. Could the authors explain why this is the case?

*Response: Thank you for pointing out this issue and we apologize for the confusion. We have corrected the names of sections 3.5, 3.6, and 3.7 to include both the tissue type and the spatial transcriptomic technology.*

*References*

*1. Squire LR: Fundamental neuroscience. 4th ed. edn. Amsterdam ; Boston :: Elsevier/Academic Press; 2013.*

*2. Moffitt JR, Bambah-Mukku D, Eichhorn SW, Vaughn E, Shekhar K, Perez JD, Rubinstein ND, Hao J, Regev A, Dulac C, Zhuang X: Molecular, spatial, and functional single-cell profiling of the hypothalamic preoptic region. Science 2018, 362.*

*3. Kim DW, Washington PW, Wang ZQ, Lin SH, Sun C, Ismail BT, Wang H, Jiang L, Blackshaw S: The cellular and molecular landscape of hypothalamic patterning and differentiation from embryonic to late postnatal development. Nat Commun 2020, 11:4360.*

*4. Chiba A: Neuropeptide Y-immunoreactive (NPY-ir) structures in the brain of the gar Lepisosteus oculatus (Lepisosteiformes, Osteichthyes) with special regard to their anatomical relations to gonadotropin-releasing hormone (GnRH)-ir structures in the hypothalamus and the terminal nerve. Gen Comp Endocrinol 2005, 142:336-346.*

*5. Kiss J, Csaba Z, Csaki A, Halasz B: Glutamatergic innervation of growth hormone-releasing hormone-containing neurons in the hypothalamic arcuate nucleus and somatostatin-containing neurons in the anterior periventricular nucleus of the rat. Brain Res Bull 2006, 70:278-288.*

*6. Koutcherov Y, Paxinos G, Mai JK: Organization of the human medial preoptic nucleus. J Comp Neurol 2007, 503:392-406.*

*7. Lawal HO, Krantz DE: SLC18: Vesicular neurotransmitter transporters for monoamines and acetylcholine. Mol Aspects Med 2013, 34:360-372.*

*8. Sloley BD, Kah O, Trudeau VL, Dulka JG, Peter RE: Amino acid neurotransmitters and dopamine in brain and pituitary of the goldfish: involvement in the regulation of gonadotropin secretion. J Neurochem 1992, 58:2254-2262.*

*9. Omeljaniuk RJ, Tonon MC, Peter RE: Dopamine inhibition of gonadotropin and alpha-melanocyte-stimulating hormone release in vitro from the pituitary of the goldfish (Carassius auratus). Gen Comp Endocrinol 1989, 74:451-467.*

*10. Van Goor F, Goldberg JI, Chang JP: Dopamine-D2 actions on voltage-dependent calcium current and gonadotropin-II secretion in cultured goldfish gonadotrophs. J Neuroendocrinol 1998, 10:175-186.*

*11. Trudeau VL: Neuroendocrine regulation of gonadotrophin II release and gonadal growth in the goldfish, Carassius auratus. Rev Reprod 1997, 2:55-68.*

*12. Li Q, Yan Z, Kuang Y, Zhou X, Jin L, He L, Sun X, Tao T, Wang L: Genetic variations in the 3'-untranslated region of SLC18A2 are associated with serum FSH concentration in polycystic ovary syndrome patients and regulate gene expression in vitro. Hum Reprod 2016, 31:2150-2157.*

*13. Fukumitsu K, Kaneko M, Maruyama T, Yoshihara C, Huang AJ, McHugh TJ, Itohara S, Tanaka M, Kuroda KO: Amylin-Calcitonin receptor signaling in the medial preoptic area mediates affiliative social behaviors in female mice. Nat Commun 2022, 13:709.*

*14. Bernstein MN, Ni Z, Prasad A, Brown J, Mohanty C, Stewart R, Newton MA, Kendziorski C: SpatialCorr: Identifying Gene Sets with Spatially Varying Correlation Structure. bioRxiv 2022:2022.2002.2004.479191.*

*15. Pham D, Tan X, Xu J, Grice LF, Lam PY, Raghubar A, Vukovic J, Ruitenberg MJ, Nguyen Q: stLearn: integrating spatial location, tissue morphology and gene expression to find cell types, cell-cell interactions and spatial trajectories within undissociated tissues. bioRxiv 2020:2020.2005.2031.125658.*

*16. Shang L, Zhou X: Spatially Aware Dimension Reduction for Spatial Transcriptomics. bioRxiv 2022:2022.2001.2019.476966.*

*17. Zeira R, Land M, Strzalkowski A, Raphael BJ: Alignment and integration of spatial transcriptomics data. Nat Methods 2022, 19:567-575.*

*18. Jones A, Townes FW, Li D, Engelhardt BE: Alignment of spatial genomics and histology data using deep Gaussian processes. bioRxiv 2022:2022.2001.2010.475692.*

*19. Cho CS, Xi JY, Si YC, Park SR, Hsu JE, Kim M, Jun G, Kang HM, Lee JH: Microscopic examination of spatial transcriptome using Seq-Scope. Cell 2021, 184:3559-+.*

*20. Jiang L, Chen H, Pinello L, Yuan GC: GiniClust: detecting rare cell types from single-cell gene expression data with Gini index. Genome Biol 2016, 17:144.*

*21. Tsoucas D, Yuan GC: GiniClust2: a cluster-aware, weighted ensemble clustering method for cell-type detection. Genome Biol 2018, 19:58.*

*22. Fa BT, Wei T, Zhou Y, Johnston L, Yuan X, Ma YR, Zhang Y, Yu ZS: GapClust is a light-weight approach distinguishing rare cells from voluminous single cell expression profiles. Nature Communications 2021, 12.*

*23. Wang X, Allen WE, Wright MA, Sylwestrak EL, Samusik N, Vesuna S, Evans K, Liu C, Ramakrishnan C, Liu J, et al: Three-dimensional intact-tissue sequencing of single-cell transcriptional states. Science 2018, 361.*

*24. Lein ES, Hawrylycz MJ, Ao N, Ayres M, Bensinger A, Bernard A, Boe AF, Boguski MS, Brockway KS, Byrnes EJ, et al: Genome-wide atlas of gene expression in the adult mouse brain. Nature 2007, 445:168-176.*

*25. Hu J, Li X, Coleman K, Schroeder A, Ma N, Irwin DJ, Lee EB, Shinohara RT, Li M: SpaGCN: Integrating gene expression, spatial location and histology to identify spatial domains and spatially variable genes by graph convolutional network. Nat Methods 2021, 18:1342-1351.*

*26. Maynard KR, Collado-Torres L, Weber LM, Uytingco C, Barry BK, Williams SR, Catallini JL, 2nd, Tran MN, Besich Z, Tippani M, et al: Transcriptome-scale spatial gene expression in the human dorsolateral prefrontal cortex. Nat Neurosci 2021, 24:425-436.*

*27. Zhang XX, Johnson TD, Little RJA, Cao Y: A Bayesian Image Analysis of Radiation Induced Changes in Tumor Vascular Permeability. Bayesian Analysis 2010, 5:189-212.*

**Second round of review**

**Reviewer 1**

The revision has addressed most of my previous concerns. A new question is: in the new simulation on different number of cell types, the use of ARI as a metric to evaluate the clustering performance may not be the best choice as the cluster numbers in the comparisons are not equal. The authors may use other metrics (such as NMI, etc) to present the results.

**Reviewer 2**

The authors have done a thorough job at addressing all of my comments and I believe that the new version should be moved forward for publication

**Authors Response**

**Point-by-point responses to the reviewers’ comments:**

*Point-by-point Responses*

Reviewer #1: The revision has addressed most of my previous concerns. A new question is: in the new simulation on different number of cell types, the use of ARI as a metric to evaluate the clustering performance may not be the best choice as the cluster numbers in the comparisons are not equal. The authors may use other metrics (such as NMI, etc) to present the results.

*Response: Thank you. Following your comment, we have included the normalized mutual information (NMI) as an additional evaluation metric in the simulation study on the influence of the specified number of cell types and spatial domains. The new results are included in the Supplementary Fig. 18 and described in the Results section (lines 272-274 on pages 10-11). The new results with NMI are consistent with our previous results with ARI, further confirming the superior performance of BASS on cell type clustering and spatial domain detection under mis-specified number of clusters or domains.*
